# Synthesis and Biological Activity of Iminophosphine Copper(I) Complexes Derived from 2‑(Diphenylphosphino)benzaldehyde and 4‑Substituted-Benzylamines

**DOI:** 10.1021/acsomega.6c08107

**Published:** 2026-10-28

**Authors:** Alessio Zavaroni, Luca Rigamonti, Giorgio Pelosi, Lorenzo Verderi, Matteo Incerti, Lisa Menegaldo, Diego Montagner, Dominga Rogolino, Francesca Bonvicini, Mauro Carcelli

**Affiliations:** † Department of Chemistry, Life Sciences, Environmental Sustainability, 9370 University of Parma, Parco Area delle Scienze 11/A, 43124 Parma, Italy; ‡ Dipartimento di Scienze Chimiche e Geologiche, 9306 Università di Modena e Reggio Emilia, via G. Campi 103, 41125 Modena, Italy; § Food and Drug Department, University of Parma, Parco Area delle Scienze 27/A, 43124 Parma, Italy; ∥ Department of Pharmacy and Biotechnology, 9296 University of Bologna, Via Massarenti 9, 40138 Bologna, Italy; ⊥ Department of Chemistry, 8798 Maynooth University, W23 X021 Maynooth, Ireland

## Abstract

A panel of copper­(I) complexes was isolated by metal-templated reactions starting from 2-(diphenylphosphino)­benzaldehyde and some 4-substituted-benzylamines, and by changing the ratio between metal and ligand and the anion. The spectroscopic characterization of the copper­(I) complexes was completed by discussion of some structures obtained by single-crystal X-ray diffraction. The copper­(I) complexes were assayed *in vitro* against *Staphylococcus aureus* ATCC 25923, *Escherichia coli* ATCC 25922, and *Candida albicans* ATCC 10231. Cell viability was assessed both in nonmalignant and malignant cell lines, namely Vero and Caco-2 cells, respectively. While the tested compounds show only a nonspecific antibacterial effect against *S. aureus*, some of them showed an interesting antiproliferative activity.

## Graphical abstract

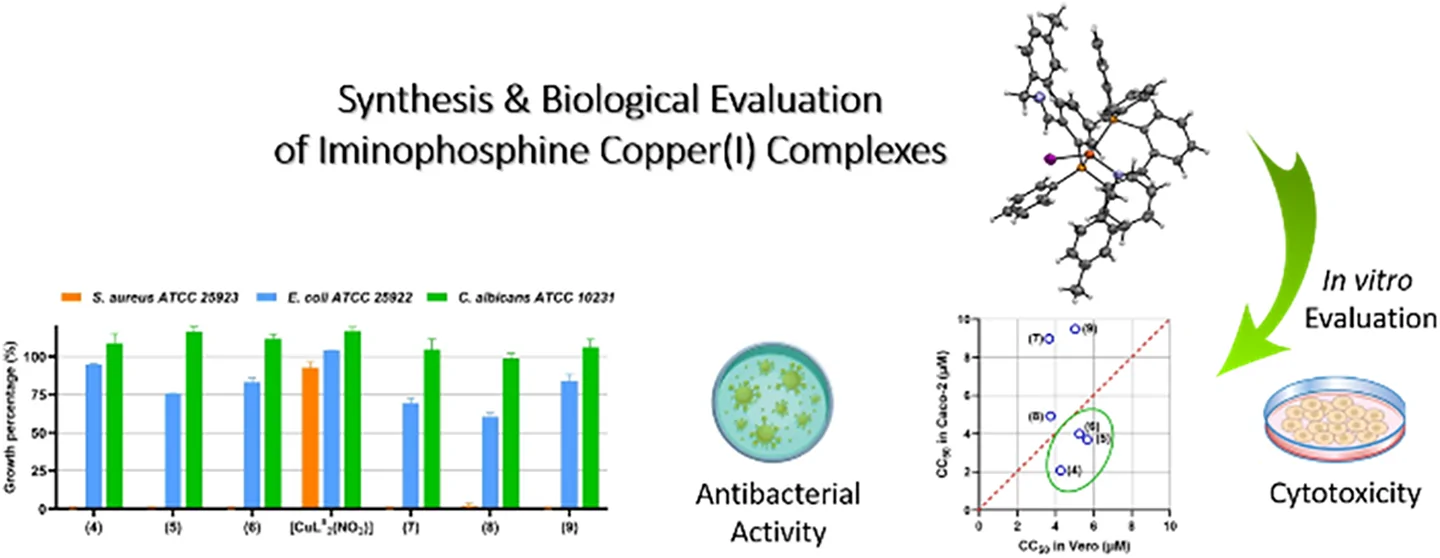

## 1 Introduction

Antimicrobial resistance (AMR) poses a critical threat to global health, as the rapid spread of multidrug-resistant pathogens outpaces the development of traditional antibiotics.^1,2^ Metal complexes have re-emerged as a promising therapeutic alternative: unlike conventional drugs, metallo-antimicrobials utilize multitargeted mechanisms and synergistic metal–ligand activities to circumvent resistance.^3^ Copper complexes are noteworthy for their multifaced biological activity and their ability to induce cell death via different and often interconnected pathways. These mechanisms include DNA damage, reactive oxygen species (ROS) generation as well as cuproptosis, a recently identified form of regulated cell death occurring in both eukaryotic and prokaryotic cells.^4^ This process involves the disruption of copper homeostasis, which targets lipoylated proteins in the tricarboxylic acid cycle and triggers the accumulation of peroxides.^5–7^ By disrupting energy metabolism and promoting oxidative stress, these “cuproptosis-like” mechanisms could offer an opportunity for developing a new generation of copper-based antibiotics.^3^

While the anticancer and the antimicrobial activity of copper­(II) complexes has been deeply investigated, copper­(I) complexes have received much less attention. In fact, copper­(I) coordination compounds have been principally studied for their application in catalysis,^8^ photochemistry^9^ and structural chemistry,^10^ while their biological properties are much less investigated. [Cu­(N–N)­(PR_3_)­(X)] is a class of copper­(I) compounds that raised significant interest (X = I^–^ or NCS^–^, N–N = 2,2′-bipyridyl, 1,10-phenanthroline, 2,9-dimethyl-1,10-phenanthroline or 2,2′-biquinoline, PR_3_ = tris­(aminomethyl)­phosphines).^11^ These species have demonstrated notable *in vitro* anticancer activity, and antimicrobial activity against *Staphylococcus aureus* and *Candida albicans*.^11^ Recently, a novel class of cationic mixed-ligand copper­(I) complexes of type [Cu­(N)­(P)_2_]^+^ was disclosed, where N is a phenoxy-ketimine and P is a monodentate tertiary phosphine. These compounds proved to be active against the Gram-positive *S. aureus* and the Gram-negative *E. coli*.^12^ Some metal­(I)-NHC carbene complexes have been also investigated for their antibacterial activity (NHC, N-heterocyclic carbene, metal = Ag, Cu, or Au): Ag­(I) and Cu­(I) complexes resulted the most efficient at inhibiting biofilm formation.^13,14^

To the best of our knowledge, no biological data are available for copper­(I) complexes with P,N ligands, particularly iminophosphines. These organic molecules have been extensively used for the synthesis of complexes with gold and palladium,^15–17^ while few iminophosphine copper­(I) complexes are reported in literature.^18,19^ Here, a synthetic strategy based on template reactions starting from 2-(diphenylphosphino)­benzaldehyde and some 4-substituted-benzylamines allowed to prepare the copper­(I) iminophosphine complexes **(1)**–**(9)** by varying the metal to ligand *ratio* (1:1 or 1:2) and the nature of the counteranion (Scheme 1).

**Scheme 1:**
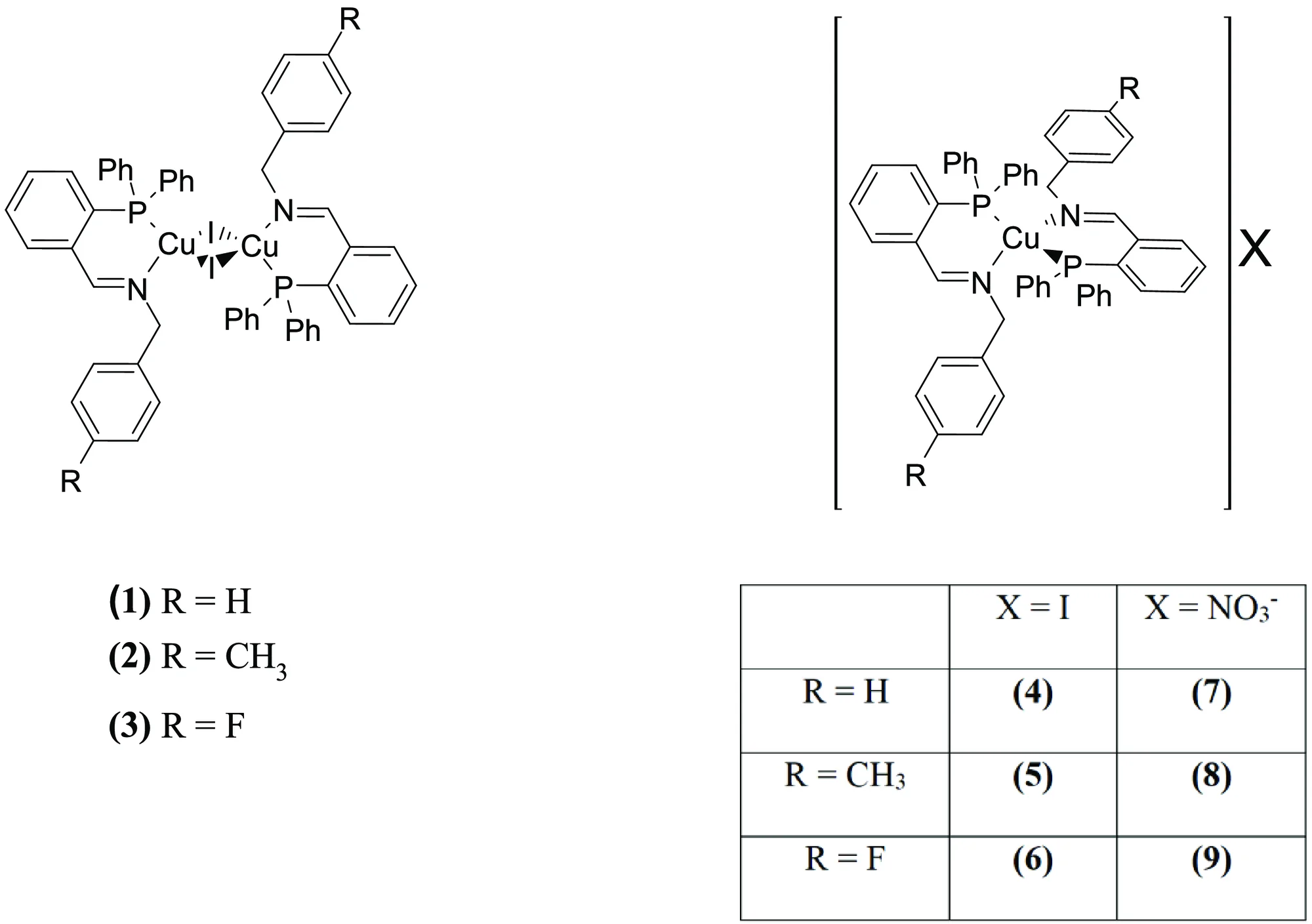
Schematic Representation of the Iminophosphine Copper­(I) Complexes **(1)**–**(9)**

The complexes (**4)**–(**9)**, stable in the assay conditions, were tested *in vitro* against *S. aureus* ATCC 25923, *E. coli* ATCC 25922, and *C. albicans* ATCC 10231, chosen as representative of Gram positive, Gram negative and fungal microorganisms, respectively. Cytotoxicity in malignant Caco-2 cell line was also investigated, and the obtained CC_50_ values were compared to those measured in Vero cells to individuate compounds with anticancer potential.

## 2 Results and Discussion

### 2.1 Synthesis and Characterization of Iminophosphine Copper­(I) Complexes

We started to synthesize some copper­(I) complexes with 2-(diphenylphosphino)­benzaldehyde (PCHO) and with some imine ligands obtained from it, aiming to investigate their antimicrobial and cytotoxic properties. The isolation of the product of the reaction between PCHO and arilamines could be tedious and resulting in poor yields. After several attempts, for example, we obtain N-[2-(diphenylphosphino)­benzylidene]­benzylamine L^1^ with a yield of about 21%. To circumvent synthetic problems, the copper­(I) complexes **(1)**–**(9)** were directly obtained through template reactions. First, copper­(I) iodide was reacted with PCHO and the proper amine (benzylamine, 4-methyl or 4-fluorobenzylamine) in 1:1:1 stoichiometric ratio in acetonitrile/chloroform mixture under inert atmosphere, affording the complexes **(1)–(3)** (Scheme 2).

**Scheme 2:**
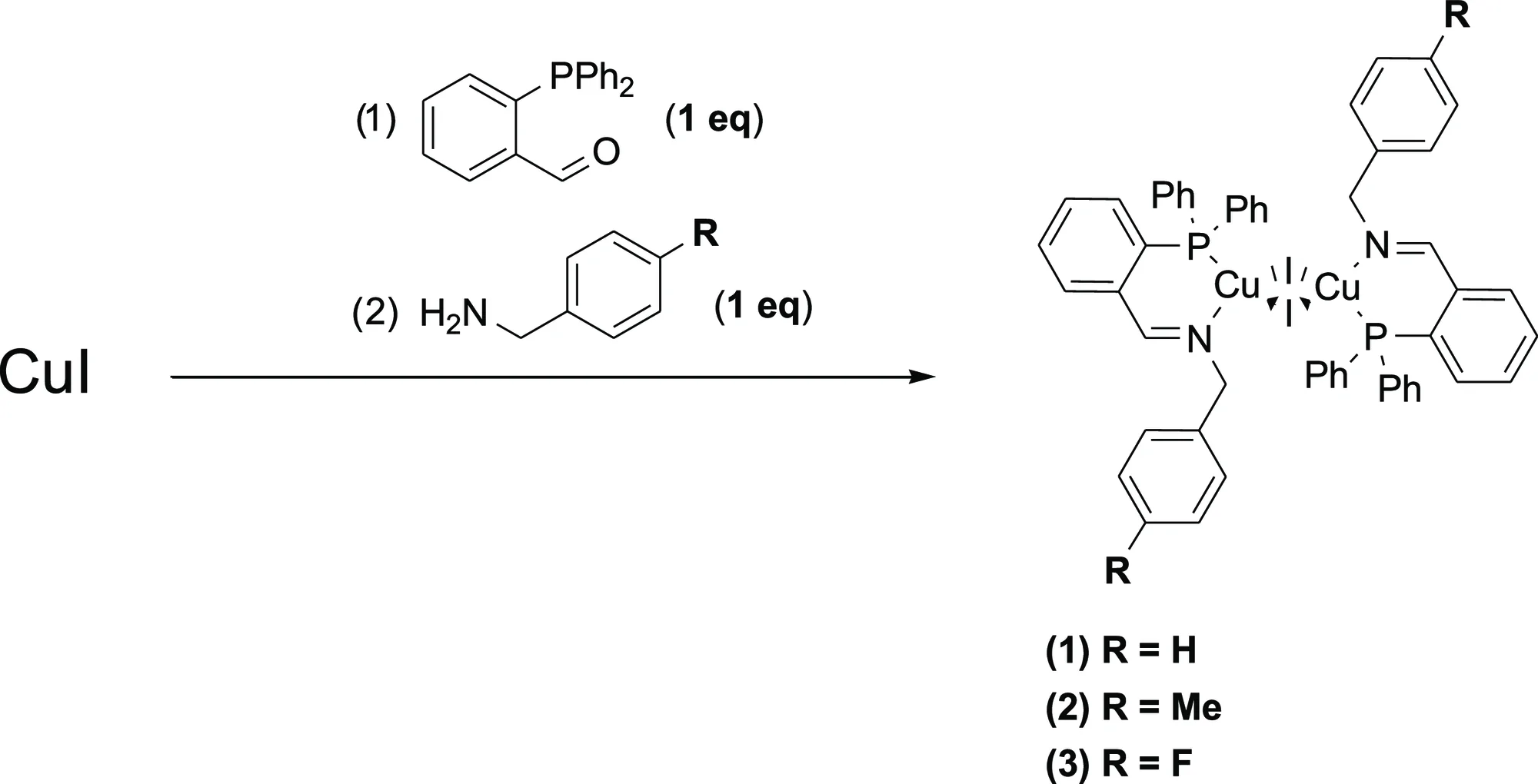
Schematic Representation of the Template Synthesis of the Copper­(I) Complexes **(1)–(3)**. Reagents and Conditions: (1) 1/1 MeCN/CHCl_3_, 50 °C, N_2_, 1 h; (2) MeCN, 65 °C, N_2_, 5 h

The formation of the imine bond was confirmed by IR spectroscopy (appearance of a band at about 1628 cm^–1^ and disappearing of the C=O stretching band of the PCHO reagent, Figures S1–S3). Red-orange single crystals suitable for XRD analysis were obtained by slow evaporation of a solution of **(2)** in CHCl_3_. The crystal data revealed a dimeric structure [Cu­(μ-I)­L^n^]_2_ with each copper­(I) ion coordinated to the P,N-bidentate iminophosphine ligand L^n^ and two iodine atoms (Figure 1 and Figure S4). The iodine atoms bind to the metal centers in a bridging mode, and the phosphorus and nitrogen atoms of the two coordinated ligands adopt a *trans* configuration. The bite angles of the ligands, P1A-Cu1A-N1A and P1B–Cu1B-N1B, are 88.95° and the coordination geometry of copper­(I) is distorted tetrahedral. The Cu1A-Cu1B distance is 2.930 Å, higher than the sum of the van der Waals radii of two copper atoms (2.80 Å), and this excludes any significant metallophilic interaction.

**Figure 1:**
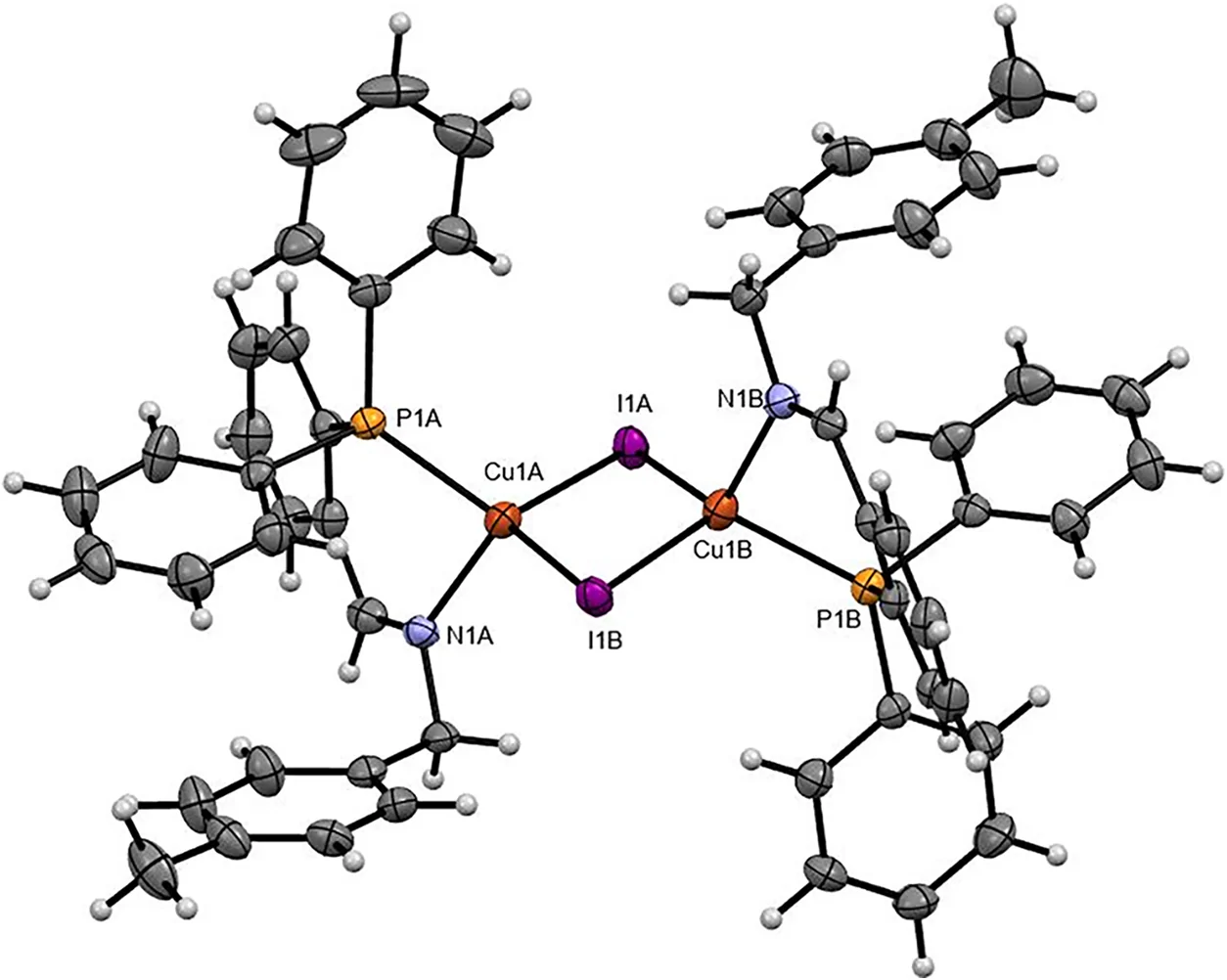
ORTEP diagram of the crystal structure of the complex **(2)**, drawn with 50% probability ellipsoids (phosphorus atoms in light orange, nitrogen in blue, carbon in gray, hydrogens in white, iodine in purple, copper in dark orange).

Single crystal XRD analysis allowed to confirm that **(1)** and **(3)** have the same structure of **(2)**; Figure S5 shows the crystal structures of **(3)** and its crystallographic characterization, while **(1)** was previously reported.^20^

As expected, **(1)-(3)** exhibit similar behavior in solution (Figures S6–S9). In the spectra of the complex **(1)**, for example, both the phosphorus and the imine proton resonances experiment a significant shielding with respect to the free ligand L^1^ (Figure S6a,b). The complexes **(1)-(3)** are stable in CDCl_3_ solution over 3 days, while in a coordinating solvent like DMSO-d_6_, more than one set of signals can be found in the ^1^H NMR spectra immediately after dissolution. After 1 day a peak at 28.77 ppm, attributable to a phosphine oxide, appeared in the ^31^P NMR spectrum (Figure S6c). ESI-MS analyses confirmed the presence of the [CuL]^+^ species in solution (Figures S10–S12).

The 1:1 complex [Cu­(μ-I)­(MeCN)­L^a^]_2_ (L^a^ = PCHO) was also isolated and characterized (Figures S13–S17) as reported in the literature.^21^ The formation of the complex was supported by IR and NMR spectroscopy, where it is evident the shift of the C=O signal respect to the free aldehyde (Figures S13–S15), and by ESI-MS analysis (Figure S16). Figure S17 reports the comparison between experimental powder diffractogram of complex [Cu­(μ-I)­(MeCN)­L^a^]_2_ and the calculated one obtained from literature data,^21^ confirming the structure of the complex.

The synthesis of the 2:1 iminophosphine:copper­(I) complexes **(4)**–**(6)** was carried out as shown in Scheme 3. An excess of aldehyde and 4-fluorobenzylamine amine reagents (up to 3 equiv.) is required to obtain **(6)**, otherwise the previously characterized 1:1 species **(3)** was isolated.

**Scheme 3:**
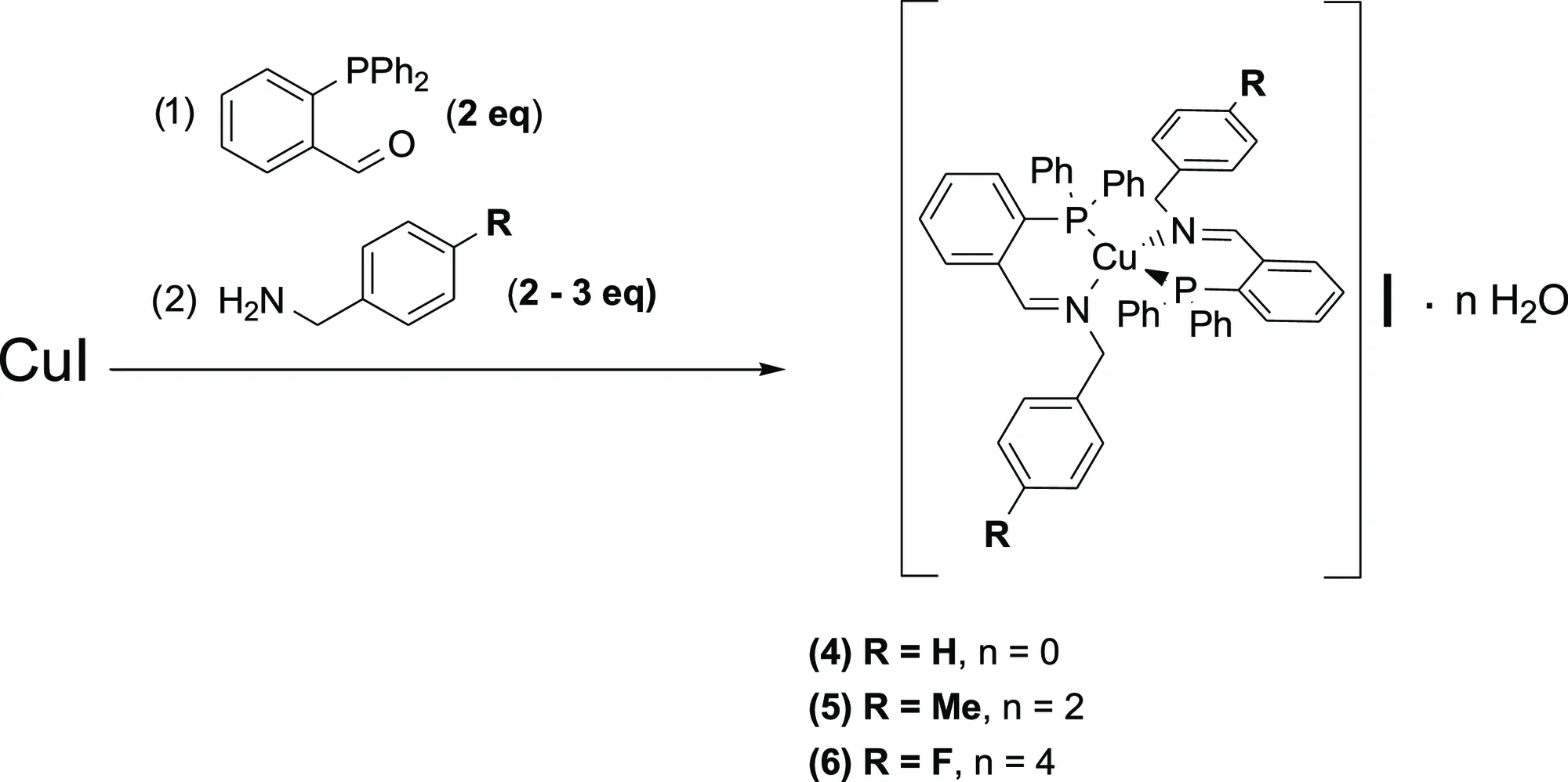
Template Synthesis of the Complexes **(4)–(6)** ^a^

a Reagents and conditions: (1) 1/1 MeCN/CHCl_3_, 50 °C, N_2_, 1 h; (2) MeCN, 65 °C, N_2_, 5 h.

The FT-IR spectra of the complexes **(4)**-**(6)** are similar to the spectra of the corresponding 1:1 ones, with the iminic stretching band at about 1626 cm^–1^ (Figures S18–S20). In the ^31^P NMR spectra of **(4)**-**(6)** there is a single peak, confirming the symmetry of the complexes with the ligands behaving as P,N bidentate (Scheme 3). The presence of only one set of signals for the ligand backbone in their ^1^H NMR spectra also supports this hypothesis. It is interesting to note that **(4)** is soluble in acetonitrile, chloroform and DMSO, contrarily to the dimeric complex **(1)** with the same ligand L^1^. The ^1^H and ^31^P NMR spectra of **(4)** in CDCl_3_, compared with the spectra of **(1)** and L^1^, are shown in Figure S21. The complex **(4)** is stable in CDCl_3_ solution over 1 week, with only traces of formation of phosphine oxide (Figure S22). Contrarily to the 1:1 complexes, **(4) - (6)** are stable also in DMSO-d_6_ (Figures S23–S28). ESI-MS analyses confirmed the presence of [CuL_2_]^+^ species in solution (Figures S29–S31).

After precipitation of the complex **(5)**, slow evaporation of the mother liquors afforded some crystals that, quite surprisingly, were characterized by XRD as the new product **(5a)** (Figure 2 and Figure S32).

**Figure 2:**
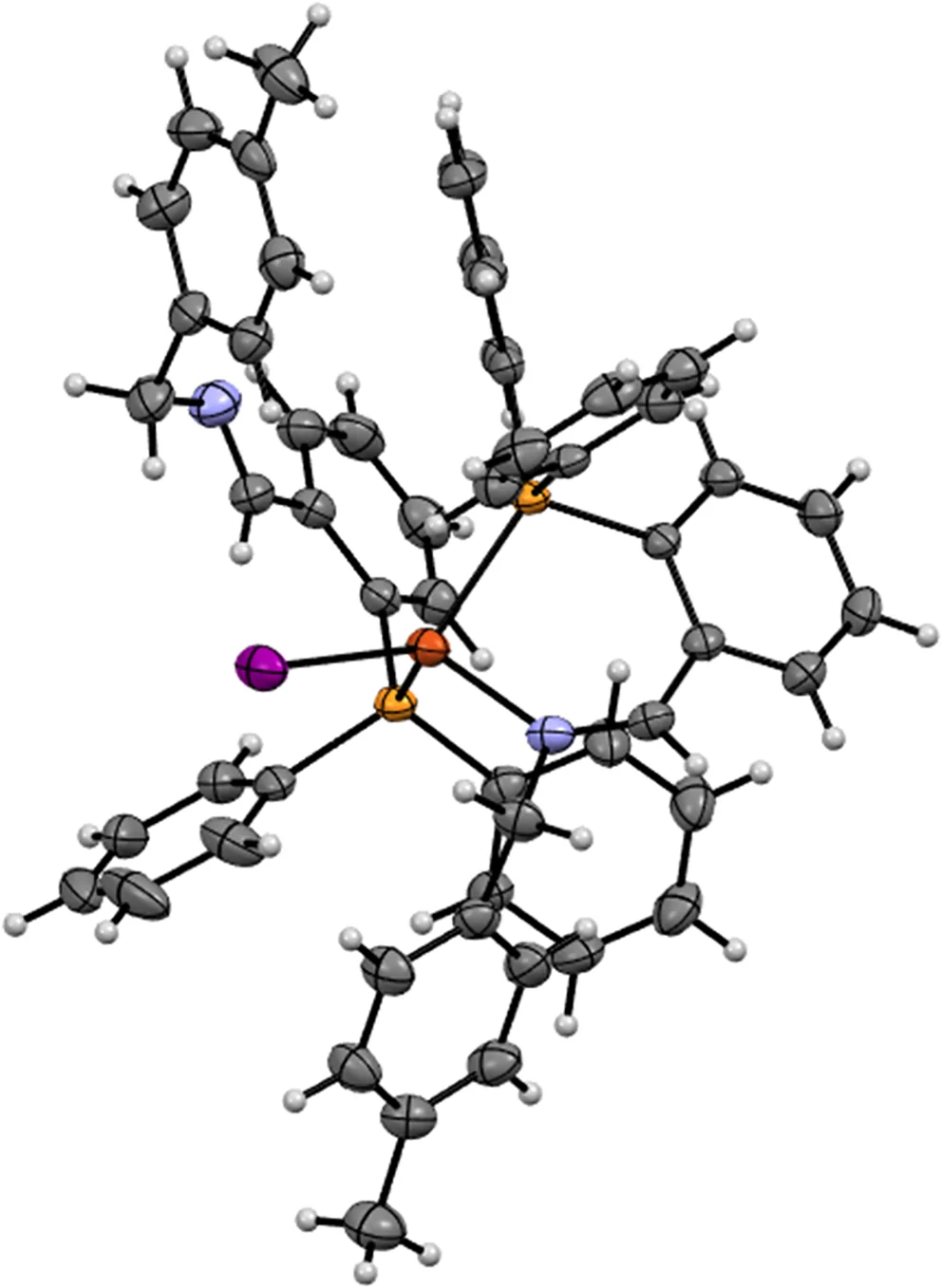
ORTEP diagram of the crystal structure of **(5a)**, drawn with 50% probability ellipsoids (phosphorus atoms in light orange, nitrogen in blue, carbon in gray, hydrogens in white, iodine in purple, copper in dark orange).

In the complex **(5a)**, one ligand acts as P,N-bidentate and the other in P-monodentate fashion; iodide completes the coordination sphere of the copper­(I) ion. The bite angle of the bidentate ligand is 88.38°, while the other coordination angles are larger than 100° giving rise to a distorted tetrahedron. The distance Cu–I is 2.681 Å, the bond lengths of the biting atoms are Cu–N 2.164 and Cu–P 2.306 Å, while the one with the monodentate ligand is consistently Cu–P 2.305 Å.

The complex [Cu­(L^a^)_2_I] (L^a^ = PCHO; Experimental part and Figures S33–35) with a 2:1 ligand:metal stoichiometry was also isolated and characterized.

We also explored the synthesis of the copper­(I) complexes **(7)-(9)** with nitrate as counterion (Scheme 4). They were prepared *via* a comproportionation reaction between copper­(II) nitrate and metallic copper in acetonitrile. [Cu­(MeCN)_4_]­NO_3_ was produced *in situ* and immediately consumed in the template synthesis.^22^

**Scheme 4:**
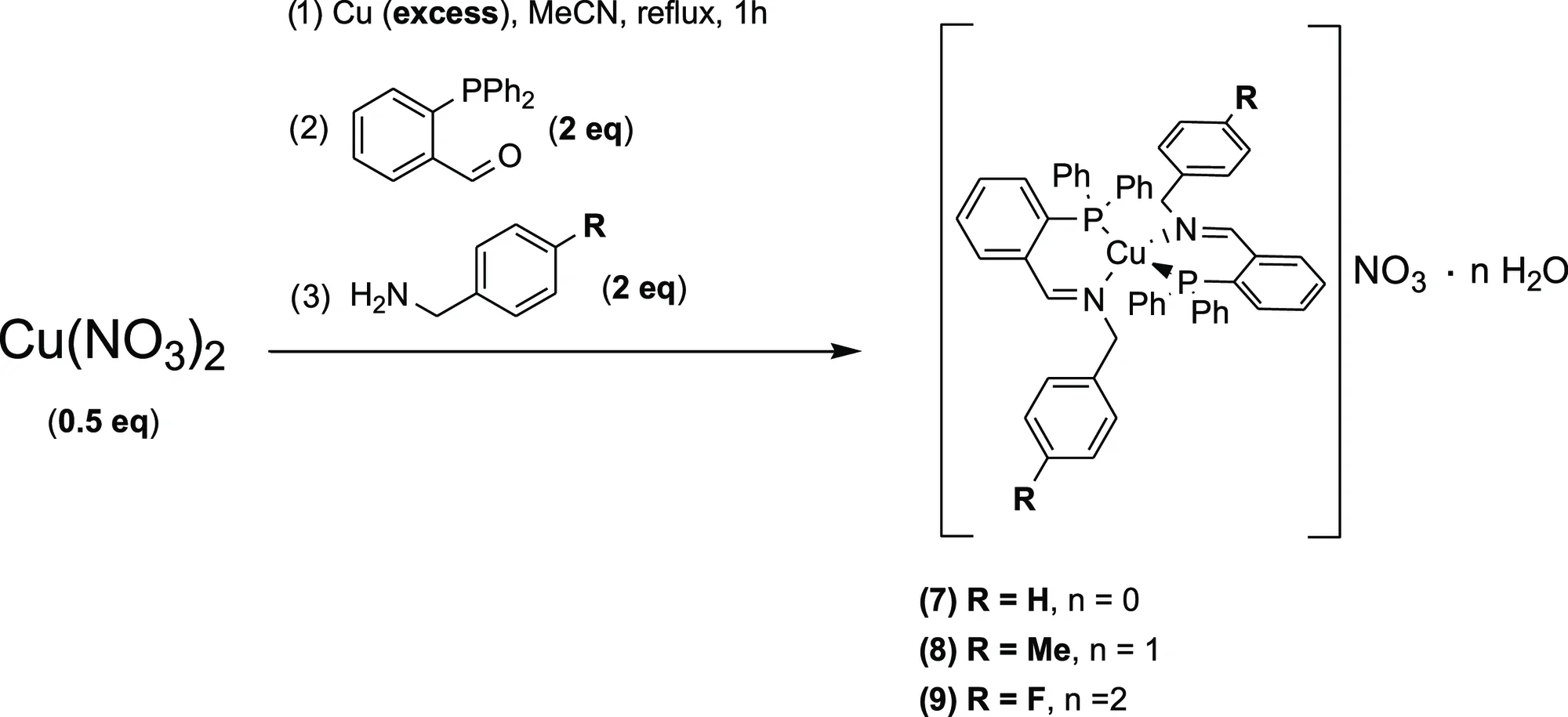
Template Synthesis of the Complexes **(7)–(9)** ^a^

a Reagents and conditions: (1) 1/1 MeCN/CHCl_3_, 50 °C, N_2_, 1 h; (2) MeCN, 65 °C, N_2_, 5 h.

The presence of only one strong band in the FT-IR spectrum in the range 1400 – 1300 cm^–1^ (Section 3 and Figures S36–38) suggests that the complexes **(7)–(9)** are ionic in the solid state.^23^ Single crystals of **(7)** suitable for XRD were obtained via slow evaporation of the mother liquor (Figure 3 and Figure S46). In agreement with the IR spectroscopic data, the crystal structure reveals an ionic framework of type [Cu­(L^1^)_2_]­NO_3_ with a distorted tetrahedral geometry around the copper­(I) center; the imine group of one of the two P,N-bidentate ligands is disordered within the crystal lattice.

**Figure 3:**
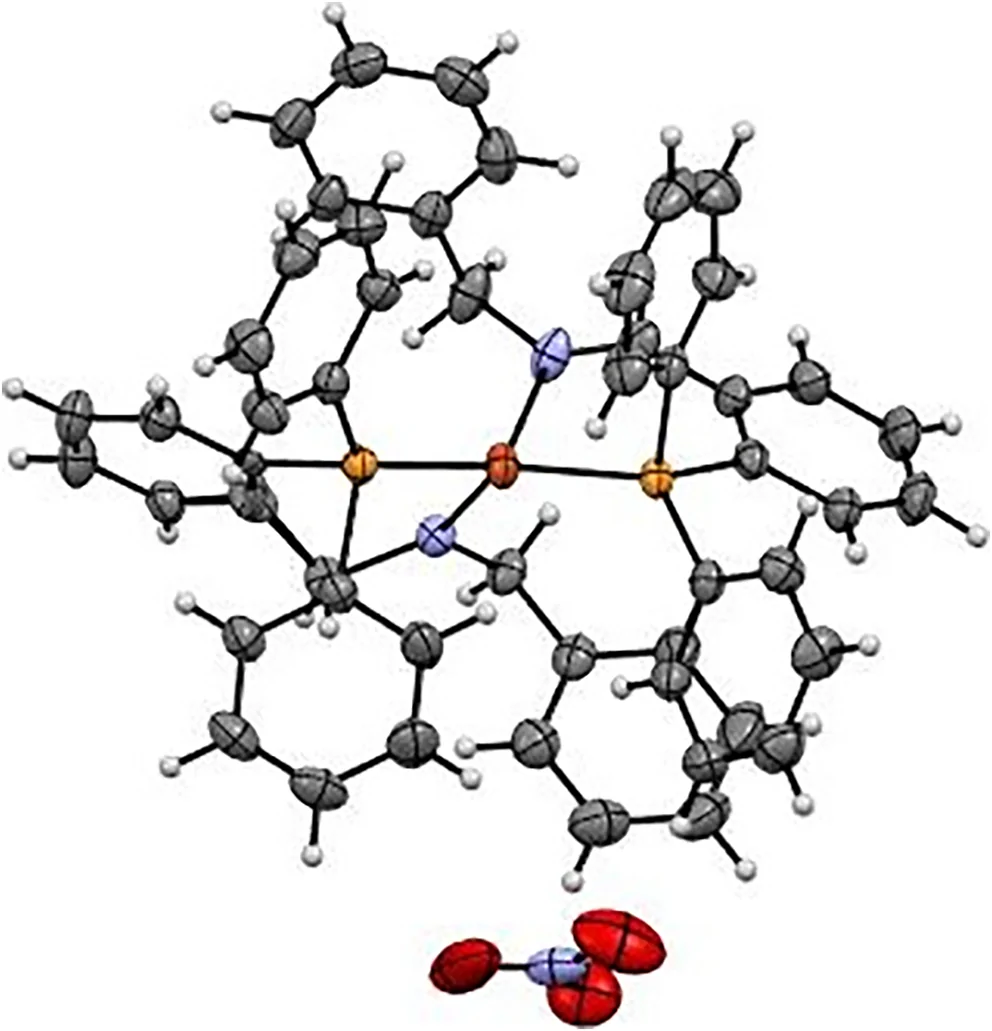
ORTEP diagram of the crystal structure of **(7)**, drawn with 50% probability ellipsoids (phosphorus atoms in light orange, carbon in gray, hydrogens in white, nitrogen in blue, oxygen in red, copper in dark orange). Only one ligand position (occupancy 50%) is represented.

As an example, Figure S39 reports the ^31^P and ^1^H NMR of complex **(7)**. The complexes **(7)–(9)** are stable in DMSO-d_6_ for over 1 week, with no detectable formation of phosphine oxide species (Figure S40–44). ESI-MS spectra are reported as Figure S45.

Finally, the complex **[CuL**_**2**_^**a**^ **(NO**_**3**_ **)]** (Figures S47–50) was isolated and characterized. The FT-IR spectrum shows two strong bands at 1434 and 1286 cm^–1^ ascribable to the N–O stretching of the nitrate group coordinated to the metal center.^24^ Single crystal X-ray diffraction analysis confirmed that the nitrate anion is bidentate, while the two ligands act as P-monodentate (Figure S48). The coordination geometry around the copper­(I) center is a distorted tetrahedron.

### 2.2 Biological Evaluation

The copper­(I) iminophosphine complexes (**4)**–(**9)** and **[CuL**_**2**_^**a**^ **(NO**_**3**_ **)]**, stable and soluble in the testing medium, were assayed *in vitro* against *S. aureus* ATCC 25923, *E. coli* ATCC 25922, and *C. albicans* ATCC 10231. These strains were selected as representative Gram-positive, Gram-negative and fungal microorganisms, respectively, commonly used in studies aimed at evaluating the antimicrobial activity of newly synthesized compounds and other potentially bioactive agents,^24,25^ including copper-based antimicrobials.^26^

The activity of the metal complexes was initially evaluated at a concentration of 100 μM against the three selected microbial species to obtain a preliminary overview of their antimicrobial potential (Figure 4).

**Figure 4:**
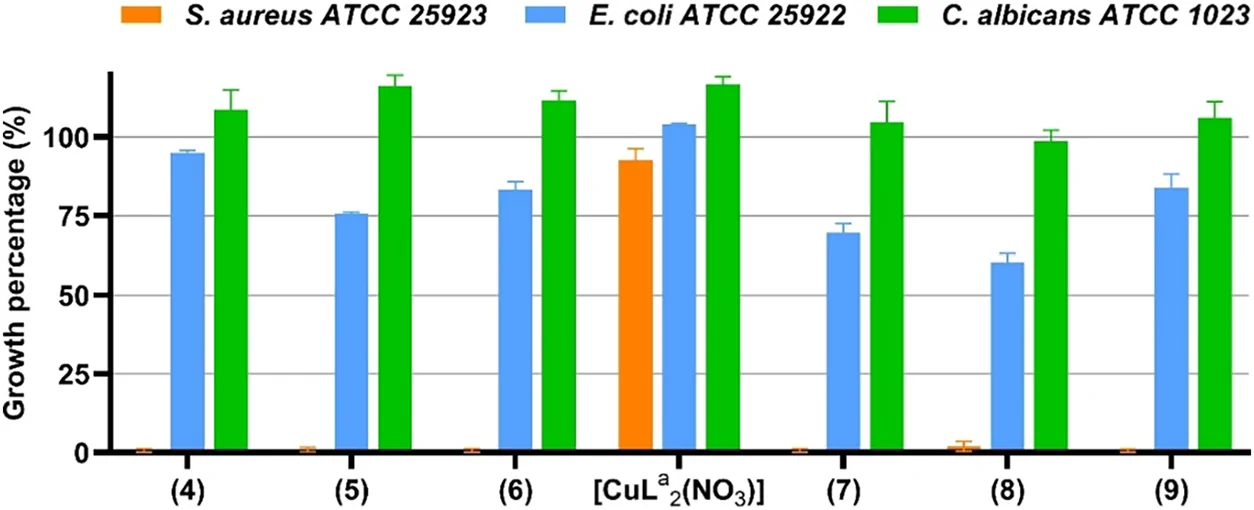
Screening of the antimicrobial activity of copper­(I) iminophosphine complexes **(4)**–**(9)** and **[CuL**_**2**_^**a**^ **(NO**_**3**_ **)]** at 100 μM. Results are expressed as percentage of microbial growth relative to untreated controls (100%). Data represent mean values ± standard deviation.

Based on these results, both iodide- and nitrate-containing complexes appeared to strongly inhibit the growth of *S. aureus*, while **[CuL**_**2**_^**a**^ **(NO**_**3**_ **)]** did not interfere with microbial proliferation. In contrast, none of them exhibited antifungal properties against *C. albicans* as fungal growth remained close to 100% in all cases. Regarding *E. coli*, growth percentages ranged from 60.4% **(8)** to 104.1% **(6)**, however none of the compounds could be considered biologically active against this Gram-negative strain. These differences in susceptibility may be attributed to variations in the cell envelope architecture of the tested species, which constitute distinct permeability barriers and may therefore lead to divergent physicochemical requirements for compounds to exert inhibitory activity against each microbial species.

The panel of metal compounds (**4)**–(**9)** were subjected to dose–response experiments to determine their IC_50_ (half maximal inhibitory concentration) values against *S. aureus* together with their CC_50_ (50% cytotoxic concentration) values in the Vero cell line (Figure 5), leaving aside the inactive **[CuL**_**2**_^**a**^ **(NO**_**3**_ **)]**. These values were also determined for the reference drugs serving as internal controls in the biological assays (Table S51). These cells, which represent a nonmalignant mammalian cell model, are internationally recommended as a standard system for assessing the cytotoxicity of pure compounds as well as a wide range of products and formulations (ISO 10993–5). Under our experimental conditions, the IC_50_ values for *S. aureus* ranged from 19.08 to 27.15 μM (Table S52), whereas the CC_50_ values for Vero cells ranged from 3.52 to 5.67 μM (Table S53), confirming the well-known toxicity of some metal-based compounds.^27,28^ The current iminophosphine substituents imparted minimal variations in bioactivity; expanding this scaffold with groups of contrasting lipophilicity and electronic character will be necessary to establish reliable SAR rules.

**Figure 5:**
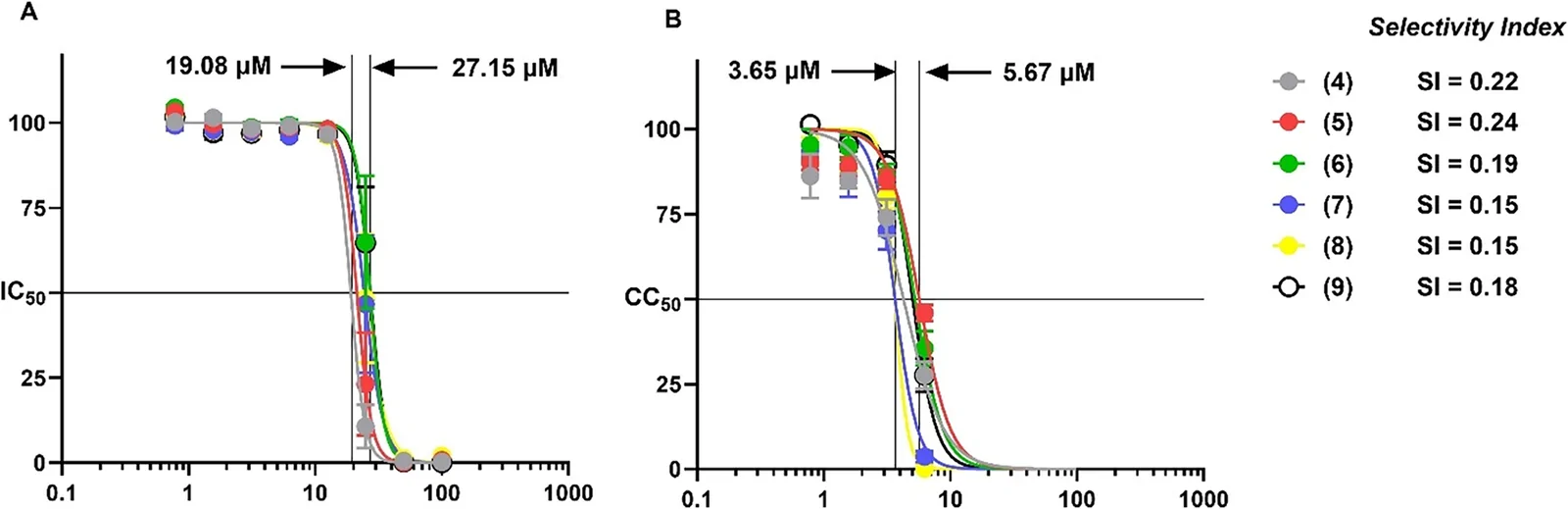
Dose–response curves of the complexes showing (A) antibacterial activity against *S. aureus* and (B) cytotoxicity in Vero cells. Data are expressed as percentage of growth inhibition (A) or cell viability (B) relative to untreated controls and are plotted as a function of the logarithm of compound concentration (μM). IC_50_ and CC_50_ values are indicated by the intersection with the 50% response level. The selectivity index (SI), calculated as CC_50_/IC_50_, is reported for each compound, highlighting their limited selectivity toward bacterial cells over mammalian cells.

The obtained results indicate that the synthesized copper­(I) iminophosphine complexes are more cytotoxic toward mammalian cells than effective against *S. aureus*, as reflected by the selectivity index (SI) values lower than 1. Overall, this profile suggests a lack of selectivity and points to a nonspecific antibacterial effect.

This consideration prompted us to further investigate the biological profile of the copper­(I) iminophosphine complexes in a different cellular context, namely the human colorectal adenocarcinoma-derived epithelial Caco-2 cells, thereby extending the evaluation beyond nonmalignant models. Thus, dose–response curves were generated in Caco-2 cells (Figure S54, Table S55) and the obtained CC_50_ values were compared to those measured in Vero cells (Figure 6). Such comparative analysis identifies three compounds, **(4)**, **(5)** and **(6)**, as possible candidates with an anticancer potential. Differences in cytotoxic potency could be attributed to the counterion, which dictates the complex ionic status, overall lipophilicity, and, probably, efficiency of cellular uptake.

**Figure 6:**
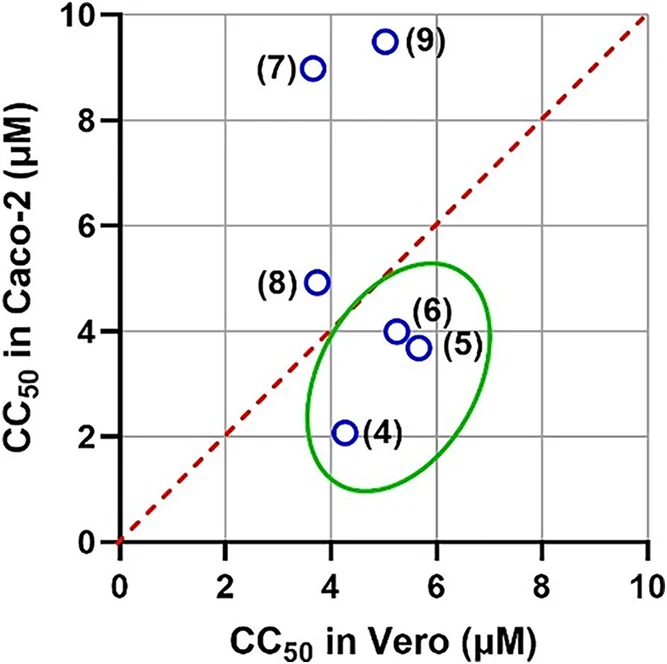
Correlation between CC_50_ values determined in Caco-2 and Vero cells. Each blue circle represents a copper­(I) iminophosphine complex. The red line corresponds to the equality (CC_50_ Caco-2 = CC_50_ Vero). Compounds located below the line exhibit lower CC_50_ values in Caco-2 cells compared to Vero cells, indicating a higher cytotoxicity toward cancer cells and, therefore potential antiproliferative activity. Compounds above the line are more cytotoxic toward nonmalignant cells, suggesting lower selectivity for cancer cells. The green ellipse highlights the compounds that showed antiproliferative activity on Caco-2 cells.

The biological properties of the compounds were further explored using human red blood cells (hRBCs), which serve as a well-established model for evaluating interactions with biological membranes and for assessing preliminary systemic safety in view of potential therapeutic applications. Notably, none of the copper­(I) iminophosphine complexes exhibited significant hemolytic activity at their antiproliferative concentrations (Table S55), suggesting that their biological effect is not associated with nonspecific membrane disruption (Figure 7). Rather, the absence of significant membrane damaging is consistent with the involvement of an intracellular mechanism of action. Such intracellular effects may arise from different molecular pathways.

**Figure 7:**
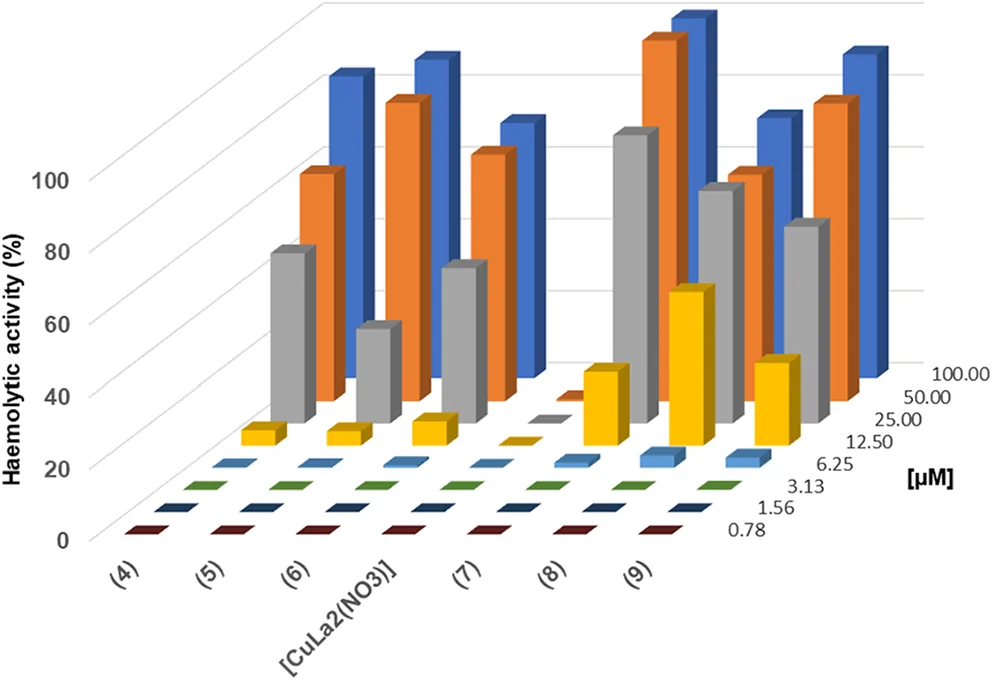
Hemolytic activity of copper­(I) iminophosphine complexes evaluated on hRBCs. Hemolysis is expressed as mean percentage values relative to the positive control (1% Triton X-100, 100% hemolysis), with untreated cells in PBS as the negative control (0% hemolysis).

When a plasmid DNA interaction assay was performed under cell-free conditions, a clear ability to induce damage to plasmid DNA was observed although with different efficiencies (Figure 8). **(4)** and **(8)** showed a strong nuclease effect, with a significant loss of detectable DNA bands, suggesting extensive DNA fragmentation and/or degradation. **(5)**, **(6)** and **(7)** exhibited weaker activity, with less pronounced DNA degradation, while **[CuL**_**2**_^**a**^ **(NO**_**3**_ **)]** and **(9)** did not significantly interact with the plasmid DNA. The changes in the electrophoretic pattern of the plasmid are consistent with an interaction with DNA and, for some complexes (**4**) and (**8**), with substantial DNA damage. This nuclease activity may contribute to the observed reduction in proliferation of both *S. aureus* and mammalian cells. However, alternative mechanisms of action may also contribute to the biological effects observed in these cellular systems, including cuproptosis. Further investigations are therefore warranted to elucidate the molecular pathways underlying the biological activity of these complexes and to assess their potential for therapeutic applications.

**Figure 8:**
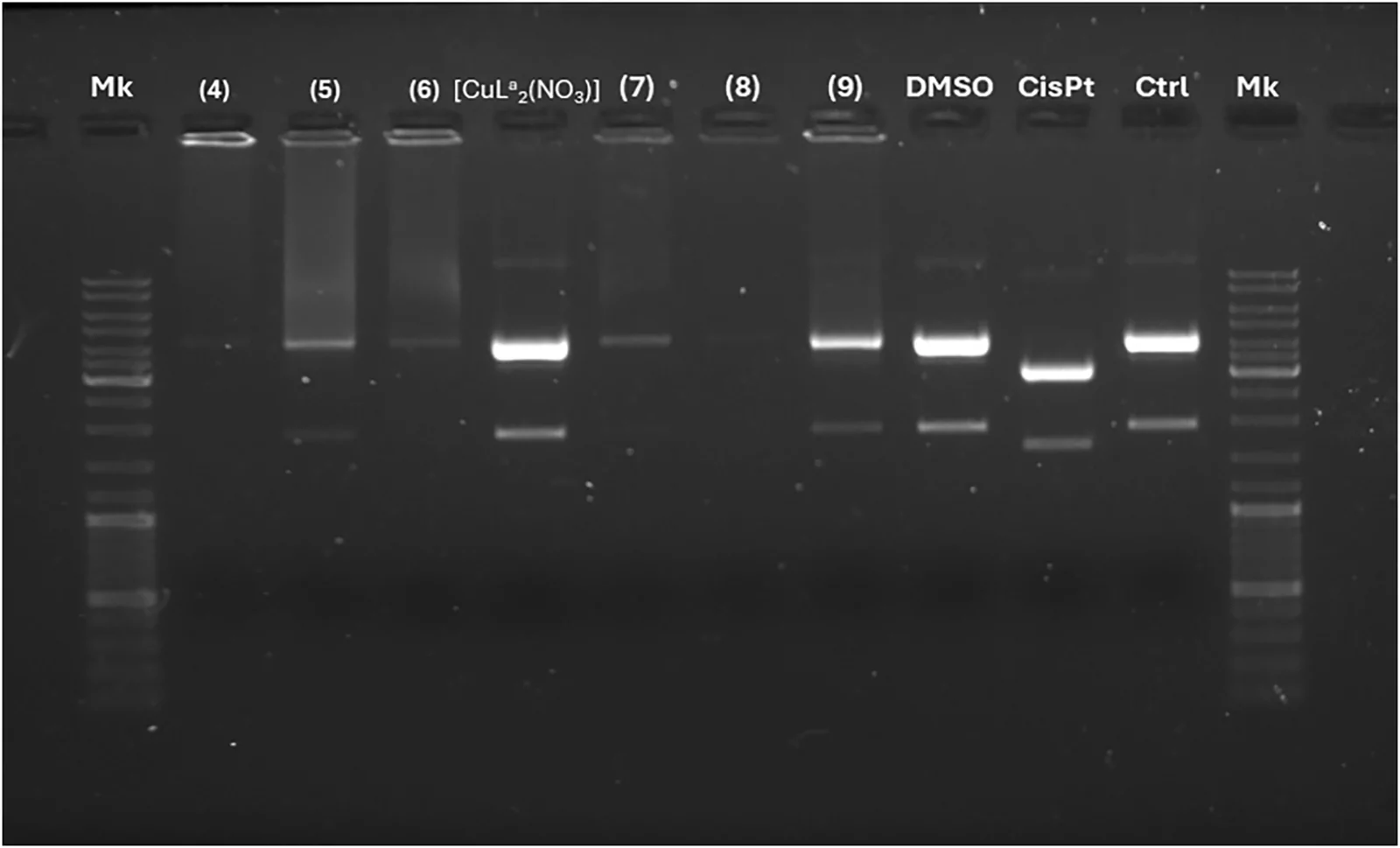
Agarose gel electrophoresis analysis of pmaxGFP (3527 bp) with the metal complexes at 100 μM. Mk: molecular weight marker (Gene Ruler DNA ladder mix); DMSO: solvent control; Ctrl: plasmid DNA control; CisPt (cisplatin) with a different pattern, consistent with its known mechanism to form covalent Pt-DNA adducts and cross-links.

## 3 Experimental Section

### 3.1 Chemistry

Commercial reagents were purchased from Sigma-Aldrich. The purity of the synthesized compounds was determined by elemental analysis and verified to be ≥ 95%. ^1^H, ^13^C and ^31^P and NMR spectra were recorded at 25 °C on a Bruker Advance 400 MHz FT spectrometer or on a Bruker Advance 500 MHz FT spectrometer. The ATR-IR spectra were recorded by means of a PerkinElmer spectrophotometer by using a diamond crystal plate in the range of 4000–400 cm^–1^. Elemental analyses were performed by using a Thermo Fisher FlashSmart CHNS/O analyzer with gas-chromatographic separation. Electrospray ionization mass spectral analyses (ESI-MS) were performed with an ESI time-of-flight Micromass 4LCZ spectrometer. Samples were dissolved in methanol or acetonitrile. Powder X-ray diffraction (P-XRD) data were collected with a Rigaku Smartlab XE diffractometer equipped with a 2D HyPix3000 detector operating in 1D mode. Data were collected under ambient conditions in Bragg–Brentano geometry (Cu Kα λ = 1.5046 Å) in continuous mode, in the 2–50° 2θ angular range, with a step size of 0.01^◦^ at a scan speed of 8° min^–1^.

#### 3.1.1 *N*-(2-(Diphenylphosphino)­phenyl)-methylene Benzylamine (**L** ^**1**^)

Benzylamine (49 μL, 0.447 mmol, 1.3 equiv) was added to a yellow solution of PCHO (100 mg, 0.344 mmol, 1.0 equiv) in 3/1 MeOH/DCM (9 mL/3 mL) mixture; the resulting solution was stirred under reflux overnight. The obtained colorless solution was allowed to cool to RT, then it was reduced to a small volume (ca. 1–2 mL) and left at −20 °C overnight. A white precipitate formed, which was filtered, washed with a small volume of cold MeOH and dried *in vacuo*. **L** ^**1**^ was isolated as a white solid (28 mg, yield = 21%). IR (cm^–1^): ν = 3052 (w) (CH sp^2^), 2829 (w) (CH sp^3^), 1636 (m) (C=N). ^1^H NMR (400 MHz, CDCl_3_, 298 K): δ­(ppm) = 9.04 (d, *J* = 5.1 Hz, 1H, CH=N), 8.07 (ddd, *J* = 7.8, 4.0, 1.4 Hz, 1H, ArH), 7.44–7.27 (m, 13H, ArH), 7.25–7.21 (m, 2H, ArH), 7.08–7.02 (m, 2H, ArH), 6.93–6.86 (m, 1H, ArH), 4.70 (s, 2H, CH_2_). ^1^H NMR (400 MHz, DMSO-*d*_6_, 298 K): δ­(ppm) = 8.93 (d, *J* = 4.8 Hz, 1H, CH=N), 7.95 (ddd, *J* = 7.6, 4.0, 1.5 Hz, 1H, ArH), 7.50–7.36 (m, 8H, ArH), 7.26–7.17 (m, 7H, ArH), 7.02–6.94 (m, 2H, ArH), 6.82 (ddd, *J* = 7.7, 4.5, 1.4 Hz, 1H, ArH), 4.62 (s, 2H, CH_2_). ^13^C NMR (101 MHz, CDCl_3_, 298 K): δ­(ppm) = 160.71 (d, *J* = 22.5 Hz), 139.50 (d, *J* = 17.3 Hz), 139.08, 137.77, 137.58, 136.45 (d, *J* = 9.5 Hz), 134.23 (d, *J* = 20.0 Hz), 133.35, 132.12, 130.53, 129.07, 129.04, 128.84, 128.77, 128.50, 128.15, 127.81 (d, *J* = 4.4 Hz), 126.93, 65.23. ^31^P NMR (162 MHz, CDCl_3_, 298 K): δ­(ppm) = −14.01 (s). ^31^P NMR (162 MHz, DMSO-*d*_6_, 298 K): δ­(ppm) = −14.49 (s). MS-ESI (positive ions; MeOH): *m*/*z* (%): 275 (100) [M–C_7_H_6_N]^+^, 380 (98) [M + H]^+^. Elem. anal. calcd (%) for C_26_H_22_NP: C 82.30%, H 5.84%, N 3.69%; found: C 82.06%, H 5.79%, N 3.41%.

#### 3.1.2 General Procedure for the Synthesis of 1:1 Iminophosphine:Copper­(I) Complexes

The reaction was performed under N_2_ atmosphere, and the solvents were degassed prior to use. To a solution of PCHO (100 mg, 0.344 mmol, 1.0 equiv) in CHCl_3_ (10 mL) was added a solution of CuI (65.9 mg, 0.346 mmol, 1.0 equiv) in MeCN (10 mL) and the resulting mixture was stirred at 50 °C for 1 h (after 15 min a yellow precipitate formed). The desired amine (0.344 mmol, 1.0 equiv) in MeCN (10 mL) was added and the resulting suspension was stirred at 60–65 °C for 5 h: the initial yellow suspension became an orange-red solution and then an orange precipitate formed. The mixture was allowed to cool to RT, then it was left at 4 °C under nitrogen overnight. The precipitate was filtrated, washed with cold MeCN and dried *in vacuo*.

##### 3.1.2.1 [Cu­(μ-I)­L^1^]_2_ **(1)**

Benzylamine (37.6 μL, 0.344 mmol) was used for the synthesis. The product was isolated as an orange-red powder (75 mg, yield = 38%). Mp 245–248 °C (dec.). IR (cm^–1^): ν = 3033 (w) (CH sp^2^), 2842 (w) (CH sp^3^), 1628 (m) (C=N). ^1^H NMR (400 MHz, CDCl_3_, 298 K): δ­(ppm) = 8.15 (s, br, 2H, CH=N), 7.59–7.26 (m, 24H, ArH), 7.25 (s, 2H, ArH), 7.22–6.85 (m, 12H, ArH), 5.02 (s, br, 4H, CH_2_). ^31^P NMR (162 MHz, CDCl_3_, 298 K): δ­(ppm) = −16.13 (s, br). MS-ESI (positive ions; MeCN): *m*/*z* (%): 442 [CuL^1^]^+^. Elem. anal. calcd (%) for C_52_H_44_Cu_2_I_2_N_2_P_2_: C 54.80%, H 3.89%, N 2.46%; found: C 54.76%, H 3.84%, N 2.42%. Single crystals suitable for XRD analysis were obtained by slow evaporation of a solution of the complex in CHCl_3_.

##### 3.1.2.2 [Cu­(μ-I)­L^2^]_2_ **(2)**

After the addition of 4-methylbenzylamine (43.8 μL, 0.344 mmol), temperature was raised to 60 °C. The product was isolated as an orange-red powder (99 mg, yield = 49%). Mp 190–192 °C (decomp.). IR (cm^–1^): ν = 3046 (w) (CH sp^2^), 2845 (w) (CH sp^3^), 1627 (m) (C=N). ^1^H NMR (400 MHz, CDCl_3_, 298 K): δ­(ppm) = 8.13 (s, br, 2H, CH=N), 7.60–7.27 (m, 24H, ArH), 7.25 (s, 2H, ArH), 6.99 (m, 10H, ArH), 4.98 (s, br, 4H, CH_2_), 2.28 (s, 6H, CH_3_). ^31^P NMR (162 MHz, CDCl_3_chloroform-*d*, 298 K): δ­(ppm) = −15.72 (s, br). MS-ESI (positive ions; MeCN): *m*/*z* (%): 456 (100) [CuL^2^]^+^, 850 (25) [CuL_2_^2^]^+^. Elem. anal. calcd (%) for C_54_H_48_Cu_2_I_2_N_2_P_2_: C 55.54%, H 4.14%, N 2.40%; found: C 55.65%, H 4.07%, N 2.34%. Single crystals suitable for XRD analysis were obtained by slow evaporation of mother liquors.

##### 3.1.2.3 [Cu­(μ-I)­L^3^]_2_ **(3)**

After the addition of the 4-fluorobenzylamine (39.3 μL, 0.344 mmol) amine, temperature was raised to 65 °C. The product was isolated as an orange-red powder (151 mg, yield = 71%). Mp 245–248 °C (dec.). IR (cm^–1^): ν = 3046 (w) (CH sp^2^), 2841 (w) (CH sp^3^), 1628 (m) (C=N). ^1^H NMR (400 MHz, CDCl_3_, 298 K): δ­(ppm) = 8.16 (s, br, 2H, CH=N), 7.59–7.26 (m, 26H, ArH), 7.06–6.98 (m, 6H, ArH), 6.78 (t, *J* = 8.5 Hz, 4H, ArH), 4.96 (s, br, 4H, CH_2_). ^31^P NMR (162 MHz, CDCl_3_, 298 K): δ­(ppm) = −15.77 (s, br). MS-ESI (positive ions; MeCN): *m*/*z* (%): 460 (100) [CuL^3^]^+^, 857 (40) [CuL_2_^3^]^+^. Elem. anal. calcd (%) for C_52_H_42_Cu_2_F_2_I_2_N_2_P_2_: C 53.12%, H 3.60%, N 2.38%; found: C 53.25%, H 3.62%, N 2.34%. Single crystals suitable for XRD analysis were obtained by slow evaporation of mother liquors.

#### 3.1.3 General Procedure for the Synthesis of 2:1 Iminophosphine:Copper­(I) Complexes Derived from CuI

The reaction was performed under N_2_ atmosphere and the solvents were degassed prior to use. To a solution of PCHO (100 mg, 0.344 mmol, 2.0 equiv) in CHCl_3_ (10 mL) was added a solution of CuI (32.8 mg, 0.172 mmol, 1.0 equiv) in MeCN (10 mL) and the resulting mixture was stirred at 50 °C for 1 h (after 15–20 min a yellow precipitate formed). The desired amine (0.344 mmol, 2.0 equiv) in MeCN (10 mL) was added and the resulting suspension was stirred at 50 °C for 5 h: the initial yellow suspension became a bright orange-red solution. The solution was allowed to cool to RT, then, depending on the amine, it was reduced to a small volume (3–4 mL) and an appropriate antisolvent was added, otherwise it was dried and recrystallized with the proper solvent mixture. The resulting precipitate was filtrated, washed with the appropriate solvent and dried *in vacuo*.

##### 3.1.3.1 Cu­(L^1^)_2_I **(4)**

Benzylamine (37.6 μL, 0.344 mmol) was used for the synthesis. Et_2_O was used as antisolvent. The product was isolated as a light orange solid (98 mg, yield = 59%). Mp 220–221 °C (dec.). IR (cm^–1^): ν = 3044 (w) (CH sp^2^), 2875 (w) (CH sp^3^), 1626 (m) (C=N). ^1^H NMR (400 MHz, CDCl_3_, 298 K): δ­(ppm) = 8.81 (s, br, 2H, CH=N), 8.05 (s, br, 2H, ArH), 7.66 (t, *J* = 7.6 Hz, 2H, ArH), 7.49–7.35 (m, 6H, ArH), 7.20–7.10 (m, 10H, ArH), 7.03–6.63 (m, 14H, ArH), 6.20 (s, br, 4H, ArH), 4.62 (s, br, 4H, CH_2_). ^1^H NMR (400 MHz, DMSO-*d*_6_, 298 K): δ­(ppm) = 8.75 (s, br, 2H, CH=N), 8.01 (d, *J* = 7.6 Hz, 2H, ArH), 7.84 (t, *J* = 7.6 Hz, 2H, ArH), 7.68 (t, *J* = 7.5 Hz, 2H, ArH), 7.48 (tt, *J* = 7.4, 1.2 Hz, 4H, ArH), 7.23 (m, 10H, ArH), 6.96 (t, *J* = 7.6 Hz, 4H, ArH), 6.87 (d, *J* = 7.6 Hz, 2H, ArH), 6.63 (s, br, 8H, ArH), 6.15 (s, br, 4H, ArH), 4.38 (s, br, 4H, CH_2_). ^13^C NMR (101 MHz, CDCl_3_, 298 K): δ­(ppm) = 168.56, 137.29, 136.50 (br), 136.26, 134.20 (br), 133.92, 131.74, 131.35 (br), 130.92, 130.29, 128.96, 128.59, 127.74, 127.30, 68.24. ^13^C NMR (126 MHz, DMSO-*d*_6_, 298 K): δ­(ppm) = 170.77, 137.48, 136.39, 133.64, 133.15 (br), 132.58, 131.21, 130.47, 129.04, 129.01, 128.97, 128.44, 127.60, 126.95, 67.69. ^31^P NMR (162 MHz, CDCl_3_, 298 K): δ­(ppm) = −8.62 (s, br). ^31^P NMR (162 MHz, DMSO-*d*_6_, 298 K): δ­(ppm) = −9.14 (s, br). MS-ESI (positive ions; MeCN): *m*/*z* (%): 442 (100) [CuL^1^]^+^, 822 (60) [CuL_2_^1^]^+^. Elem. anal. calcd (%) for C_52_H_44_CuIN_2_P_2_: C 65.79%, H 4.67%, N 2.95%; found: C 65.28%, H 4.93%, N 2.92%.

##### 3.1.3.2 Cu­(L^2^)_2_I·2H_2_O **(5)**

After addition of 4-methylbenzylamine (43.8 μL, 0.344 mmol), temperature was raised to 60 °C. After stirring for 5 h at 60 °C, the reaction mixture was dried and the solid recrystallized in CHCl_3_/*n*-hexane. The resulting suspension was left at RT overnight, then it was filtrated and washed with *n*-hexane. The obtained orange solid was triturated with Et_2_O and dried *in vacuo* (light orange solid, 56 mg, yield = 32%). Mp 210–213 °C (dec.). IR (cm^–1^): ν = 3049 (w) (CH sp^2^), 2834 (w) (CH sp^3^), 1626 (m) (C=N). ^1^H NMR (400 MHz, CDCl_3_, 298 K): δ­(ppm) = 8.69 (s, br, 2H, CH=N), 7.98 (s, br, 2H, ArH), 7.70–7.60 (m, 2H, ArH), 7.46–7.36 (m, 6H, ArH), 7.18 (t, *J* = 7.6 Hz, 8H, ArH), 7.01–6.59 (m, 14H, ArH), 6.14 (s, br, 4H, ArH), 4.63 (s, br, 4H, CH_2_), 2.28 (s, 6H, CH_3_). ^1^H NMR (400 MHz, DMSO-*d*_6_, 298 K): δ­(ppm) = 8.70 (s, 2H, CH=N), 7.99 (m, 2H, ArH), 7.85 (m, 2H, ArH), 7.70 (m, 2H, ArH), 7.53–7.45 (m, 4H, ArH), 7.23 (s, br, 8H, ArH), 6.98–6.44 (m, br, 14H, ArH), 5.98 (d, *J* = 7.7 Hz, 4H, ArH), 4.32 (s, br, 4H, CH_2_), 2.26 (s, 6H, CH_3_). ^13^C NMR (101 MHz, DMSO-*d*_*6*_, 298 K): δ­(ppm) = 170.22, 136.84, 135.45, 133.67, 133.34, 132.49, 131.10, 130.43, 128.95, 127.03, 64.91, 20.74. ^31^P NMR (162 MHz, DMSO-*d*_*6*_, 298 K): δ­(ppm)= −9.14 (s, br). MS-ESI (positive ions; MeCN): *m*/*z* (%): 456 (100) [CuL^2^]^+^, 850 (70) [CuL_2_^2^]^+^. Elem. anal. calcd (%) for C_54_H_52_CuIN_2_O_2_P_2_: C 64.00%, H 5.17%, N 2.76%; found: C 64.01%, H 4.97%, N 2.69%. Yellow crystals of **5a** suitable for XRD analysis were obtained by slow evaporation of mother liquors.

##### 3.1.3.3 Cu­(L^3^)_2_I·4H_2_O **(6)**

For the synthesis, the following molar ratios were used: 3:1:3 aldehyde:CuI:amine. A solution of CuI (32.8 mg, 0.172 mmol, 1.0 equiv) in MeCN (10 mL) was added to a solution of PCHO (150 mg, 0.516 mmol, 3.0 equiv) in CHCl_3_ (10 mL), and the resulting mixture was stirred at 50 °C for 1 h (after few minutes a yellow precipitate formed). 4-Fluorobenzylamine (59 μL, 0.516 mmol, 3.0 equiv) in MeCN (10 mL) was added and the resulting suspension was stirred at 65 °C for 5 h: the initial yellow suspension became a bright orange-red solution. The solution was allowed to cool to RT, then it was dried and recrystallized in CHCl_3_/*n*-hexane. A yellow precipitate formed and the suspension was left at RT for 2 h, then it was filtrated, washed with *n*-hexane and dried *in vacuo*. The product was isolated as a yellow solid (101 mg, yield = 55%). Mp 229–232 °C (dec.). IR (cm^–1^): ν = 3049 (w) (CH sp^2^), 2922 (w) (CH sp^3^), 1627, 1602 (m) (C=N). ^1^H NMR (500 MHz,CDCl_3_, 298 K): δ­(ppm) = 8.85 (s, br, 2H, CH=N), 8.03 (s, br, 2H, ArH), 7.63 (t, *J* = 7.2 Hz, 2H, ArH), 7.41 (t, *J* = 7.4 Hz, 6H, ArH), 7.21 (t, *J* = 7.7 Hz, 8H, ArH), 7.02–6.54 (m, 14H, ArH), 6.24 (s, br, 4H, ArH), 4.63 (s, br, 4H, CH_2_). ^1^H NMR (400 MHz, DMSO-*d*_*6*_, 298 K): δ­(ppm) = 8.76 (s, 2H, CH=N), 7.99 (d, *J* = 7.6 Hz, 2H, ArH), 7.83 (t, *J* = 7.6 Hz, 2H, ArH), 7.68 (t, *J* = 7.5 Hz, 2H, ArH), 7.55–7.47 (m, 4H, ArH), 7.26 (t, *J* = 7.6 Hz, 8H, ArH), 6.88 (d, *J* = 7.6 Hz, 2H, ArH), 6.83–6.51 (m, br, 12H, ArH), 6.23 (s, br, 4H, ArH), 4.39 (s, br, 4H, CH_2_). ^13^C NMR (101 MHz, DMSO-*d*_*6*_, 298 K): δ­(ppm) = 162.72, 160.30, 133.55, 133.20 (br), 132.59, 131.09, 130.55, 129.20, 129.11, 128.93, 115.25, 115.03, 79.17. ^31^P NMR (202 MHz, DMSO-*d*_*6*_, 298 K): δ­(ppm) = −9.11. MS-ESI (positive ions; MeCN): *m*/*z* (%): 460 (100) [CuL^3^]^+^, 858 (50) [CuL_2_^3^]^+^. Elem. anal. calcd (%) for C_52_H_50_CuF_2_IN_2_O_4_P_2_: C 59.07%, H 4.77%, N 2.65%; found: C 59.07%, H 4.23%, N 2.53%.

#### 3.1.4 General Procedure for the Synthesis of 2:1 Iminophosphine:Copper­(I) Complexes Starting from Cu­(NO_3_)_2_·3H_2_O

An excess of copper powder (ca. 0.2 mmol) was added to a light blue solution of Cu­(NO_3_)_2_ · 3H_2_O (20.8 mg, 0.086 mmol, 0.5 equiv) in MeCN (15 mL); the mixture was stirred under reflux until complete decolorization of the solution (ca. 30 min). The colorless solution was quickly hot filtered to remove unreacted copper powder, then it was degassed, put under N_2_ and added to a solution of PCHO (100 mg, 0.344 mmol, 2.0 equiv) in CHCl_3_ (10 mL). The resulting yellow solution was stirred at 50 °C under N_2_ for 1 h. The desired amine (0.344 mmol, 2.0 equiv) in MeCN (10 mL) was added and the mixture was stirred at 50 °C under N_2_ for 5 h: the initial yellow solution turned red-orange. The solution was allowed to cool to RT, then it was reduced to a small volume (3–4 mL) and Et_2_O (ca. 50 mL) was added. The resulting suspension was left at 4 °C under N_2_ overnight. The precipitate was filtrated, washed with Et_2_O and dried *in vacuo*.

##### 3.1.4.1 [Cu­(L^1^)_2_]­NO_3_ **(7)**

Benzylamine (37.6 μL, 0.344 mmol) was used for the synthesis. The product was isolated as a red powder (98 mg, yield = 64%). Mp 180–182 °C (dec.). IR (cm^–1^): ν = 3049 (w) (CH sp^2^), 2892 (w) (CH sp^3^), 1617 (m) (C=N), 1344 (s) (NO_2_ asym. str.). ^1^H NMR (400 MHz, CDCl_3_, 298 K): δ­(ppm) = 8.73 (s, 2H, CH=N), 8.12 (d, *J* = 7.5 Hz, 2H, ArH), 7.77 (t, *J* = 7.3 Hz, 2H, ArH), 7.45 (dt, *J* = 34.7, 7.5 Hz, 8H, ArH), 7.21–7.09 (m, 10H, ArH), 6.89–6.70 (m, 8H, ArH), 6.59 (s, br, 4H, ArH), 5.92 (d, *J* = 7.5 Hz, 4H, ArH), 4.52 (s, br, 4H, CH_2_). ^1^H NMR (400 MHz, DMSO-*d*_6_, 298 K): δ­(ppm) = 8.74 (s, 2H, CH=N), 8.06–7.98 (m, 2H, ArH), 7.86 (td, *J* = 7.5, 1.3 Hz, 2H, ArH), 7.69 (t, *J* = 7.6 Hz, 2H, ArH), 7.48 (t, *J* = 7.4 Hz, 4H, ArH), 7.33–7.16 (m, 10H, ArH), 6.95 (t, *J* = 7.6 Hz, 4H, ArH), 6.87 (dt, *J* = 7.8, 4.0 Hz, 2H, ArH), 6.60 (s, br, 8H, ArH), 6.13 (d, *J* = 7.5 Hz, 4H, ArH), 4.36 (s, 4H,CH_2_). ^1^H NMR (400 MHz, CD_3_OD, 298 K): δ­(ppm) = 8.63 (s, 2H, CH=N), 7.98 (dq, *J* = 7.8, 1.8 Hz, 2H, ArH), 7.83 (td, *J* = 7.6, 1.3 Hz, 2H, ArH), 7.69–7.59 (m, 2H, ArH), 7.47 (t, *J* = 7.5 Hz, 4H, ArH), 7.28–7.15 (m, 10H, ArH), 7.01–6.25 (m, br, 14H, ArH), 6.14 (dd, *J* = 8.2, 1.3 Hz, 4H, ArH), 4.45 (s, 4H, CH_2_). ^13^C NMR (101 MHz, CDCl_3_, 298 K): δ­(ppm) = 170.73, 138.16, 138.12, 138.09, 137.19, 135.79, 135.73, 135.66, 134.14 (br, ovlp) 133.99 (ovlp), 132.13, 132.11, 132.08, 131.39, 130.74, 130.62, 130.43, 129.09, 129.05, 129.00, 128.64, 127.36, 127.31, 69.11. ^13^C NMR (101 MHz, DMSO-*d*_*6*_, 298 K): δ­(ppm) = 170.76, 137.51, 137.48, 137.44, 136.38, 135.45, 135.38, 135.31, 133.64, 133.17 (br), 132.58, 131.20, 130.46, 129.97, 129.85, 129.73, 129.05, 129.00, 128.95, 128.44, 127.59, 126.94, 67.69. ^31^P NMR (162 MHz, CDCl_3_, 298 K): δ­(ppm) = −7.68 (s, br). ^31^P NMR (162 MHz, DMSO-*d*_6_, 298 K): δ­(ppm) = −9.07 (s). MS-ESI (positive ions; MeCN): *m*/*z* (%): 442 (100) [CuL^1^]^+^, 822 (10) [CuL_2_^1^]^+^. Elem. anal. calcd (%) for C_52_H_44_CuN_3_O_3_P_2_: C 70.62%, H 5.01%, N 4.75%; found: C 70.85%, H 4.97%, N 4.63%. Single crystals suitable for XRD analysis were obtained by slow evaporation of mother liquors.

##### 3.1.4.2 [Cu­(L^2^)_2_]­NO_3_·H_2_O **(8)**

4-Methylbenzylamine (43.8 μL, 0.344 mmol) was used for the synthesis. The product was isolated as an orange sticky solid (110 mg, yield = 69%). Mp 134–137 °C (dec.). IR (cm^–1^): ν = 3500–3300 (w) (OH), 3051 (w) (CH sp^2^), 2917 (w) (CH sp^3^), 1621 (m) (C=N), 1335 (s) (NO_2_ asym. str.). ^1^H NMR (500 MHz, CDCl_3_, 298 K): δ­(ppm) = 8.69 (s, 2H, CH=N), 8.09 (d, *J* = 6.6 Hz, 2H, ArH), 7.76 (td, *J* = 7.6, 1.0 Hz, 2H, ArH), 7.50 (t, *J* = 7.6 Hz, 2H, ArH), 7.42 (s, 4H, ArH), 7.15 (d, *J* = 6.4 Hz, 8H, ArH), 7.02–5.92 (m, 14H, ArH), 5.79 (d, *J* = 7.9 Hz, 4H, ArH), 4.46 (s, 4H, CH_2_), 2.28 (s, 6H, CH_3_). ^1^H NMR (400 MHz, DMSO-*d*_6_, 298 K): δ­(ppm) = 8.71 (s, 2H, CH=N), 8.04–7.97 (m, 2H, ArH), 7.85 (td, *J* = 7.6, 1.3 Hz, 2H, ArH), 7.70 (t, *J* = 7.6 Hz, 2H, ArH), 7.49 (t, *J* = 7.4 Hz, 4H, ArH), 7.22 (t, *J* = 7.6 Hz, 8H, ArH), 6.87 (dt, *J* = 8.0, 4.3 Hz, 2H, ArH), 6.81–6.29 (m, br, 12H, ArH), 5.98 (d, *J* = 7.8 Hz, 4H, ArH), 4.32 (s, 4H, CH_2_), 2.26 (s, 6H, CH_3_). ^13^C NMR (126 MHz, CDCl_3_, 298 K): δ­(ppm) = 170.30, 138.01, 137.98, 137.95, 136.90, 135.80, 135.75, 135.70, 134.14, 134.03, 132.06, 132.04, 131.35, 130.66, 130.56, 130.39, 129.29, 128.88, 127.34, 68.94, 21.36. ^13^C NMR (126 MHz, DMSO-*d*_6_, 298 K): δ­(ppm) = 170.30, 137.38, 136.86, 135.40, 133.68, 133.32, 133.12, 132.48, 131.11, 130.45, 128.96, 128.91, 128.87, 128.84, 127.01, 67.59, 20.75. ^31^P NMR (202 MHz, CDCl_3_, 298 K): δ­(ppm) = −7.85 (s, br). ^31^P NMR (162 MHz, DMSO-*d*_6_, 298 K): δ­(ppm) = −9.13 (s). MS-ESI (positive ions; MeCN): *m*/*z* (%): 456 (100) [CuL^2^]^+^, 850 (30) [CuL_2_^2^]^+^. Elem. anal. calcd (%) for C_54_H_50_CuN_3_O_4_P_2_: C 69.70%, H 5.42%, N 4.52%; found: C 70.24%, H 5.31%, N 4.63%.

##### 3.1.4.3 [Cu­(L^3^)_2_]­NO_3_·2H_2_O **(9)**

4-Fluorobenzylamine (39.3 μL, 0.344 mmol) was used for the synthesis. The product was isolated as an orange solid (113 mg, 69%). Mp 171–175 °C (dec.). IR (cm^–1^): ν = 3057 (w) (CH sp^2^), 2979 (w) and 2853 (w) (CH sp^3^), 1621 (m), 1604 (m) (C=N), 1364, 1323 (s) (NO_2_ asym. str.). ^1^H NMR (400 MHz, CDCl_3_, 298 K): δ­(ppm) = 8.78 (s, 2H, CH=N), 8.14 (dq, *J* = 7.7, 1.8 Hz, 2H, ArH), 7.76 (td, *J* = 7.5, 1.3 Hz, 2H, ArH), 7.47 (dt, *J* = 18.3, 7.5 Hz, 6H, ArH), 7.19 (t, *J* = 7.6 Hz, 8H, ArH), 6.91–6.18 (m, br, 14H, ArH), 5.90–5.78 (m, 4H, ArH), 4.50 (s, br, 4H, CH_2_).^1^H NMR (400 MHz, DMSO-*d*_6_, 298 K): δ­(ppm) = 8.76 (s, 2H, CH=N), 8.05–7.98 (m, 2H, ArH), 7.85 (td, *J* = 7.5, 1.2 Hz, 2H, ArH), 7.74–7.66 (m, 2H, ArH), 7.51 (t, *J* = 7.5 Hz, 4H, ArH), 7.26 (t, *J* = 7.6 Hz, 8H, ArH), 6.88 (dt, *J* = 7.9, 4.1 Hz, 2H, ArH), 6.79–6.55 (m, br, 12H, ArH), 6.17 (dd, *J* = 8.4, 5.4 Hz, 4H, ArH), 4.36 (s, 4H, CH_2_). ^13^C NMR (101 MHz, CDCl_3_, 298 K): δ­(ppm) = 171.01, 163.32, 160.88, 138.31, 138.27, 138.23, 135.76, 135.69, 135.62, 134.05 (br), 133.88, 133.31, 133.28, 132.14, 132.12, 132.09, 131.46, 130.63, 130.56, 130.44, 130.31, 129.05, 129.01, 128.97, 115.52, 115.30, 68.33. ^13^C NMR (101 MHz, DMSO-*d*_*6*_, 298 K): δ­(ppm) = 171.01, 162.77, 160.34, 137.53, 137.50, 137.46, 135.42, 135.35, 135.28, 133.62, 133.15 (br), 132.73, 132.58, 132.55, 131.20, 130.60, 129.72, 129.60, 129.48, 129.10, 129.02, 129.00, 128.95, 128.90, 115.28, 115.07, 66.98. ^31^P NMR (162 MHz, CDCl_3_, 298 K): δ­(ppm) = −7.54 (s, br). ^31^P NMR (162 MHz, DMSO-*d*_6_, 298 K): δ­(ppm) = −8.96 (s). MS-ESI (positive ions; MeCN): *m*/*z* (%): 460 (100) [CuL^3^]^+^, 858 (60) [CuL_2_^3^]^+^. Elem. anal. calcd (%) for C_52_H_46_CuF_2_N_3_O_5_P_2_: C 65.30%, H 4.85%, N 4.39%; found: C 65.54%, H 4.86%, N 4.20%.

#### 3.1.5 Synthesis of PCHO:Copper­(I) Complexes

##### 3.1.5.1 [Cu­(μ-I)­(MeCN)­L^a^]_2_

The reaction was performed under N_2_ atmosphere and the solvents were degassed prior to use. To a solution of PCHO (L^a^, 100 mg, 0.344 mmol, 1.0 equiv) in CHCl_3_ (10 mL) was added a solution of CuI (65.9 mg, 0.346 mmol, 1.0 equiv) in MeCN (10 mL) and the resulting mixture was stirred at 50 °C for 1 h. The obtained yellow suspension was allowed to cool to RT, then it was reduced to half volume. The suspension was left at 4 °C under N_2_ for 1 h, then it was filtrated, washed with cold MeCN and dried *in vacuo*. The product was isolated as a yellow powder (132 mg, yield = 74%). Mp 210–212 °C. IR (cm^–1^): ν = 2921 (w), 2829­(w), 2747 (w) (CH), 2295 (w), 2270 (w) (C≡N), 1688 (s) (C=O). ^1^H NMR (400 MHz, CDCl_3_, 298 K): δ­(ppm) = 10.09 (s, br, 2H, CH=O), 7.94 (d, *J* = 7.4 Hz, 2H, ArH), 7.62–7.27 (m, 24H, ArH), 7.00 (d, *J* = 7.6 Hz, 2H, ArH), 2.01 (s, 6H, CH_3_-CN). ^31^P NMR (162 MHz, CDCl_3_, 298 K): δ­(ppm) = −0.13 (s, br). MS-ESI (positive ions; MeCN): *m*/*z* (%): 353 (100) [Cu­(L^a^)]^+^, 643 (40) [Cu­(L^a^)_2_]^+^. Elem. anal. calcd (%) for C_42_H_36_Cu_2_I_2_N_2_O_2_P_2_: C 48.34%, H 3.48%, N 2.68%; found: C 47.88%, H 3.38%, N 2.16%.

##### 3.1.5.2 Cu­(L^a^)_2_I

The reaction was performed under N_2_ atmosphere and the solvents were degassed prior to use. To a solution of PCHO (100 mg, 0.344 mmol, 2.0 equiv) in CHCl_3_ (10 mL) was added a solution of CuI (32.8 mg, 0.172 mmol, 1.0 equiv) in MeCN (10 mL) and the resulting mixture was stirred at 50 °C for 1 h. The obtained yellow suspension was allowed to cool to RT, then it was reduced to half volume. The suspension was left at 4 °C under N_2_ for 2 h, then it was filtrated, washed with cold MeCN and dried *in vacuo*. The product was isolated as a yellow powder (100 mg, yield = 75%). Mp 254–256 °C. IR (cm^–1^): ν = 3061 (w), 2921 (w) (CH sp^2^), 2838 (w), 2744 (w) (aldehyde CH), 1696 (m), 1674 (s) (C=O). ^1^H NMR (400 MHz, CDCl_3_, 298 K): δ­(ppm) = 9.98 (s, br, 2H, CH=O), 7.89 (d, *J* = 7.7 Hz, 2H, ArH), 7.60 (td, *J* = 7.5, 1.2 Hz, 2H, ArH), 7.55–7.33 (m, 16H, ArH), 7.28 (s, 4H, ArH), 7.24 (s, 2H, ArH), 7.01 (d, *J* = 7.6 Hz, 2H, ArH). ^31^P NMR (162 MHz, CDCl_3_, 298 K): δ­(ppm) = −1.53 (s, br). MS-ESI (positive ions; MeCN): *m*/*z* (%): 290 (70) [L^a^]^+^, 353 (85) [Cu­(L^a^)]^+^, 643 (100) [Cu­(L^a^)_2_]^+^. Elem. anal. calcd (%) for C_38_H_30_CuIO_2_P_2_: C 59.19%, H 3.92%; found: C 59.62%, H 3.98%. Single crystals suitable for XRD analysis were obtained by slow evaporation of a solution of the complex in CHCl_3_.

##### 3.1.5.3 [Cu­(L^a^)_2_NO_3_]

To a light blue solution of Cu­(NO_3_)_2_·3H_2_O (20.8 mg, 0.086 mmol, 0.5 equiv) in MeCN (15 mL) was added an excess of copper powder (ca. 0.2 mmol) and the mixture was stirred under reflux until complete decolorization of the solution (ca. 30 min). The colorless solution was quick hot filtered to remove unreacted copper powder, then it was degassed, put under N_2_ and added to a solution of PCHO (100 mg, 0.344 mmol, 2.0 equiv) in CHCl_3_ (10 mL). The resulting yellow solution was stirred at 50 °C under N_2_ for 1 h. The solution was allowed to cool to RT, then it was reduced to a small volume (3–4 mL) and Et_2_O (ca. 50 mL) was added. The mixture was left at −20 °C under N_2_ overnight. The precipitate was filtrated, washed with Et_2_O and dried *in vacuo*. The product was isolated as a yellow crystalline solid (92 mg, yield = 76%). Mp 193–195 °C (dec.). IR (cm^–1^): ν = 3061 (w) (CH sp^2^), 2852 (w), 2757 (w) (CH aldehyde), 1692 (m), 1670 (s) (C=O), 1434 (s) (NO stretch), 1286 (s) (NO_2_ asym. stretch). ^1^H NMR (400 MHz, DMSO-*d*_6_, 298 K): δ­(ppm) = 9.97 (s, br, 2H, CH=O), 8.00 (s, br, 2H, ArH), 7.73 (dtd, *J* = 18.2, 7.4, 1.5 Hz, 4H, ArH), 7.56–6.96 (m, 20H, ArH), 6.85 (d, *J* = 7.5 Hz, 2H, ArH). MS-ESI (positive ions; MeCN): *m*/*z* (%): 353 (100) [Cu­(L^a^)]^+^, 643 (65) [Cu­(L^a^)_2_]^+^. Elem. anal. calcd (%) for C_38_H_30_CuNO_5_P_2_: C 64.63%, H 4.28%, N 1.98%; found: C 65.00%, H 4.35%, N 2.07%. Single crystals for X-ray diffraction analysis were selected from the sample.

### 3.2 Crystallography

Single crystal X-ray diffraction analysis were performed on selected single crystals on a Bruker D8 Venture diffractometer equipped with a kappa goniometer and a Photon II detector. Low-temperature data collections were performed under nitrogen flux employing an Oxford cryostream. Microfocused Mo–Kα radiation (λ=0.71073 Å) was used for **(3)** and **[Cu­(L** ^**a**^ **)**_**2**_ **NO**_**3**_ **]**, and Cu Kα radiation (λ = 1.54178 Å) for **(2)**, **(5a)**, and **(7)** for data acquisition. Lorentz polarization and absorption correction were applied. Data were reprocessed using APEX v3 software. Structures were solved by direct methods using SHELXT^29^ and refined by full-matrix least-squares on all F^2^ using SHELXL^30^ implemented in Olex2.15.^31^ Anisotropic displacement parameters were refined except for hydrogen atoms, which were introduced in calculated positions riding on their carrier atoms. The data deposited with the Cambridge Structural Database have the deposition numbers CCDC 2560043–2560047. The data can be obtained free of charge via www.ccdc.cam.ac.uk/data_request/cif or by emailing data_request@ccdc.cam.ac.uk, or by contacting The Cambridge Crystallographic Data center, 12 Union Road, Cambridge CB2 1EZ, UK. (http://www.ccdc.cam.ac.uk/data_request/cif).

### 3.3 Biological Studies

#### 3.3.1 Microbial Strains and Growth Conditions

Reference strains of *S. aureus* (ATCC 25923), *E. coli* (ATCC 25922) and *C. albicans* (ATCC 10231) were used in the study as microbial models. The strains were obtained from the American Type Culture Collection (ATCC, Manassas, VA, USA). Bacterial cultures were grown on 5% blood agar plates (Biolife Italiana S.r.l., Milan, Italy) at 37 °C while fungal cultures on Sabouraud Dextrose Agar (Biolife Italiana S.r.l., Milan, Italy) at 37 °C.

#### 3.3.2 Metal Complexes and Reference Drugs

The dry powder of the iminophosphine copper­(I) complexes was dissolved in 100% DMSO at 20 mM and then diluted with the appropriate cell culture medium to achieve the required concentrations. Ciprofloxacin, fluconazole, and cisplatin, purchased from Sigma-Aldrich (St. Louis, MO, USA), were used as reference drugs in the biological assays (Table S51).

#### 3.3.3 Determination of the MIC and IC_50_ Values

The antimicrobial activity of the compounds was evaluated *in vitro* by determining the Minimum Inhibitory Concentration (MIC) using a well-established broth microdilution method in compliance with the Clinical and Laboratory Standards Institute (CLSI) guidelines. Briefly, microbial inocula were adjusted to 0.5 McFarland (10^8^ CFU/mL) in phosphate-buffered saline (PBS); bacterial suspensions were subsequently diluted 1:200 in Mueller–Hinton broth (MH CondaLab, Laboratorios Conda S.A., Madrid, Spain), whereas fungal suspensions were diluted 1:20 in RPMI-1640 medium (Gibco, ThermoFisher Scientific Inc., Waltham, MA, USA), containing 1.8% glucose, 0.3% levo-glutamine buffered to pH 7.0 with MOPS at 0.165 M. Aliquots of 100 μL of the microbial suspensions were added to 96-well microplates containing 100 μL of the test compounds, 2-fold serially diluted to final concentrations ranging from 100 to 0.78 μM. Positive controls (microbial suspensions in growth medium), negative controls (medium only), and solvent controls were included in all experiments. Ciprofloxacin and fluconazole were used as reference antimicrobial agents. The plates were incubated at 37 °C for 24 h, and subsequently the optical density was measured at 630 nm.

MIC values were defined as the minimum concentrations required to inhibit 90% of microbial growth relative to the positive controls. For the most active compounds, IC_50_ values were also determined These values, corresponding to the concentration that reduce microbial growth by 50%, were calculated by nonlinear regression analysis. Specifically, dose–response curves were generated by plotting the percentage of growth inhibition, normalized to the positive control (100% growth) against the logarithm of the tested compounds concentrations. All statistical analyses and curve fittings were performed using GraphPad Prism software, version 9.4.1.^32^

#### 3.3.4 Cell Viability Assay

Vero (ATCC CCL-81) and Caco-2 (ATCC HTB-37) cell lines were used as experimental models to investigate the effects of the different metal complexes on nonmalignant mammalian cells and malignant human cells, respectively. Briefly, cells were cultured at 37 °C with 5% CO_2_ in Eagle’s Minimum Essential medium (EMEM, Lonza, Walkersville, MD, USA) supplemented with 10% fetal bovine serum (FBS; Microgen Laboratory Research srl), 100 μg/mL penicillin, and 100 μg/mL streptomycin. For the experiments, cells were seeded into 96-well plates at 10^4^ cells/well, and incubated at 37 °C for 24 h. The culture medium was then removed, and cell monolayers were incubated with 100 μL of fresh medium containing 2-fold serial dilutions of the compounds at the final concentrations ranging from 100 to 0.78 μM. Untreated cells were used as positive controls, whereas cells treated with 1% Triton X-100 served as negative controls. Cisplatin was included as reference compound.

Cell viability was determined using a well-established Neutral red uptake assay.^33^ After 48 h of treatment, the culture medium was removed, the cell monolayers were washed with PBS, and 100 μL of fresh medium containing 1:100 dilution of Neutral Red solution (4 mg/mL) was added. Following 3 h of incubation at 37 °C, the medium was discarded and cells were washed twice with PBS before adding 100 μL of destaining solution (50% ethanol, 49% water, and 1% glacial acetic acid) to each well. The plates were incubated under constant agitation (300 rpm) for 20–45 min, followed by the measurement of absorbance at 550 nm. Data were expressed as the percentage of cell viability relative to the untreated controls. The CC_50_ values were obtained from the corresponding dose–response curves, as previously reported for IC_50_ determination.

#### 3.3.5 Hemolytic Activity Assay

The hemolytic potential of the iminophosphine copper­(I) complexes was determined by quantifying the hemoglobin released upon the lysis of human red blood cells (hRBCs). Fresh hRBCs, obtained from anonymous donor, were isolated via centrifugation, washed with PBS, and adjusted to a final concentration of 4% (w/v) in the same buffer. For the assay, 100 μL of hRBCs suspension was mixed with an equal volume of the test compounds in a 96–well microplate, using 2-fold serial dilutions ranging from 100 to 0.78 μM. Since the solubilized samples exhibited intrinsic color, background controls consisting of the corresponding compound concentrations without hRBCs were included to assess potential interference with absorption measurements. Following the incubation at 37 °C for 1 h, the supernatants were transferring in a new 96-well plate and the released hemoglobin was the quantified at 405 nm. Untreated hRBCs in PBS were used as negative control (0% hemolysis), whereas hRBCs treated with 1% Triton X-100 served as the positive control (100% hemolysis). The percentage of hemolysis was calculated [OD_405nm_ (sample) – OD_405nm_ (background)]/[OD_405nm_ (positive control) – OD_405nm_ (negative control)] × 100. Finally, the Minimal Hemolytic Concentration (MHC) was defined as the lowest concentration of compound inducing 10% hemolysis.

#### 3.3.6 Plasmid DNA Interaction Assay

The interaction between the iminophosphine copper­(I) complexes and a plasmid DNA was investigated via agarose gel electrophoresis. Briefly, 200 ng of pmaxGFP plasmid (3527 bp; Lonza, Basel, Switzerland) were incubated with the test compounds (100 μM) in a Tris-HCl buffer (5 mM Tris-HCl, 50 mM NaCl, pH 7.20). Cisplatin (100 μM) was used as DNA-damage control, while the plasmid in the Tris-HCl buffer as negative control. After a 2 h incubation at 37 °C, all the reactions were stopped by adding 5 μL of loading buffer solution. The samples were subsequently analyzed using 1% agarose gel electrophoresis to assess any DNA modifications or binding events.

## 4 Conclusions

Despite the limited number of compounds investigated, some general considerations can be drawn about their biological activity. In **[CuL**_**2**_^**a**^ **(NO**_**3**_ **)]**, the ligand L^a^ is simply PCHO, that behaves as P-monodentate, and the aldehyde oxygen, with low affinity for the soft copper­(I) ion, does not coordinate. It is interesting to note that **[CuL**_**2**_^**a**^ **(NO**_**3**_ **)]** did not exhibit any detectable biological activity, including hemolytic effects on hRBCs, suggesting a lack of interaction with biological membranes as well as with plasmid DNA.

The other complexes (**4)**–(**9)** showed antibacterial activity against *S. aureus*; however, this effect was observed at concentrations that were also cytotoxic toward Vero cells, indicating poor selectivity and therefore limited potential as selective antibacterial agents.

Regarding their potential antiproliferative activity, the data obtained in the cellular assays indicate that iodide-containing complexes (**4)**–(**6)** display lower CC_50_ values in Caco-2 cells compared to Vero cells, suggesting a preferential cytotoxic effect toward malignant cells. In contrast, nitrate-containing complexes **(7)**–(**9)** exhibited the opposite trend. Taken together with the hemolysis results, which indicate limited membrane disruption at bioactive concentrations, these findings point to a possible intracellular mechanism of action such as their interaction with DNA molecules. Although at a qualitative level and under cell-free conditions, some complexes (**4)**–(**6)** induced changes in plasmid DNA topology consistent with a DNA strand-scission activity and in agreement with the antiproliferative property observed in Caco-2 cells. However, the mechanism underlying this effect cannot be established from the present experimental data. Further studies, including more detailed mechanistic investigations, are therefore required to elucidate the molecular pathways responsible for the observed DNA damage and to clarify its possible contribution to the antiproliferative activity of these complexes.

## Supplementary Material



## Data Availability

Data supporting the findings of this study are available within the article and its Supporting Information.

## Notes

Figure S1; (a) Comparison between the FT-IR spectra of **[Cu­(μ-I)­L** ^**1**^ **]**_**2**_ (blue) and **PCHO** (red); (b) comparison of the FT-IR spectrum of complex **[Cu­(μ-I)­L** ^**1**^ **]**_**2**_ (red) with ligand **L** ^**1**^ (blue); Figure S2: FT-IR spectrum of **(2)**; Figure S3: FT-IR spectrum of **(3)**; Figure S4: X-ray crystallographic data for compound **(2)**; Figure S5: ORTEP diagram of the crystal structure of complex **(3)**; Figure S6**:** (a) ^1^H NMR (400 MHz, 298 K); (b) ^31^P NMR (162 MHz, 298 K) spectra of **L** ^**1**^ (top) and of the complex **(1)** (bottom) in CDCl_3_; (c) ^31^P NMR (162 MHz, 298 K) spectrum of **(1)** immediately after preparation of the sample (top) and after 1 day (bottom) in DMSO-d_6_; Figure S7: ^1^H NMR (400 MHz, 298 K) and ^31^P NMR (162 MHz, 298 K) spectra of **(2**) in CDCl_3_; Figure S8: ^1^H NMR (400 MHz, CDCl_3_, 298 K) spectrum of **(3)**; Figure S9: ^31^P NMR (162 MHz, chloroform-*d*, 298 K) spectrum of **(3)**; Figure S10: ESI-MS spectrum (positive ions) for complex **(1)** in acetonitrile; Figure S11: ESI-MS spectrum (positive ions) for **(2)** in acetonitrile; Figure S12: ESI-MS spectrum (positive ions) for **(3)** in acetonitrile; Figure S13. (a) Synthesis of the complex **[Cu­(μ-I)­(MeCN)­L** ^**a**^ **]**_**2**_, **L** ^**a**^ = PCHO; Figure S14: ^1^H NMR (400 MHz, CDCl_3_, 298 K) spectrum of **[Cu­(μ-I)­(MeCN)­L** ^**a**^ **]**_**2**_; Figure S15: ^31^P NMR (162 MHz, CDCl_3_, 298 K) spectrum of **[Cu­(μ-I)­(MeCN)­L** ^**a**^ **]**_**2**_; Figure S16: ESI-MS spectrum (positive ions) for **[Cu­(μ-I)­(MeCN)­L** ^**a**^ **]**_**2**_ in acetonitrile; Figure S17: (a) calculated (in red) and experimental (in black) powder diffractogram of complex **[Cu­(μ-I)­(MeCN)­L** ^**a**^ **]**_**2**_; (b) ORTEP diagram of the crystal structure of **[Cu­(μ-I)­(MeCN)­L** ^**a**^ **]**_**2**_; Figure S18: FT-IR spectrum of **(4)**; Figure S19: FT-IR spectrum of **(5)**; Figure S20: FT-IR spectrum of **(6)**; Figure S21: (a) ^1^H NMR (400 MHz, 298 K) and (b) ^31^P NMR (162 MHz, 298 K) spectra of **L** ^**1**^ (top), complex **(1)** (middle), and complex **(4)** (bottom) in CDCl_3_; Figure S22: (top) ^1^H NMR (400 MHz, CDCl_3_, 298 K) spectrum of **(4)** immediately after preparation of the sample (purple), after 1 day (blue-green), after 2 days (green) and after 1 week (red). (bottom) ^31^P NMR (162 MHz, CDCl_3_, 298 K) spectrum of complex **(4)** immediately after preparation of the sample (purple), after 1 day (blue-green), after 2 days (green) and after 1 week (red); Figure S23: ^1^H NMR (400 MHz, DMSO-d_6_, 298 K) spectrum of **(4)**; Figure S24: ^1^H NMR (400 MHz, DMSO-d_6_, 298 K) spectrum of **(4)** immediately after preparation of the sample (top), after 1 day (middle) and after 2 days (bottom); Figure S25: ^1^H NMR (400 MHz, DMSO-d_6_, 298 K) spectrum of **(5)**; Figure S26: ^31^P NMR (162 MHz, DMSO-d_6_, 298 K) spectrum of **(5)**; Figure S27**:** ^1^H NMR (400 MHz, DMSO-d_6_, 298 K) spectrum of **(6)**; Figure S28: ^31^P NMR (202 MHz, DMSO-d_6_, 298 K) spectrum of **(6)**; Figure S29: ESI-MS spectrum (positive ions) for **(4)** in acetonitrile; Figure S30: ESI-MS spectrum (positive ions) for **(5)** in acetonitrile; Figure S31: ESI-MS spectrum (positive ions) for **(6)** in acetonitrile; Figure S32: X-ray crystallographic data and ORTEP representation for compound **(5a)**; Figure S33: FT-IR spectrum of [**Cu­(L** ^**a**^ **)**_**2**_ **I**]; Figure S34: (top) ^1^H NMR (400 MHz, 298 K) and (bottom) ^31^P NMR (162 MHz, 298 K) spectra of **[Cu­(L** ^**a**^ **)**_**2**_ **I]** in CDCl_3_; Figure S35: ESI-MS spectrum (positive ions) for [**Cu­(L** ^**a**^ **)**_**2**_ **I]** in acetonitrile; Figure S36: (top) FT-IR spectra of the complex **(7)** (in red) and **L** ^**1**^ (in blue); (bottom) FT-IR spectrum of **(7)**; Figure S37: FT-IR spectrum of **(8)**; Figure S38: FT-IR spectrum of **(9)**; Figure S39: (top) ^1^H NMR (400 MHz, CDCl_3_, 298 K) and (bottom) ^31^P NMR (162 MHz, CDCl_3_, 298 K) spectra of **(7)**; Figure S40: (a) ^1^H NMR (400 MHz, 298 K) spectrum of **(7)** recorded in DMSO-d_6_; (b) ^31^P NMR (162 MHz, 298 K) spectrum of **(7)** in DMSO-d_6_ measured immediately after preparation of the sample (purple), after 24 h (blue-green), 48 h (green) and 1 week (red); Figure S41: ^1^H NMR (400 MHz, DMSO-*d*_*6*_, 298 K) spectrum of **(8)**; Figure S42: ^31^P NMR (162 MHz, DMSO-*d*_*6*_, 298 K) spectrum of **(8)**; Figure S43: ^1^H NMR (400 MHz, DMSO-*d*_*6*_, 298 K) spectrum of **(9)**; Figure S44: ^31^P NMR (162 MHz, DMSO-*d*_*6*_, 298 K) spectrum of **(9)**; Figure S45: ESI-MS spectra (positive ions) for **(7)** (top), **(8)** and **(9)** (bottom) in acetonitrile; Figure S46: X-ray crystallographic data and ORTEP for compound **(7)**; Figure S47: FT-IR spectra of **[Cu­(L** ^**a**^ **)**_**2**_ **NO**_**3**_ **]** (in blue) and PCHO (in red); Figure S48: X-ray crystallographic data and ORTEP diagram for compound [**Cu­(L** ^**a**^ **)**_**2**_ **NO**_**3**_ **]**; Figure S49: ^1^H NMR (400 MHz, DMSO-*d*_*6*_, 298 K) spectrum of [**Cu­(L** ^**a**^ **)**_**2**_ **NO**_**3**_ **]**; Figure S50: ESI-MS spectrum (positive ions) for [**Cu­(L** ^**a**^ **)**_**2**_ **NO**_**3**_ **]** in acetonitrile; Table S51: IC_50_ and CC_50_ values (mean ± standard deviation) of the reference drug controls; Table S52: IC_50_ values (in μM) of copper­(I) iminophosphine complexes; Table S53: CC_50_ values (in μM) of copper­(I) iminophosphine complexes; Figure S54: Dose–response curves of the complexes obtained in Caco-2 cells; Table S55: CC_50_ values determined in Caco-2 cells and hemolytic activity (%) measured at the corresponding CC_50_ concentrations (PDF)
