# Supplementary material for: Synthesis and Biological Activity of Iminophosphine Copper(I) Complexes Derived from 2‑(Diphenylphosphino)benzaldehyde and 4‑Substituted-Benzylamines
Source: ACS Omega. 2026 Oct 28;11(44):68833–45. doi: 10.1021/acsomega.6c08107 (PMC13756700; doi:10.1021/acsomega.6c08107)
Supplement: Supplementary file 1 [file ao6c08107_si_006.pdf]

Supporting Information

Synthesis and biological activity of iminophosphine copper(I) complexes derived from 2-(diphenylphosphino)benzaldehyde and 4-substituted-benzylamines

Alessio Zavaroni[a], Luca Rigamonti[b], Giorgio Pelosi[a], Lorenzo Verderi[a], Matteo Incerti[c], Lisa Menegaldo[d], Diego Montagner[e], Dominga Rogolino[a], Francesca Bonvicini[d], Mauro Carcelli[a,*]

[a]*Department of Chemistry, Life Sciences, Environmental Sustainability, University of Parma, Parco Area delle Scienze 11/A, 43124 Parma, Italy*

[b]*Dipartimento di Scienze Chimiche e Geologiche, Università di Modena e Reggio Emilia, via G. Campi 103, 41125 Modena, Italy*

[c]*Food and Drug Department, University of Parma, Parco Area delle Scienze 27/A, 43124 Parma, Italy*

[d]*Department of Pharmacy and Biotechnology, University of Bologna, Via Massarenti 9, 40138 Bologna, Italy*

[e]*Department of Chemistry, Maynooth University, Maynooth, Ireland*

**Corresponding author*

Figure S1: (a) Comparison between the FT-IR spectra of **[Cu(μ-I)L¹]₂** (blue) and **PCHO** (red); (b) Comparison of the FT-IR spectrum of complex **[Cu(μ-I)L¹]₂** (red) with ligand **L¹** (blue), showing the slight shift of the C=N stretching band towards lower wavenumbers upon coordination.

Figure S2: FT-IR spectrum of **(2)**.

Figure S3: FT-IR spectrum of **(3)**.

Figure S4: X-ray crystallographic data for compound **(2).**

Figure S5: ORTEP diagram of the crystal structure of complex **(3)**, drawn with 50% probability ellipsoids (phosphorus atoms in light orange, nitrogen in blue, carbon in grey, hydrogens in white, iodine in purple, fluorine in yellow, copper in dark orange).

Figure S6: a) ^{1}H NMR (400 MHz, 298 K); b) ^{31}P NMR (162 MHz, 298 K) spectra of **L¹** (top) and of the complex **(1)** (bottom) in $CDCl_3$; c) ^{31}P NMR (162 MHz, 298 K) spectrum of **(1)** immediately after preparation of the sample (top) and after one day (bottom) in DMSO-d_6.

Figure S7: ^{1}H NMR (400 MHz*,* 298 K) and ^{31}P NMR (162 MHz, 298 K) spectra of (**2**) in $CDCl_3$.

Figure S8: ^{1}H NMR (400 MHz, $CDCl_3$*,* 298 K) spectrum of **(3)**.

Figure S9: ^{31}P NMR (162 MHz, chloroform-*d*, 298 K) spectrum of **(3)**.

Figure S10: ESI-MS spectrum (positive ions) for complex **(1)** in acetonitrile.

Figure S11: ESI-MS spectrum (positive ions) for **(2)** in acetonitrile.

Figure S12: ESI-MS spectrum (positive ions) for **(3)** in acetonitrile.

Figure S13. a) Synthesis of the complex **[Cu(μ-I)(MeCN)Lᵃ]₂**, **Lᵃ** = PCHO.

Figure S14: ^{1}H NMR (400 MHz, $CDCl_3$*,* 298 K) spectrum of **[Cu(μ-I)(MeCN)Lᵃ]₂**.

Figure S15: ^{31}P NMR (162 MHz, $CDCl_3$, 298 K) spectrum of **[Cu(μ-I)(MeCN)Lᵃ]₂**.

Figure S16: ESI-MS spectrum (positive ions) for **[Cu(μ-I)(MeCN)Lᵃ]₂** in acetonitrile.

Figure S17: (a) Calculated (in red) and experimental (in black) powder diffractogram of complex **[Cu(μ-I)(MeCN)Lᵃ]₂**; (b) ORTEP diagram of the crystal structure of **[Cu(μ-I)(MeCN)Lᵃ]₂**, drawn with 50% probability ellipsoids (phosphorus atoms in light orange, nitrogen in blue, carbon in grey, hydrogens in white, iodine in purple, oxygen in red, copper in dark orange; Aguirrechu-Comerón et al., 2018).

Figure S18: FT-IR spectrum of **(4)**.

Figure S19: FT-IR spectrum of **(5)**.

Figure S20: FT-IR spectrum of **(6)**.

Figure S21: (a) ^{1}H NMR (400 MHz, 298 K) and (b) ^{31}P NMR (162 MHz, 298 K) spectra of **L^{1}** (top), complex **(1)** (middle), and complex **(4)** (bottom) in $CDCl_3$.

Figure S22: (top) ^{1}H NMR (400 MHz, $CDCl_3$, 298 K) spectrum of **(4)** immediately after preparation of the sample (purple), after one day (blue-green), after two days (green) and after one week (red). (bottom) ^{31}P NMR (162 MHz, $CDCl_3$, 298 K) spectrum of complex **(4)** immediately after preparation of the sample (purple), after one day (blue-green), after two days (green) and after one week (red).

Figure S23: ^{1}H NMR (400 MHz, DMSO-d_6, 298 K) spectrum of **(4)**.

Figure S24: ^{1}H NMR (400 MHz, DMSO-d_6, 298 K) spectrum of **(4)** immediately after preparation of the sample (top), after one day (middle) and after two days (bottom).

Figure S25: ^{1}H NMR (400 MHz, DMSO-d_6, 298 K) spectrum of **(5)**.

Figure S26: ^{31}P NMR (162 MHz, DMSO-d_6, 298 K) spectrum of **(5)**.

Figure S27: ^{1}H NMR (400 MHz, DMSO-d_6, 298 K) spectrum of **(6)**.

Figure S28: ^{31}P NMR (202 MHz, DMSO-d_6, 298 K) spectrum of **(6)**.

Figure S29: ESI-MS spectrum (positive ions) for **(4)** in acetonitrile.

Figure S30: ESI-MS spectrum (positive ions) for **(5)** in acetonitrile.

Figure S31: ESI-MS spectrum (positive ions) for **(6)** in acetonitrile.

Figure S32: X-ray crystallographic data and ORTEP representation for compound **(5a)**

Figure S33: FT-IR spectrum of [**Cu(L^{a})$_2$I**].

Figure S34: (top) ^{1}H NMR (400 MHz, 298 K) and (bottom) ^{31}P NMR (162 MHz, 298 K) spectra of **[Cu(L^{a})$_2$I]** in $CDCl_3$

Figure S35: ESI-MS spectrum (positive ions) for [**Cu(L^{a})$_2$I]** in acetonitrile.

Figure S36: (top) FT-IR spectra of the complex **(7)** (in red) and **L^{1}** (in blue); (bottom) FT-IR spectrum of **(7)**.

Figure S37: FT-IR spectrum of **(8)**.

Figure S38: FT-IR spectrum of **(9)**.

Figure S39: (top) ^{1}H NMR (400 MHz, $CDCl_3$, 298 K) and (bottom) ^{31}P NMR (162 MHz, $CDCl_3$, 298 K) spectra of **(7)**.

Figure S40: a) ^{1}H NMR (400 MHz, 298 K) spectrum of **(7)** recorded in DMSO-d_6; b) ^{31}P NMR (162 MHz, 298 K) spectrum of **(7)** in DMSO-d_6 measured immediately after preparation of the sample (purple), after 24 hours (blue-green), 48 hours (green) and one week (red).

Figure S41: ^{1}H NMR (400 MHz, DMSO-d_6, 298 K) spectrum of **(8)**.

Figure S42: ^{31}P NMR (162 MHz, DMSO-d_6, 298 K) spectrum of **(8)**.

Figure S43: ^{1}H NMR (400 MHz, DMSO-d_6, 298 K) spectrum of **(9)**.

Figure S44: ^{31}P NMR (162 MHz, DMSO-d_6, 298 K) spectrum of **(9)**.

Figure S45: ESI-MS spectra (positive ions) for **(7)** (top), **(8)** and **(9)** (bottom) in acetonitrile.

Figure S46: X-ray crystallographic data and ORTEP for compound **(7).**

Figure S47: FT-IR spectra of **[$Cu(L^a)_2NO_3$]** (in blue) and PCHO (in red).

Figure S48: X-ray crystallographic data and ORTEP diagram for compound [**$Cu(L^a)_2NO_3$]**. (a) ORTEP diagram of the crystal structure of complex, drawn with 50% probability ellipsoids; (b) coordination geometry of copper(I) centre with indication of P-Cu-P and O-Cu-O angles (phosphorus atoms in light orange, nitrogen in blue, carbon in grey, hydrogens in white, oxygen in red, copper in dark orange).

Figure S49: ^{1}H NMR (400 MHz, DMSO-d_6, 298 K) spectrum of [**$Cu(L^a)_2NO_3$]**.

Figure S50: ESI-MS spectrum (positive ions) for [**$Cu(L^a)_2NO_3$]** in acetonitrile.

Table S51: IC_{50} and CC_{50} values (mean ± standard deviation) of the reference drug controls

Table S52: IC_{50} values (in μM) of copper(I) iminophosphine complexes as determined by the nonlinear regression (curve fit) obtained using GraphPad Prism version 10.0.0 for Windows, (GraphPad Software, Boston, Massachusetts USA, www.graphpad.com).

Table S53: CC_{50} values (in μM) of copper(I) iminophosphine complexes as determined by the nonlinear regression (curve fit) obtained using GraphPad Prism version 10.0.0 for Windows, (GraphPad Software, Boston, Massachusetts USA, www.graphpad.com).

Figure S54: Dose–response curves of the complexes obtained in Caco-2 cells generated by plotting the percentage of the cell viability relative to untreated controls and as a function of the logarithm of

the compound concentration (μM). CC_{50} values are indicated by the intersection with the 50% response level.

Table S55: CC_{50} values determined in Caco-2 cells and hemolytic activity (%) measured at the corresponding CC_{50} concentrations.

Figure S1: (a) Comparison between the FT-IR spectra of **[Cu(μ-I)L¹]₂** (blue) and **PCHO** (red); (b) Comparison of the FT-IR spectrum of complex **[Cu(μ-I)L¹]₂** (red) with ligand **L¹** (blue), showing the slight shift of the C=N stretching band towards lower wavenumbers upon coordination.

(a)

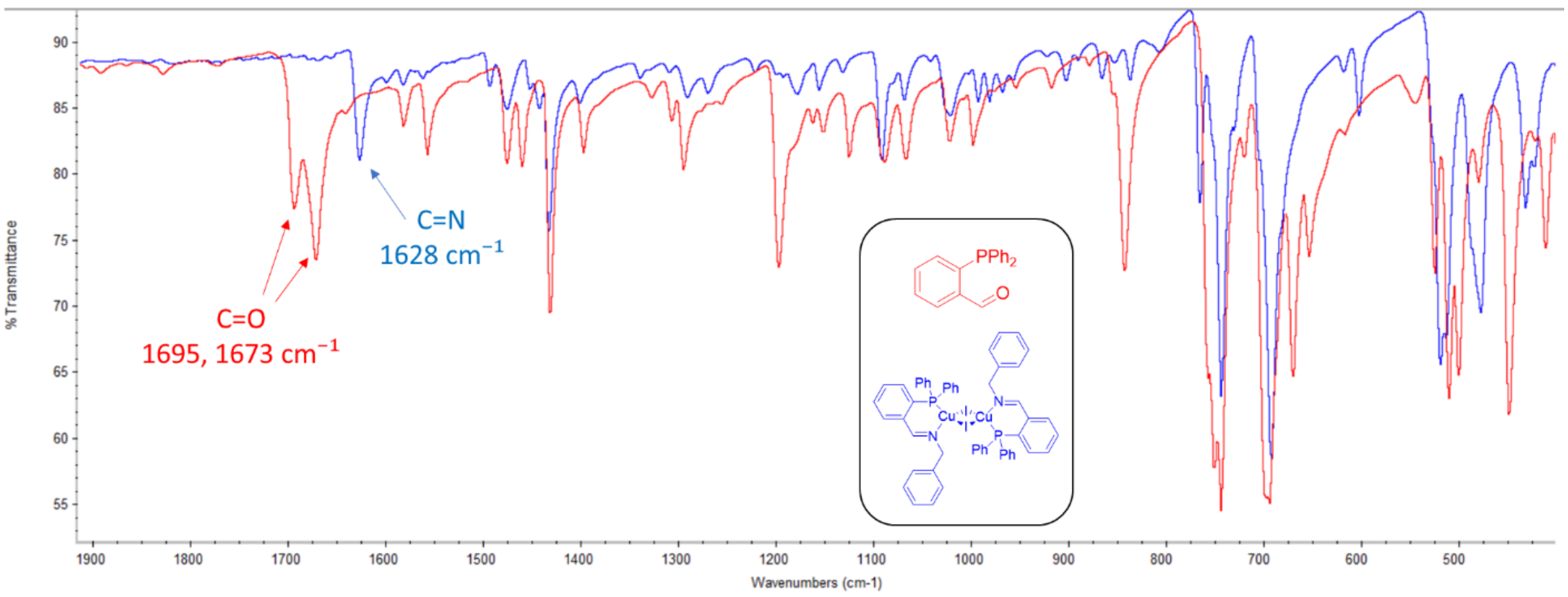

(b)

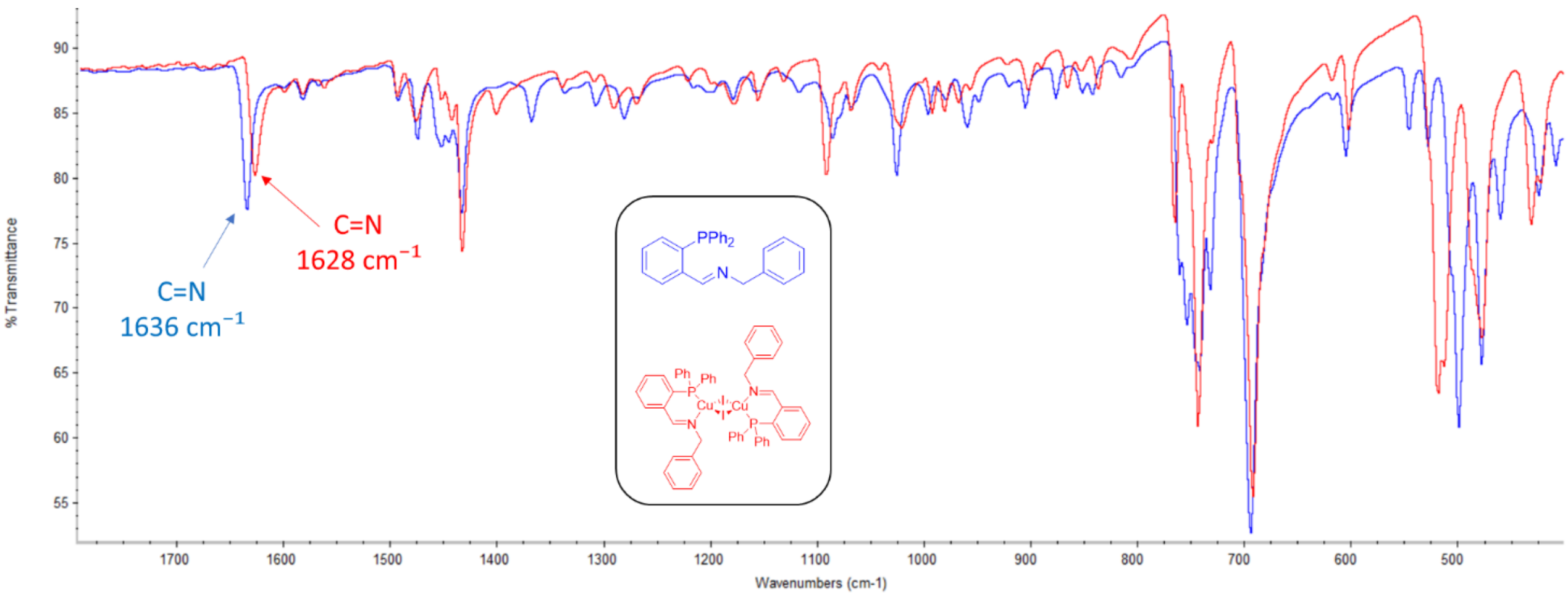

Figure S2: FT-IR spectrum of **(2)**.

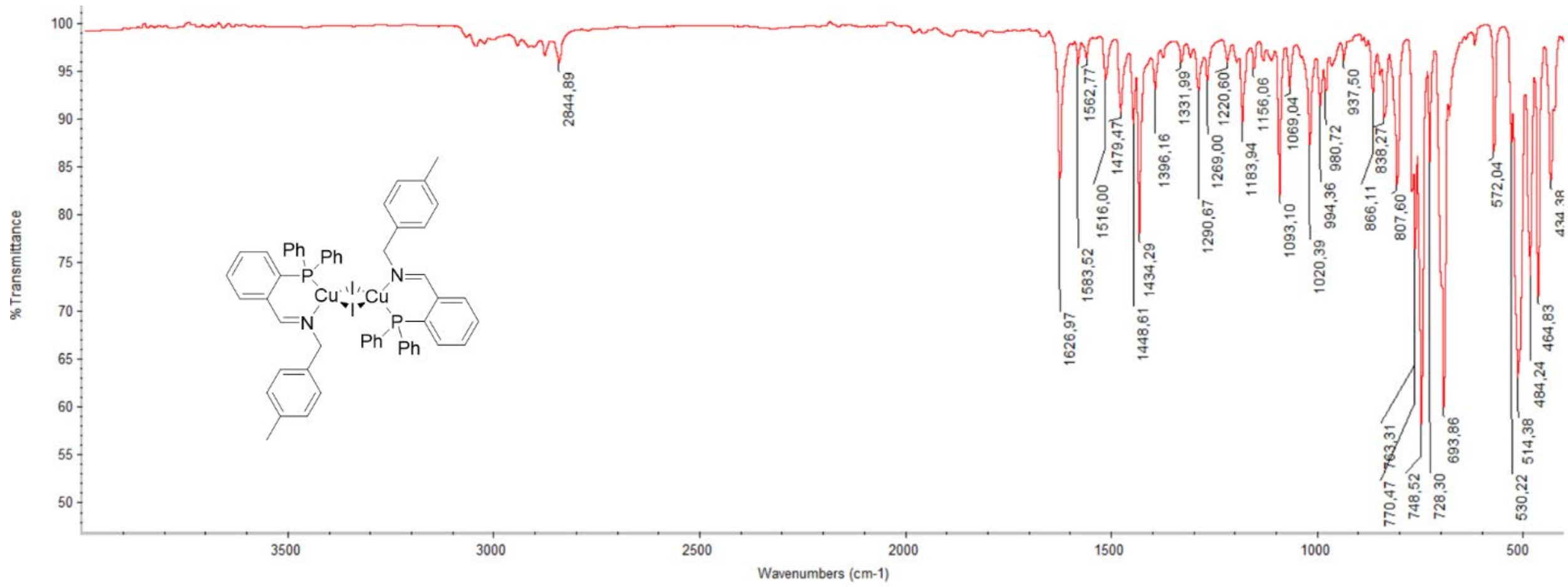

Figure S3: FT-IR spectrum of **(3)**.

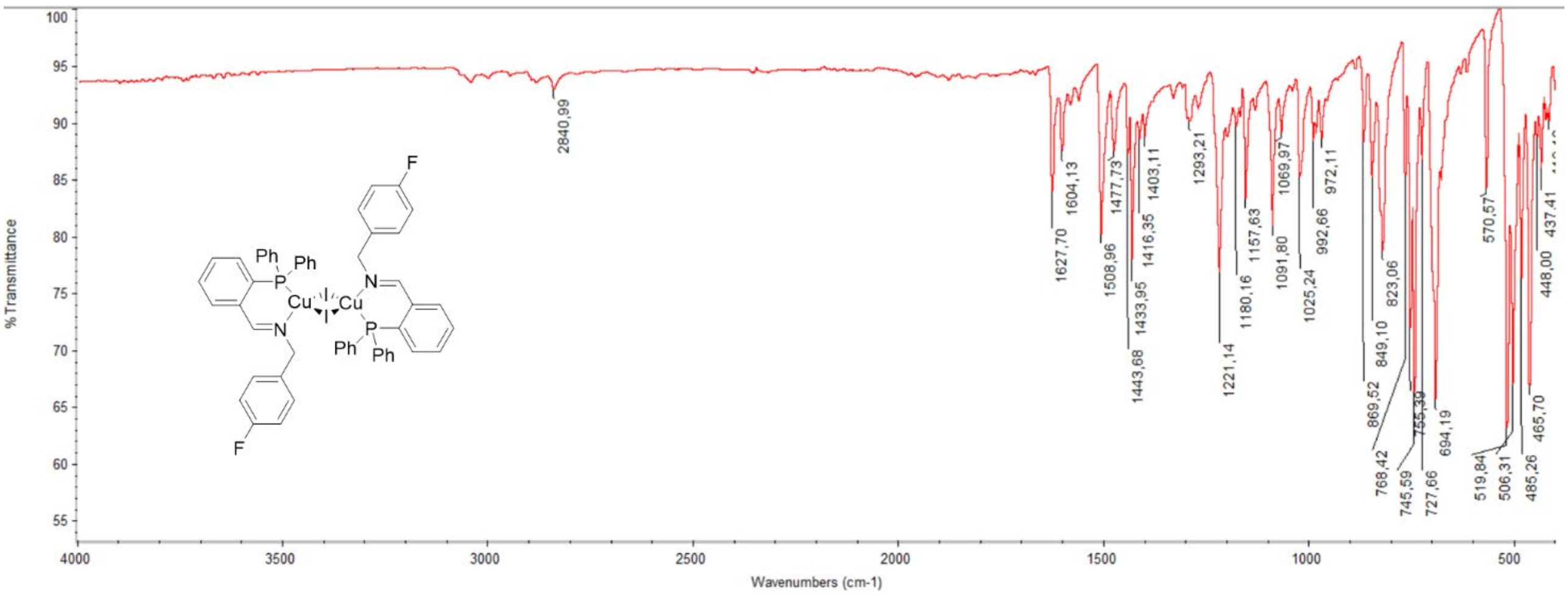

Figure S4: X-ray crystallographic data for compound **(2)**

**Empirical formula**	C54 H48 Cu2 I2 N2 P2
**Formula weight**	1167.76
**Temperature/K**	200.0
**Diffractometer/detector**	Bruker D8 Venture / PhotonII area detector
**Radiation**	CuKα (λ = 1.54178)
**Crystal system**	monoclinic
**Space group**	P21/c
**a/Å**	20.6138(8)
**b/Å**	9.3915(3)
**c/Å**	24.4735(11)
**α/°**	90
**β/°**	91.456(2)
**γ/°**	90
**Volume/Å^3**	4736.4(3)
**Z**	4
**ρcalc /g·cm^{-3}**	1.638
**μ/mm^{-1}**	12.274
**F(000)**	2320
**Θ range for data collection/°**	2.14 - 70.14
	-25<h<25
**Index ranges**	-11<k<11
	-29<l<29
**Reflections collected**	50656
**Unique reflections**	8963
**Parameters**	561
**Goodness-of-fit on F^2**	1.043
**Final R indexes [I>=2σ (I)]**	R=0.037, wR2=0.083
**CCDC Number**	2560046

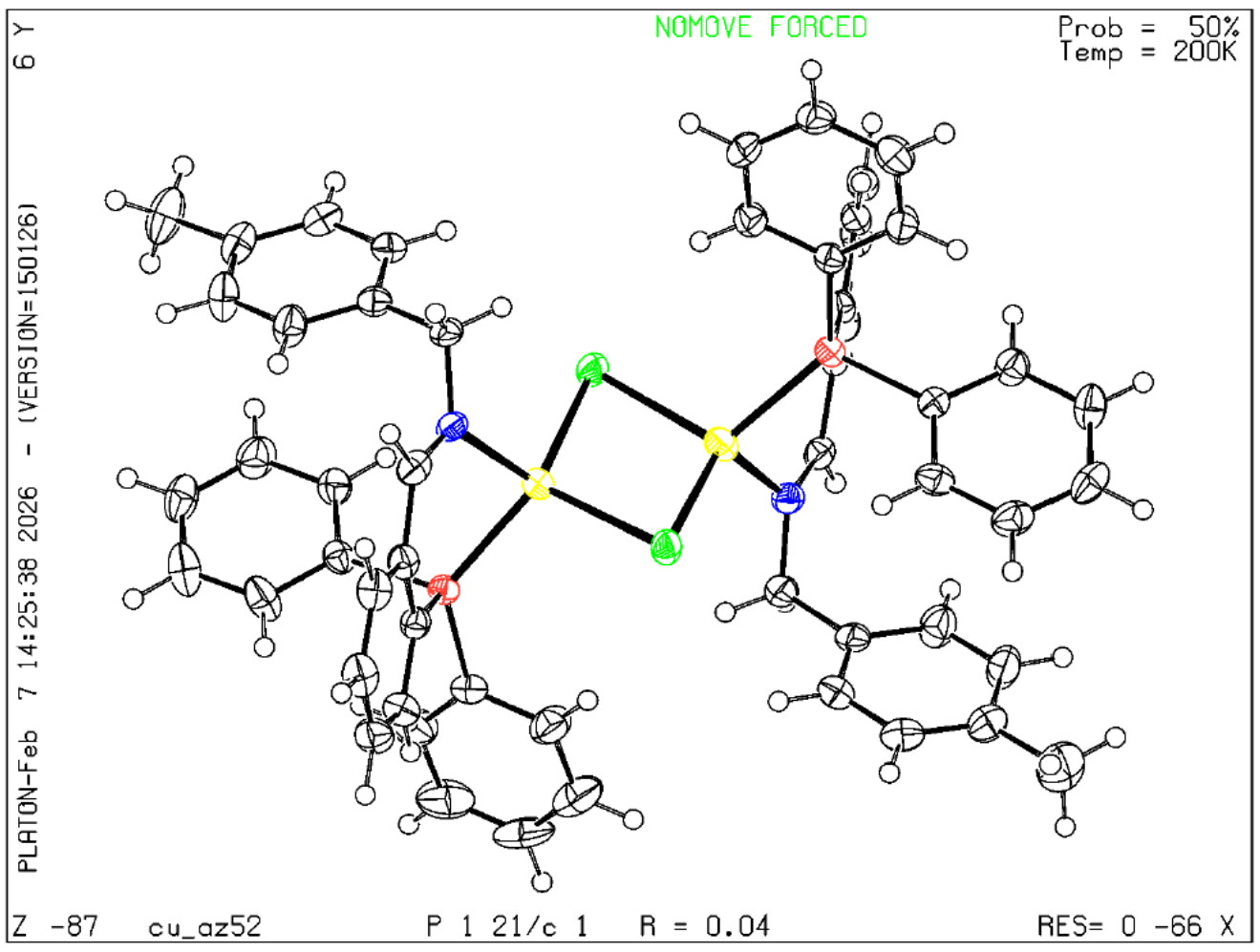

Figure S5: ORTEP diagram of the crystal structure of complex **(3)**, drawn with 50% probability ellipsoids (phosphorus atoms in light orange, nitrogen in blue, carbon in grey, hydrogens in white, iodine in purple, fluorine in yellow, copper in dark orange).

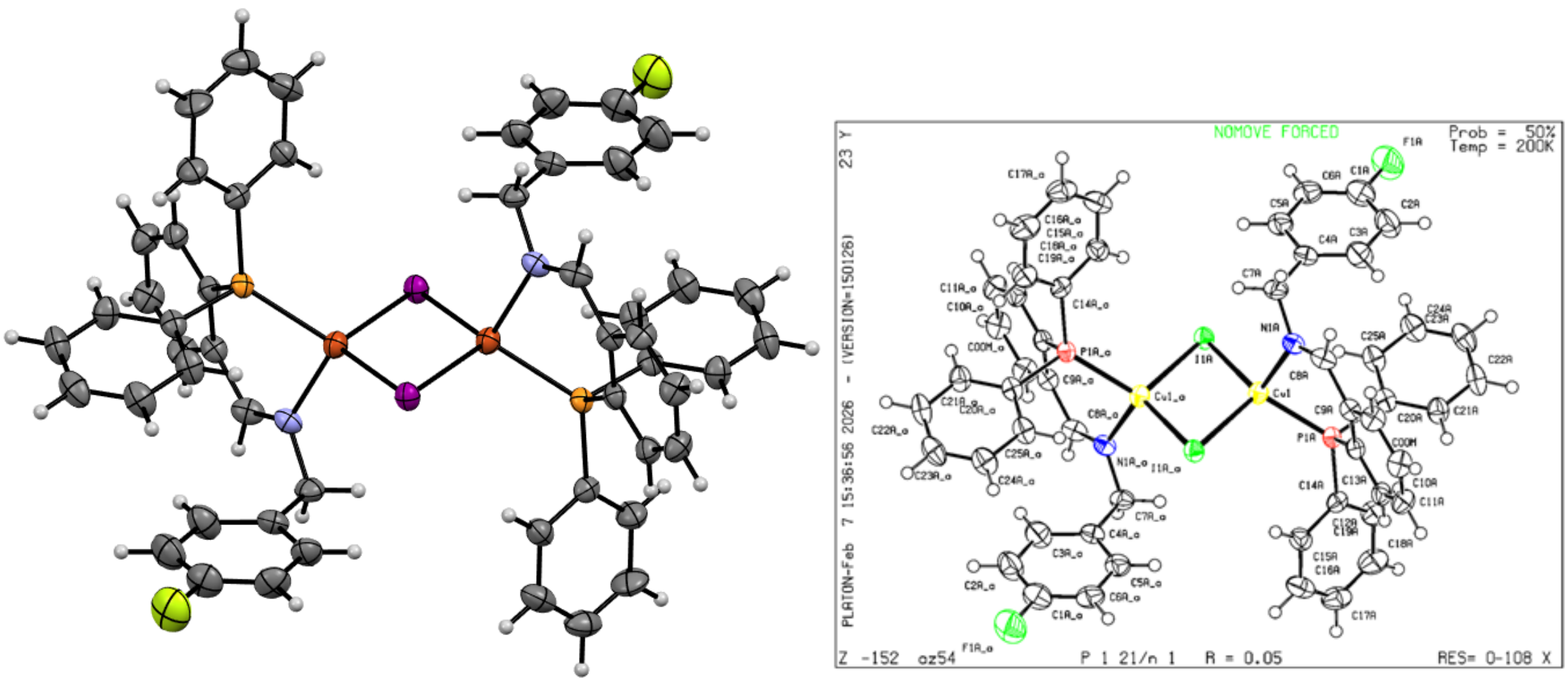

X-ray crystallographic data for compound **(3):**

**Empirical formula**	C26 H21 Cu F I N P
**Formula weight**	587.85
**Temperature/K**	200.0
**Diffractometer/detector**	Bruker D8 Venture / PhotonII area detector
**Radiation**	MoKα (λ = 0.71073)
**Crystal system**	monoclinic
**Space group**	P21/n
**a/Å**	15.954(3)
**b/Å**	9.0371(14)
**c/Å**	16.040(3)
**α/°**	90
**β/°**	95.709(6)
**γ/°**	90
**Volume/Å^3**	2301.2(7)
**Z**	4
**ρcalc /g·cm^{-3}**	1.697
**μ/mm^{-1}**	2.382
**F(000)**	1160
**Θ range for data collection/°**	1.897 - 25.027
	-18<h<18
**Index ranges**	-9<k<10
	-19<l<19
**Reflections collected**	15946
**Unique reflections**	3977
**Parameters**	280

**Goodness-of-fit on F^2**	1.272
**Final R indexes [I>=2σ (I)]**	R=0.052, wR2=0.146
**CCDC Number**	2560044

Figure S6: a) ^{1}H NMR (400 MHz, 298 K); b) ^{31}P NMR (162 MHz, 298 K) spectra of **L¹** (top) and of the complex **(1)** (bottom) in $CDCl_3$; c) ^{31}P NMR (162 MHz, 298 K) spectrum of **(1)** immediately after preparation of the sample (top) and after one day (bottom) in DMSO-d$_6$.

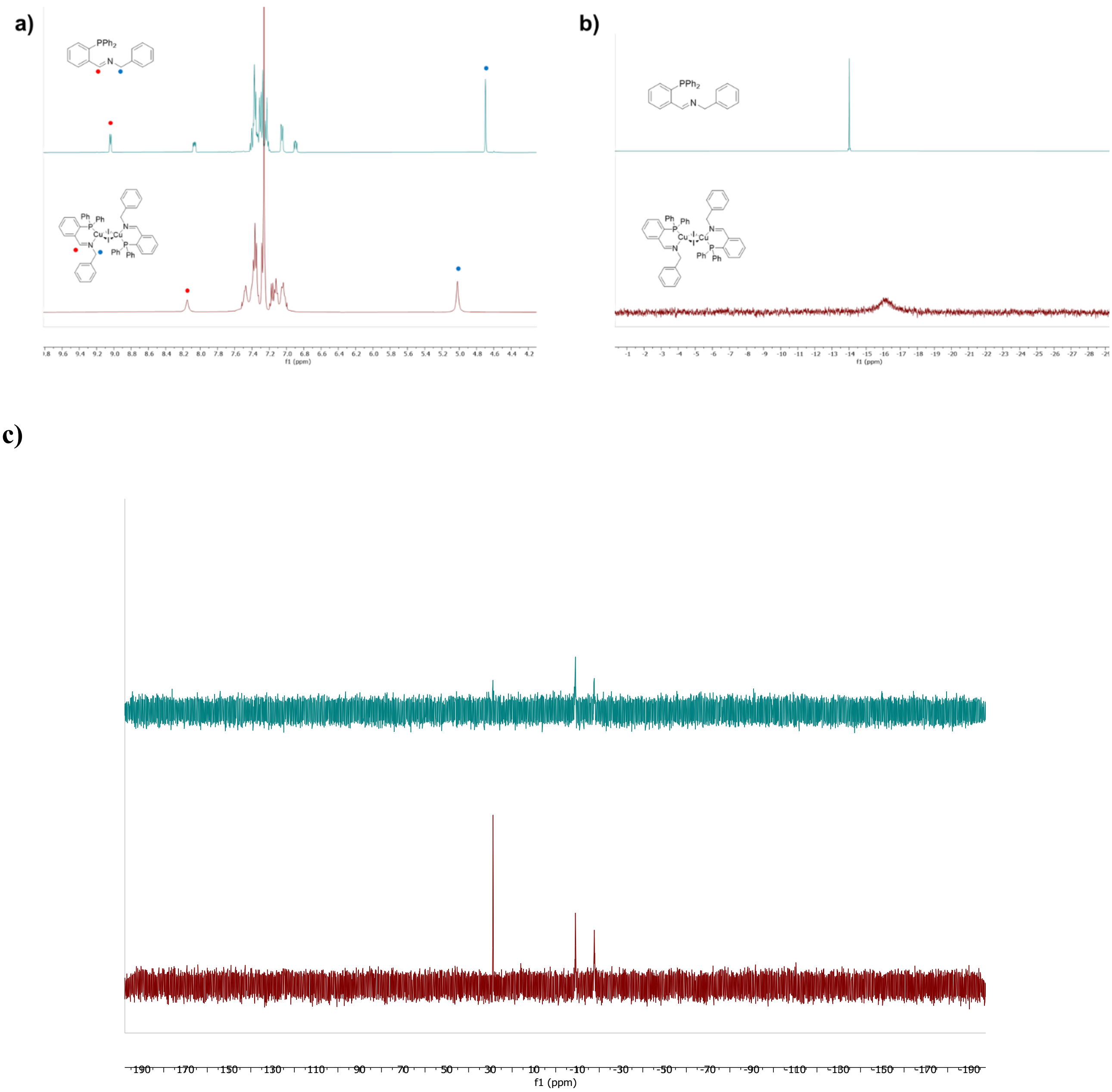

Figure S7: ^{1}H NMR (400 MHz, 298 K) and ^{31}P NMR (162 MHz, 298 K) spectra of **(2)** in $CDCl_3$.

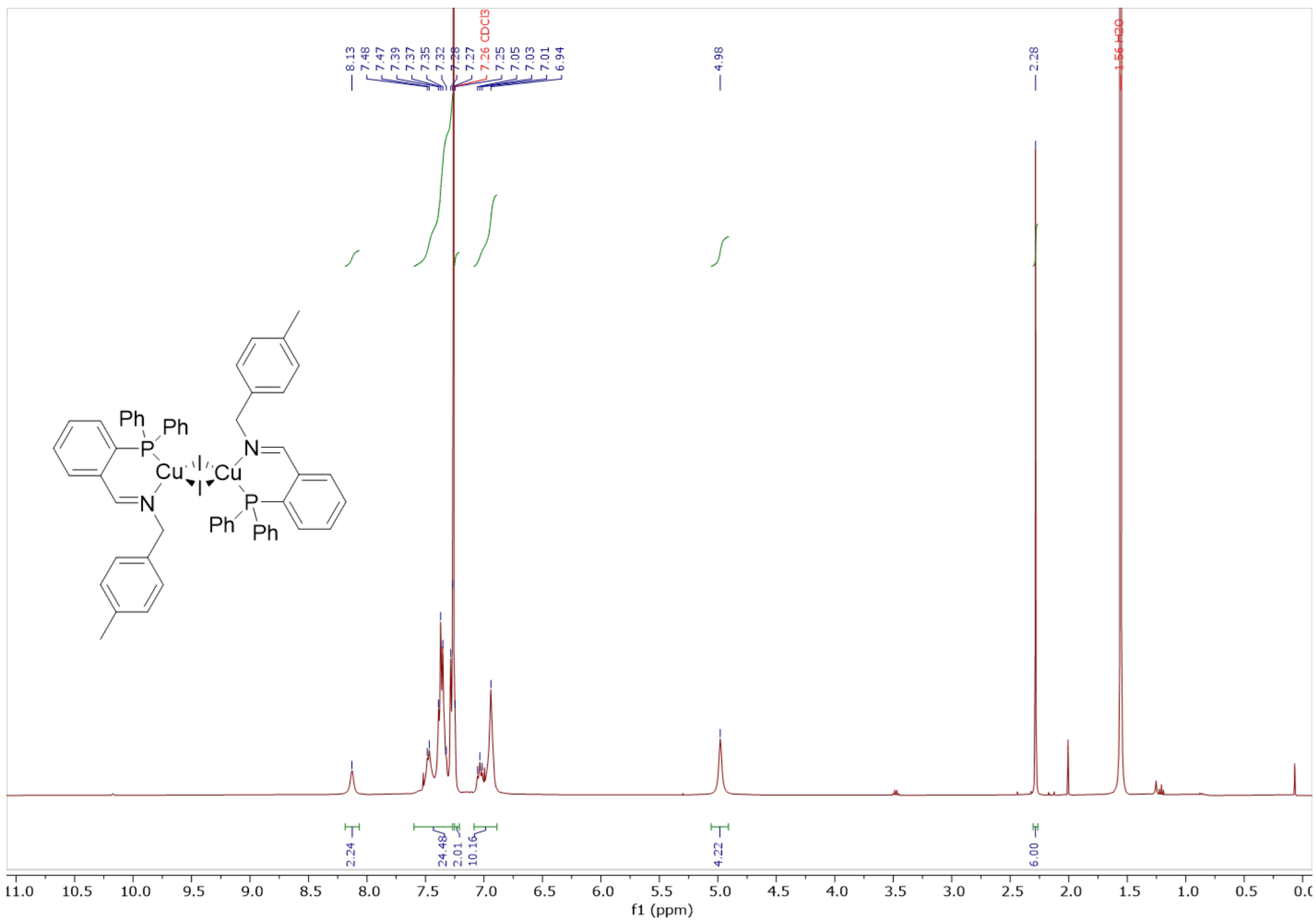

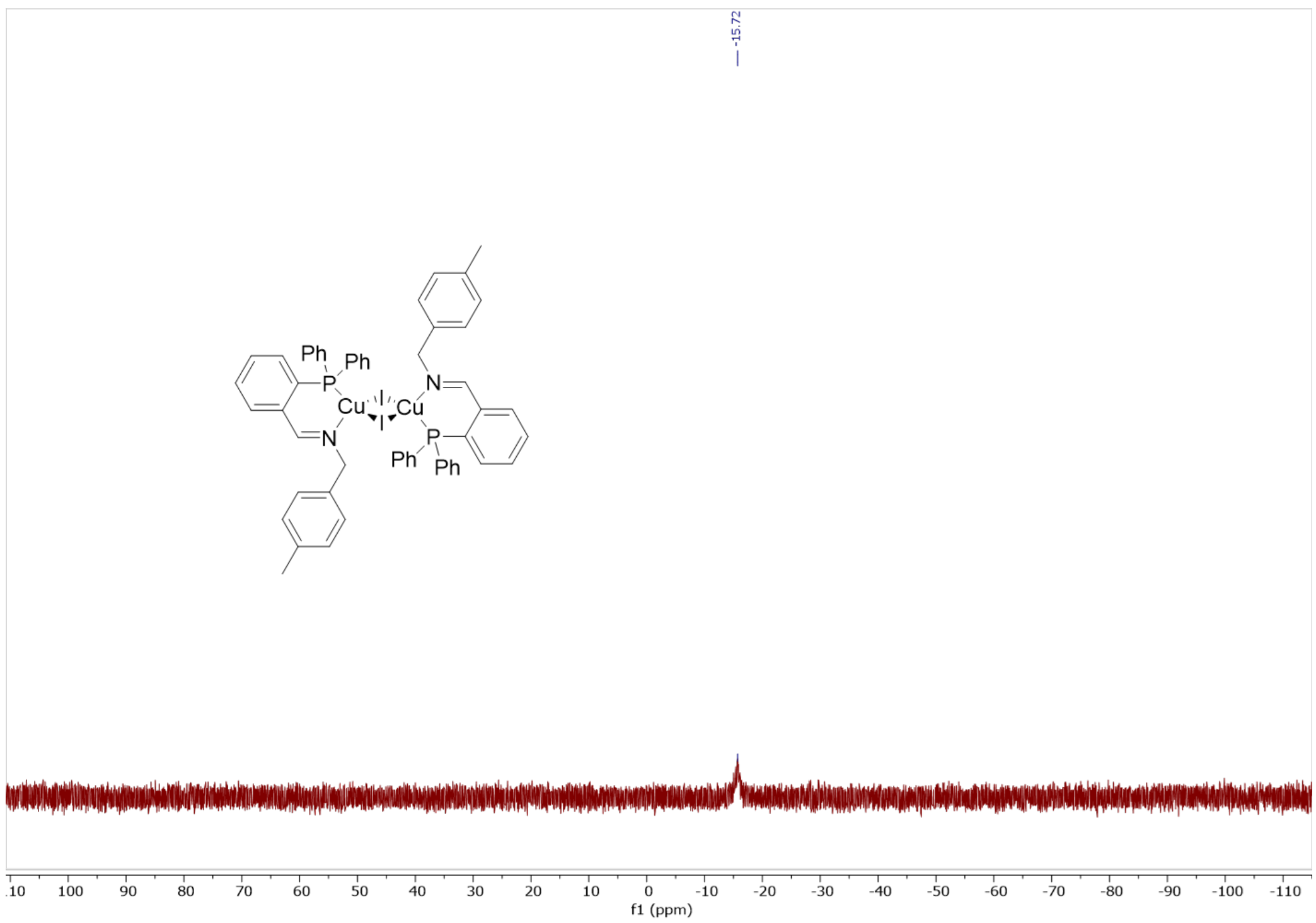

Figure S8: ^{1}H NMR (400 MHz, $CDCl_3$, 298 K) spectrum of **(3)**.

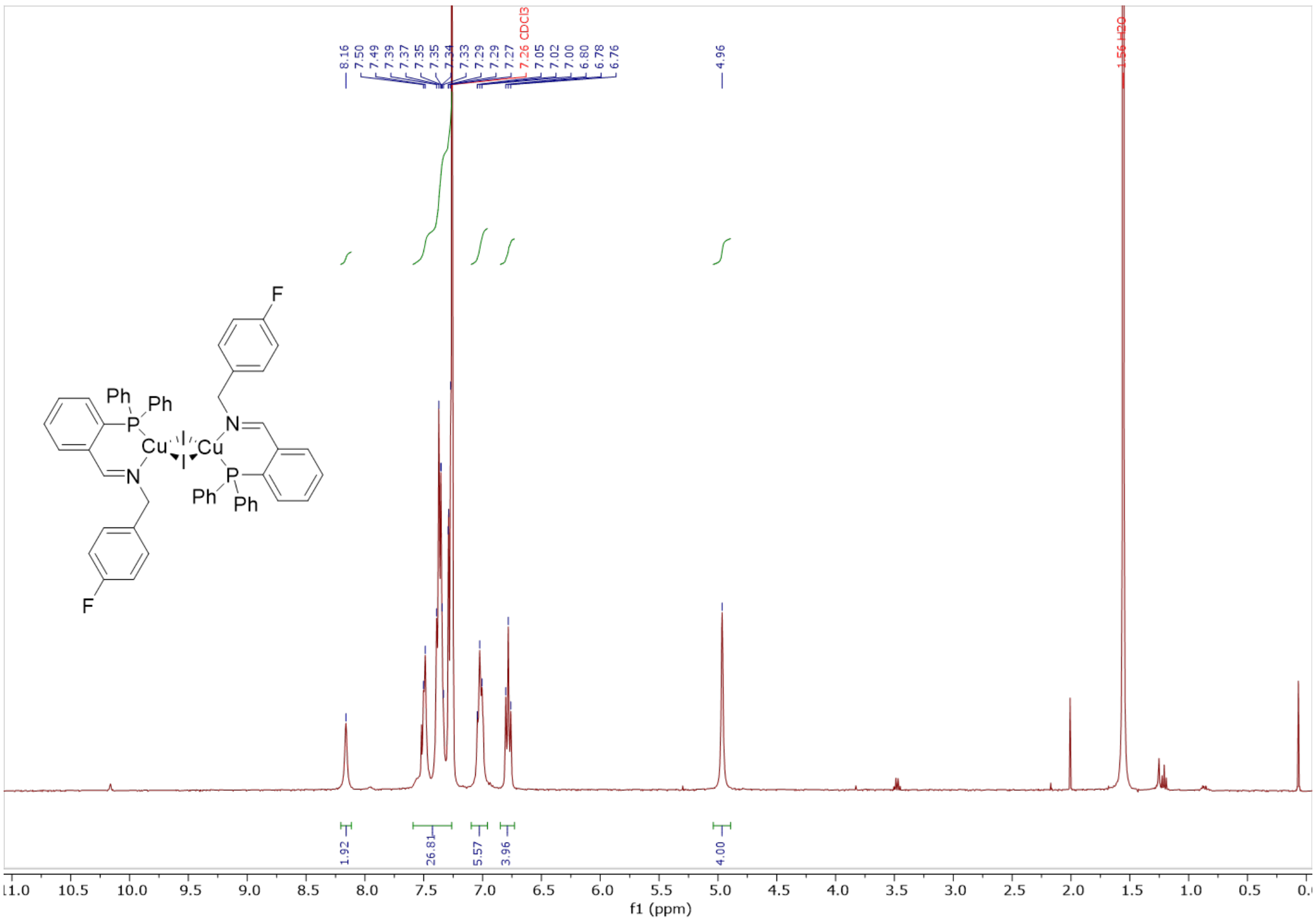

Figure S9: ^{31}P NMR (162 MHz, chloroform-*d*, 298 K) spectrum of **(3)**.

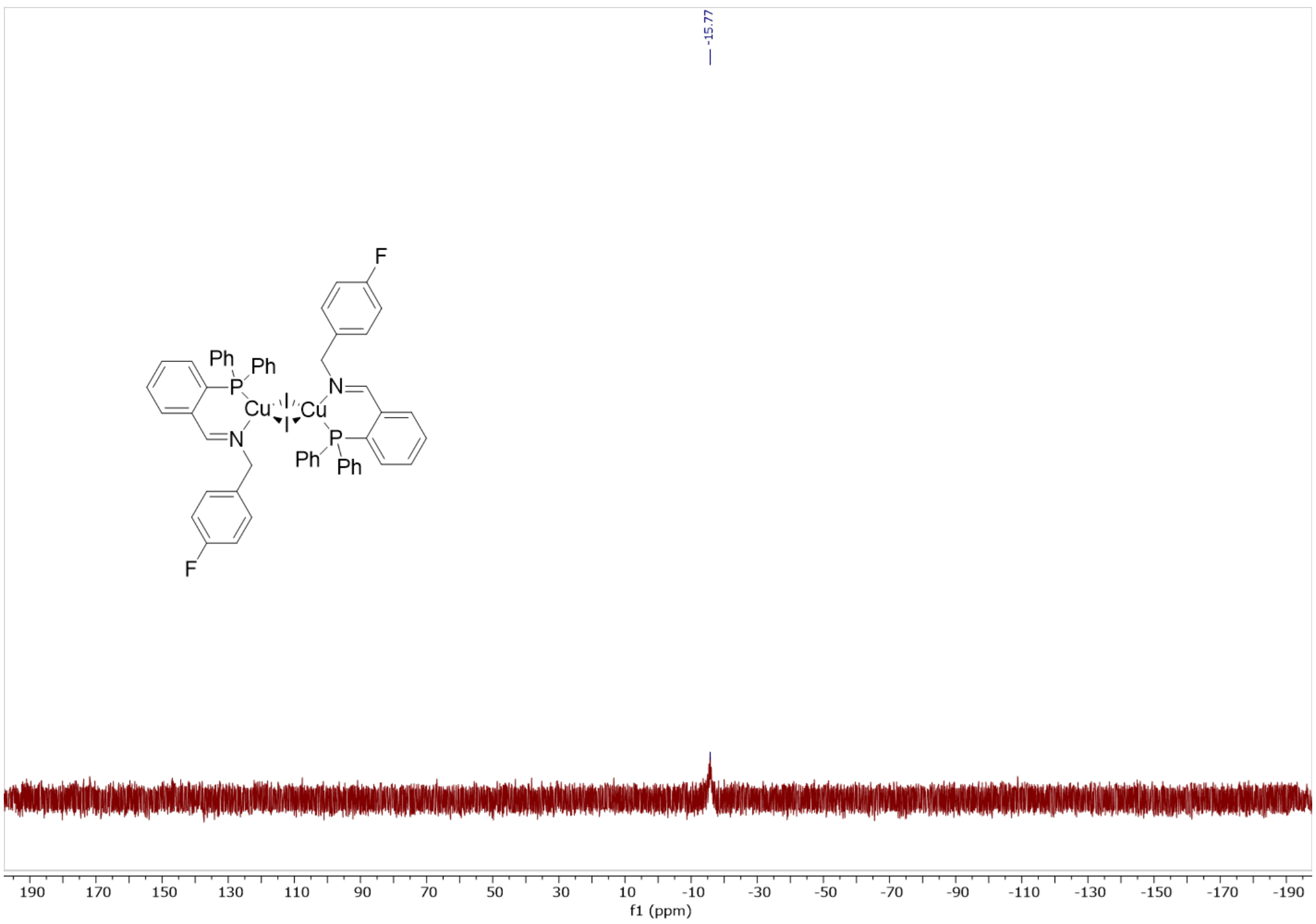

Figure S10: ESI-MS spectrum (positive ions) for complex **(1)** in acetonitrile.

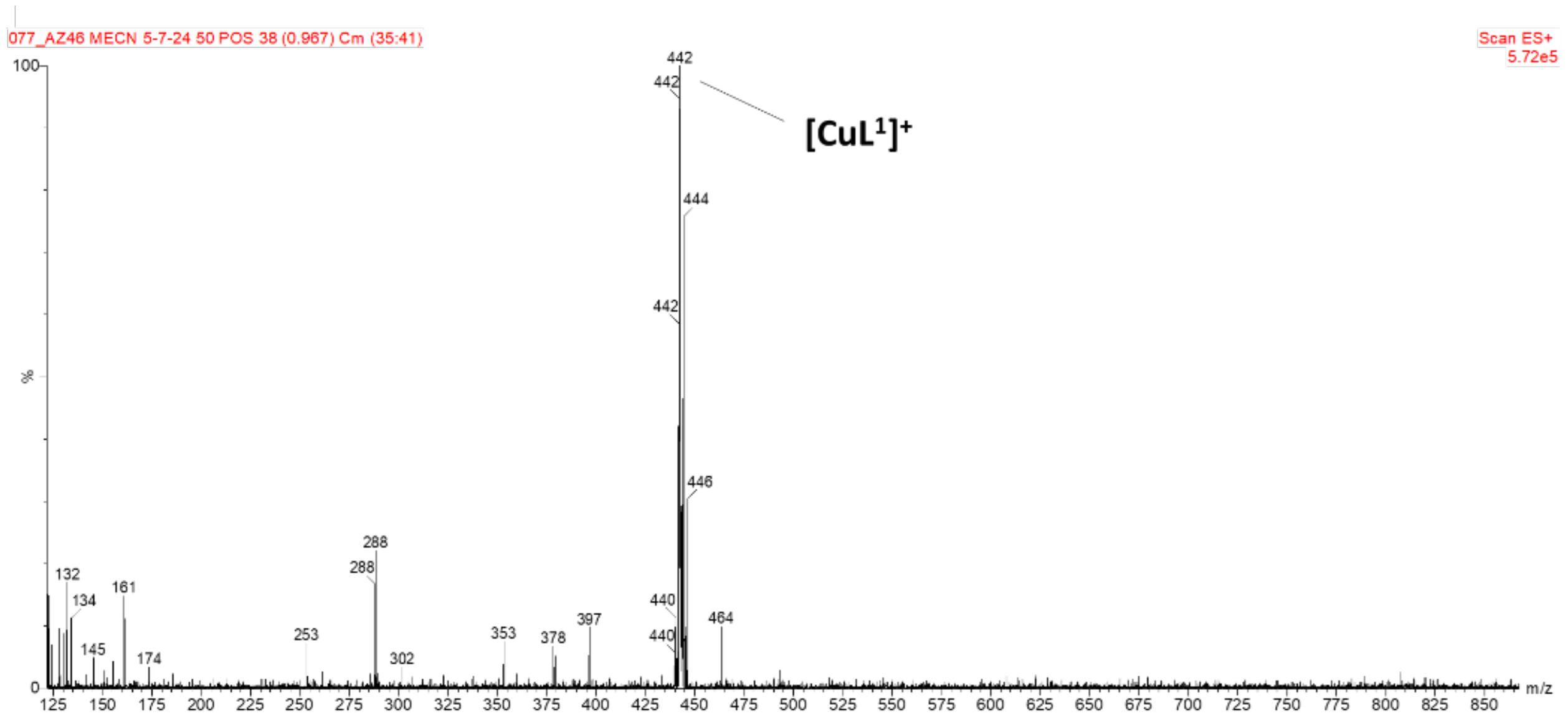

Figure S11: ESI-MS spectrum (positive ions) for **(2)** in acetonitrile.

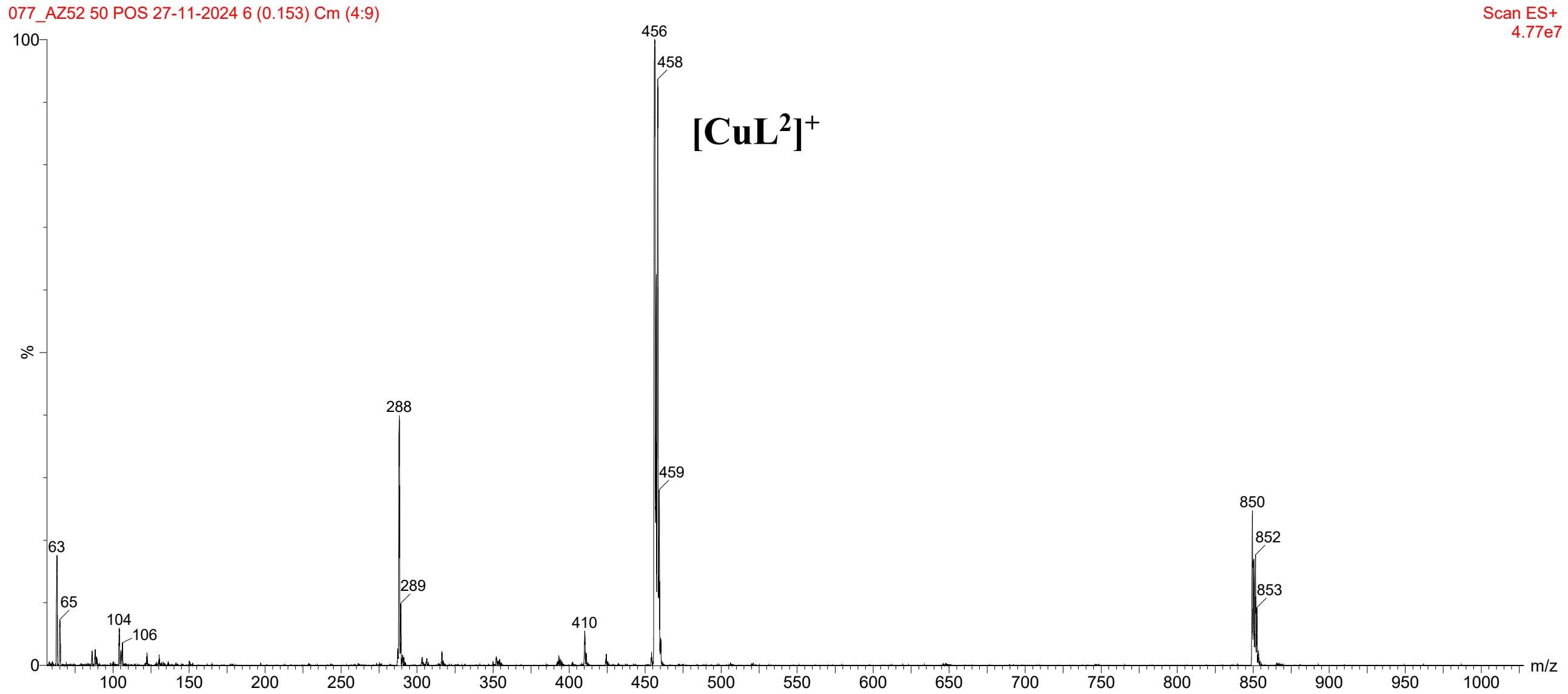

Figure S12: ESI-MS spectrum (positive ions) for **(3)** in acetonitrile.

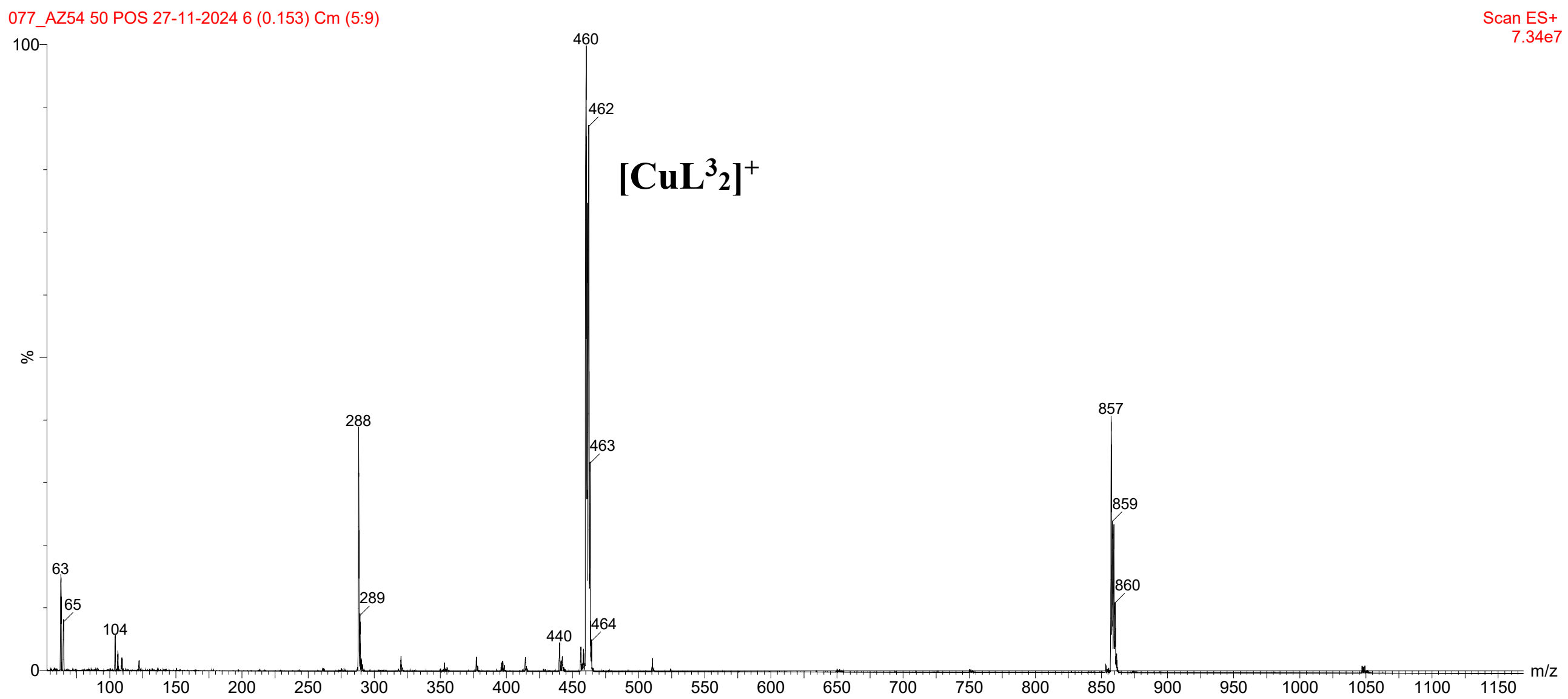

Figure S13. a) Synthesis of the complex **[Cu(μ-I)(MeCN)L[a]]$_2$**, **L[a]** = PCHO.

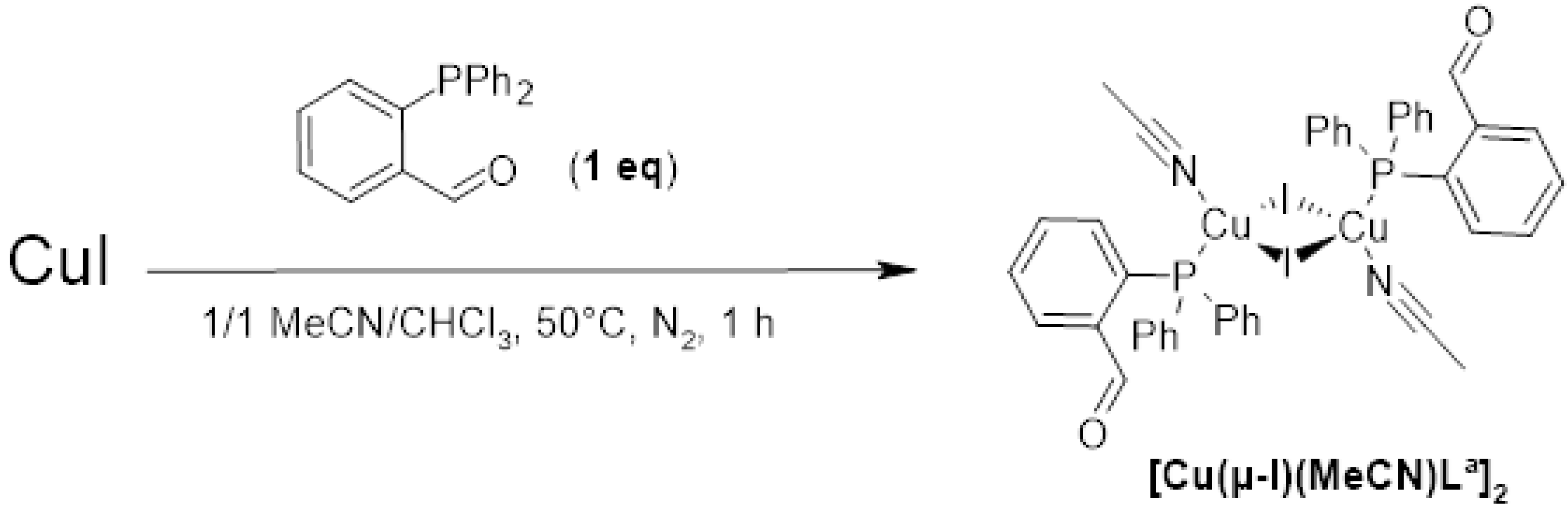

b) FT-IR spectrum of **[Cu(μ-I)(MeCN)L[a]]$_2$**.

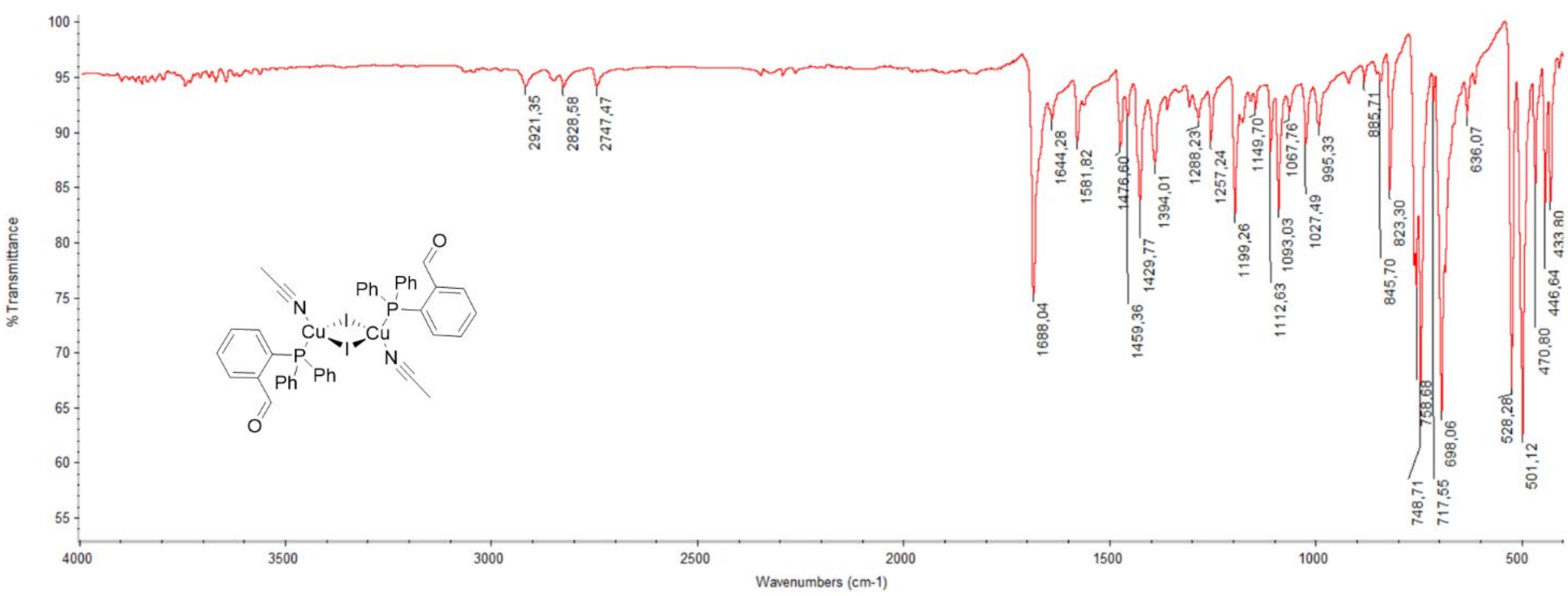

Figure S14: ^{1}H NMR (400 MHz, $CDCl_3$, 298 K) spectrum of **[Cu(μ-I)(MeCN)L^a]₂**.

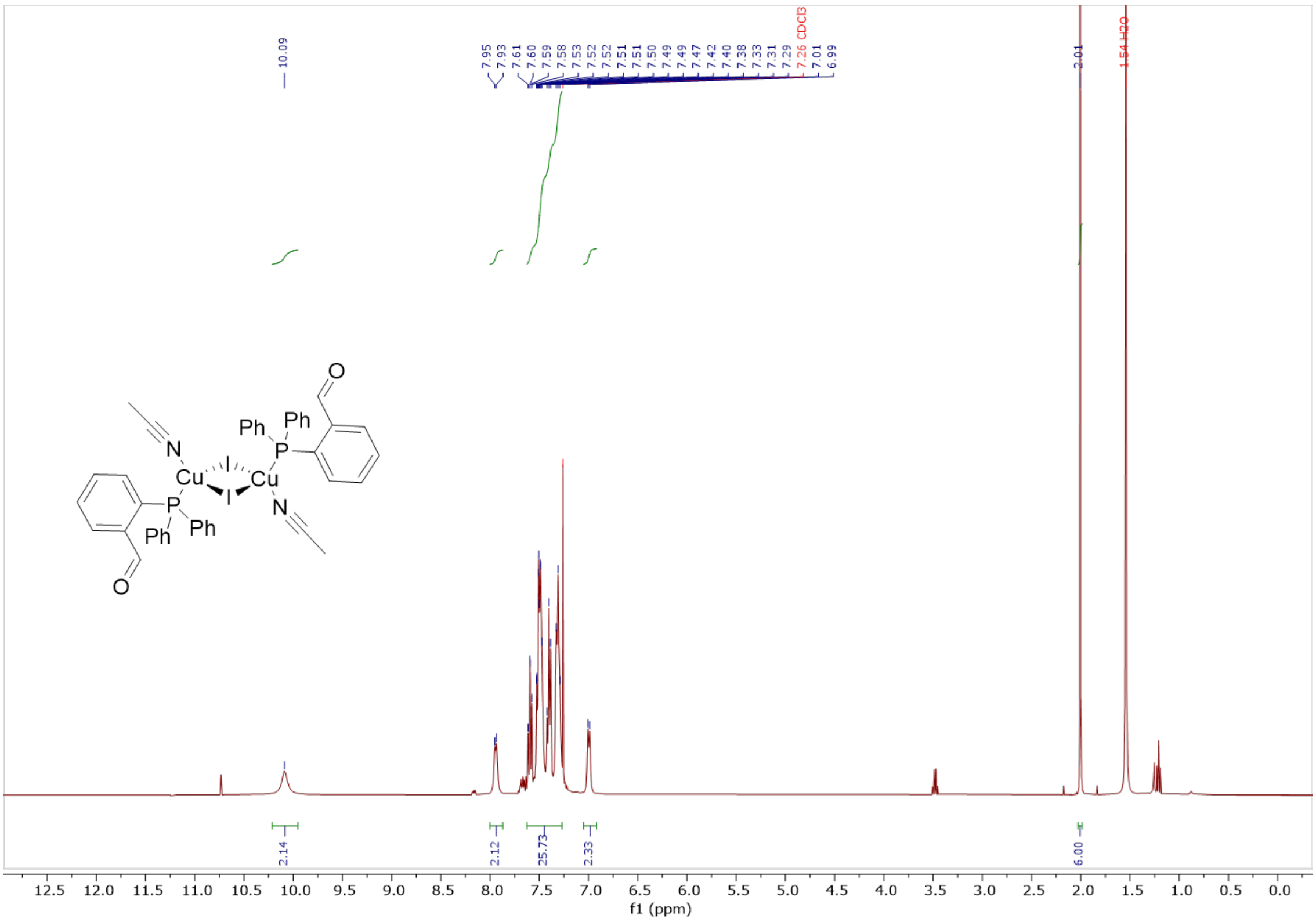

Figure S15: ^{31}P NMR (162 MHz, $CDCl_3$, 298 K) spectrum of **[Cu(μ-I)(MeCN)L^a]₂**.

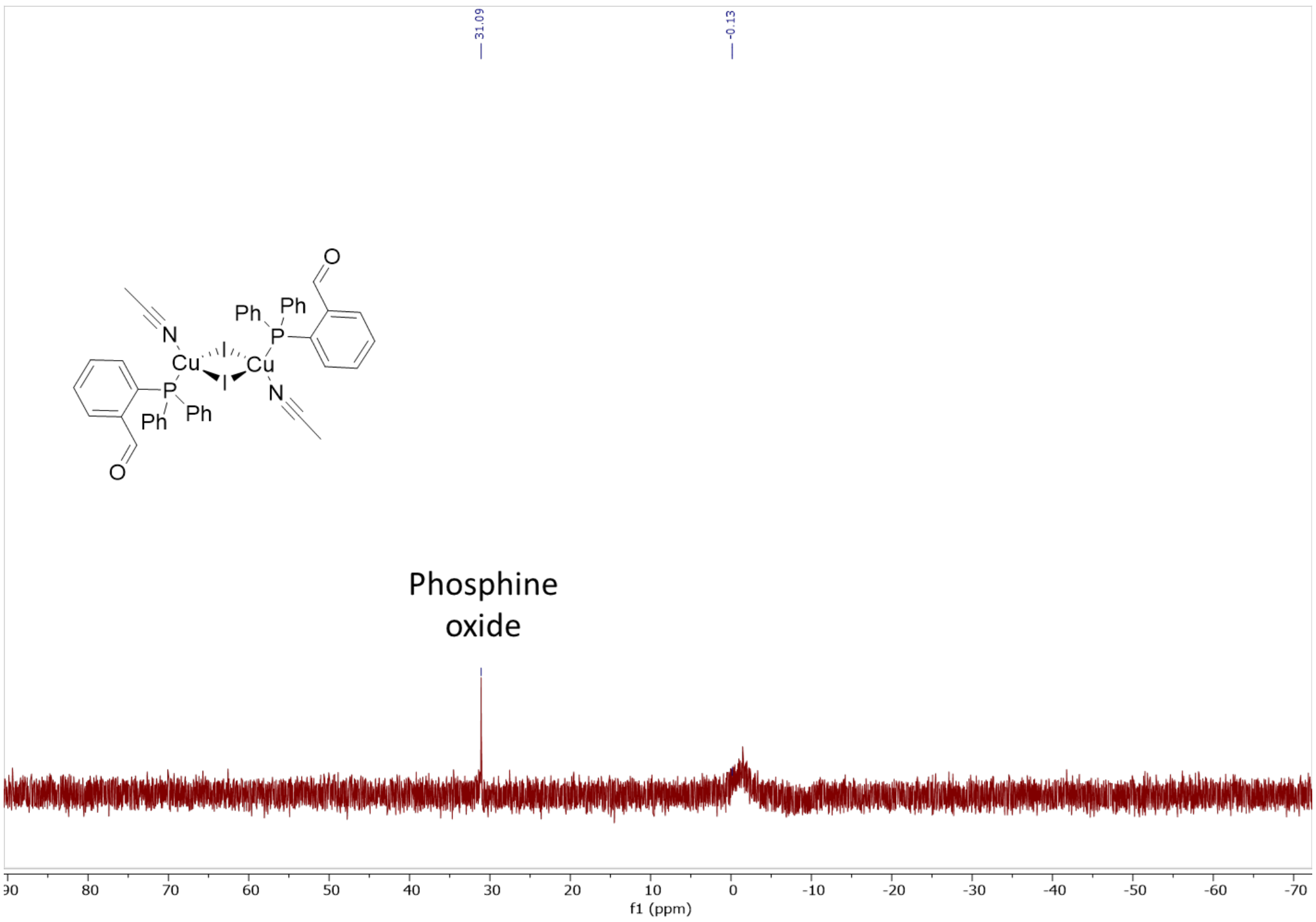

Figure S16: ESI-MS spectrum (positive ions) for **[Cu(μ-I)(MeCN)L^a]$_2$** in acetonitrile.

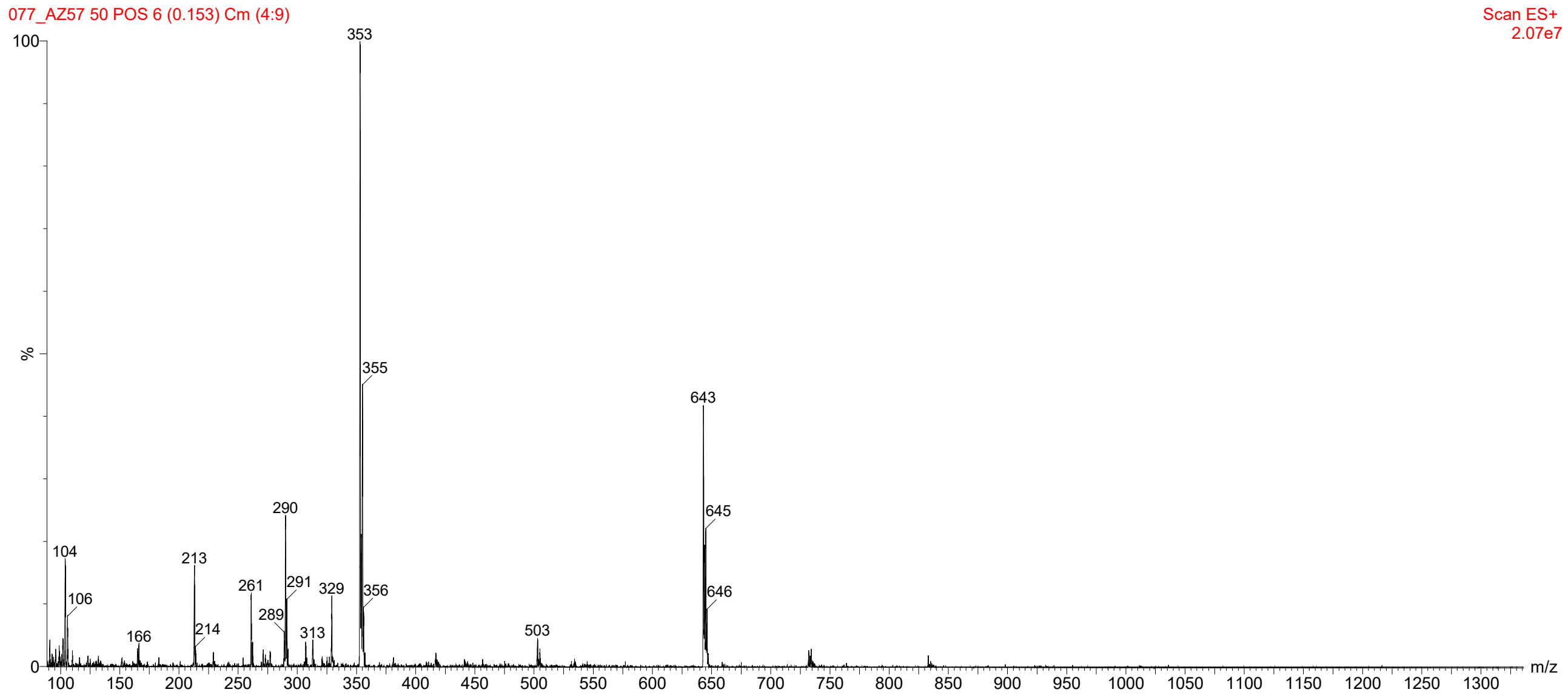

Figure S17: (a) Calculated (in red) and experimental (in black) powder diffractogram of complex **[Cu(μ-I)(MeCN)L^a]$_2$**; (b) ORTEP diagram of the crystal structure of **[Cu(μ-I)(MeCN)L^a]$_2$**, drawn with 50% probability ellipsoids (phosphorus atoms in light orange, nitrogen in blue, carbon in grey, hydrogens in white, iodine in purple, oxygen in red, copper in dark orange; Aguirrechu-Comerón et al., 2018)

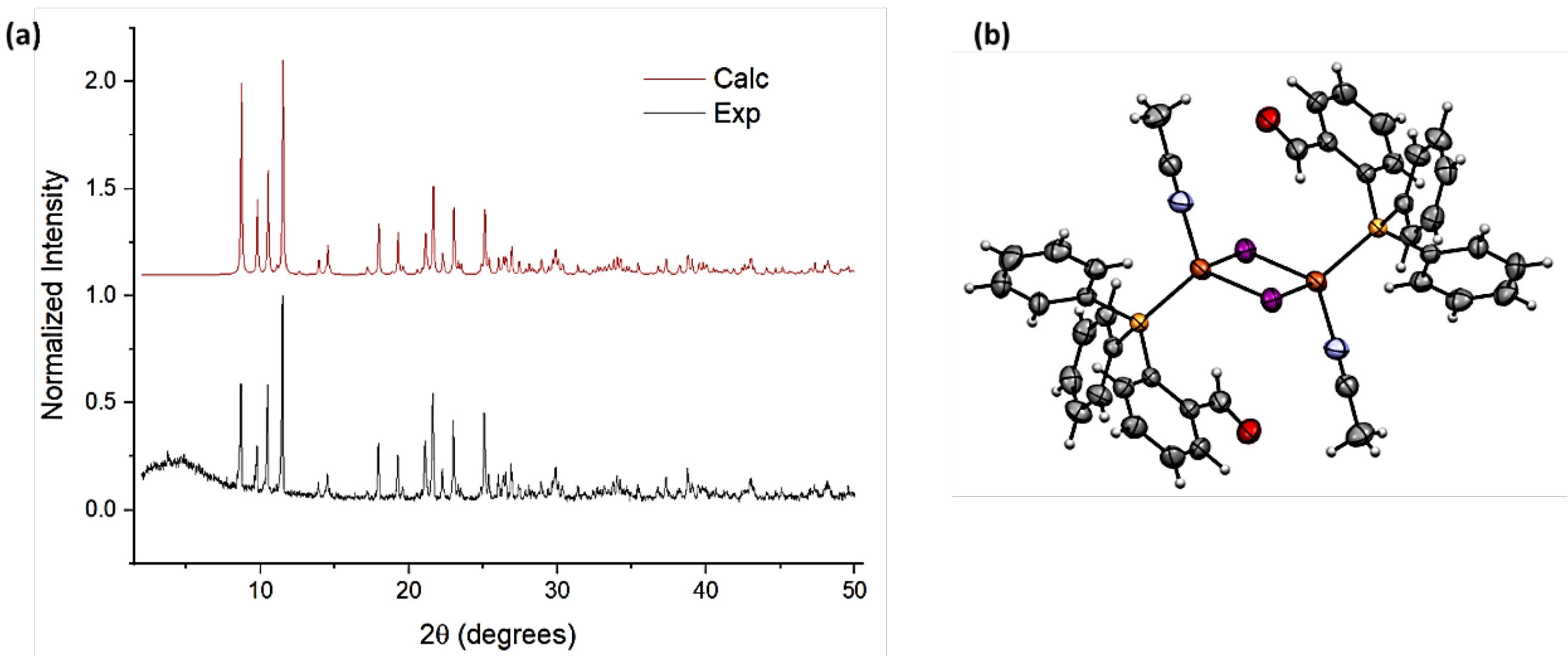

Figure S18: FT-IR spectrum of **(4)**.

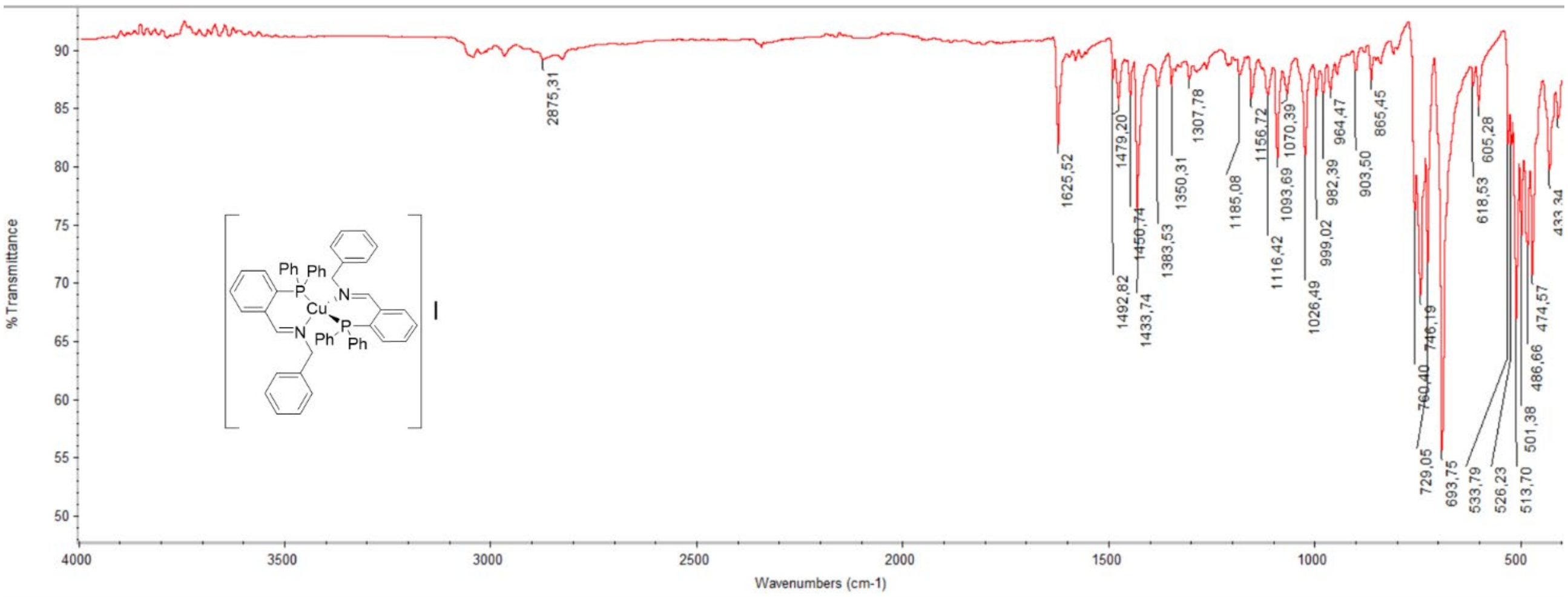

Figure S19: FT-IR spectrum of **(5)**.

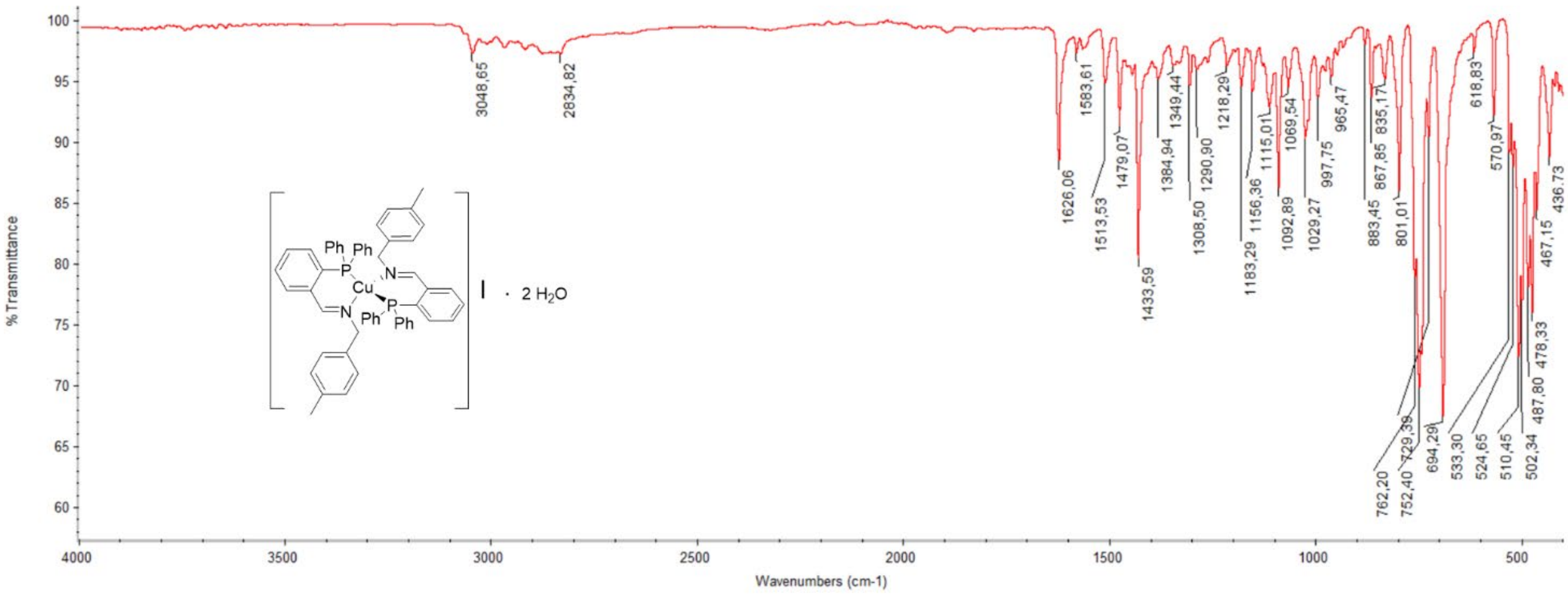

Figure S20: FT-IR spectrum of **(6)**.

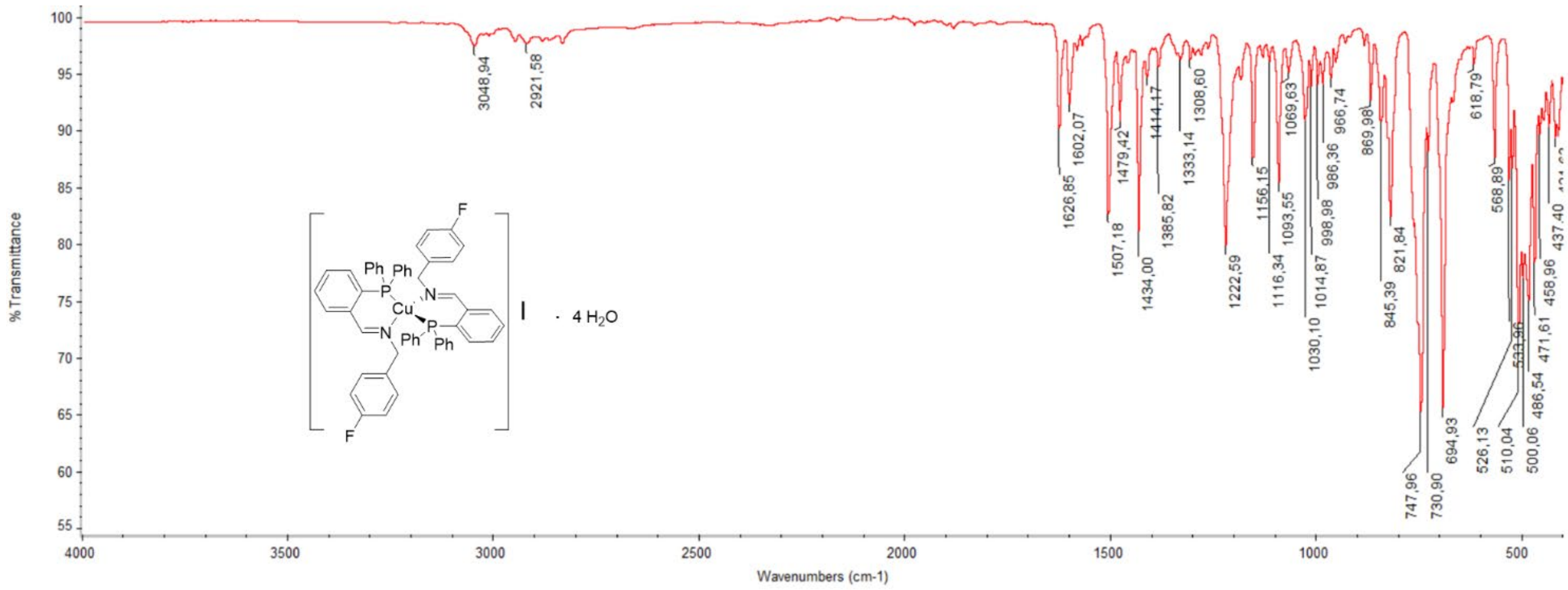

Figure S21: (a) ^{1}H NMR (400 MHz, 298 K) and (b) ^{31}P NMR (162 MHz, 298 K) spectra of **L^{1}** (top), complex **(1)** (middle), and complex **(4)** (bottom) in $CDCl_3$.

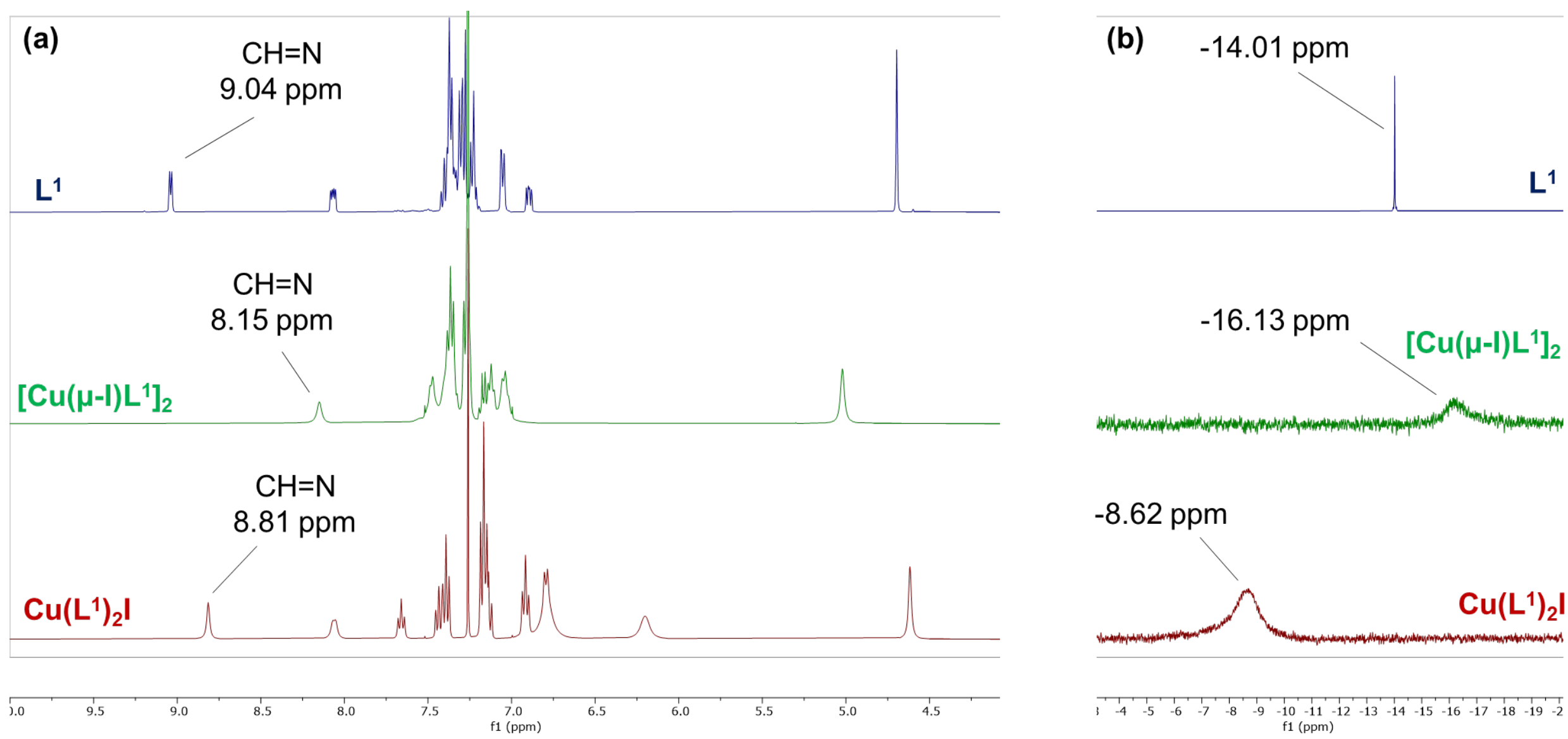

Figure S22: (top) ^{1}H NMR (400 MHz, $CDCl_3$, 298 K) spectrum of **(4)** immediately after preparation of the sample (purple), after one day (blue-green), after two days (green) and after one week (red). (bottom) ^{31}P NMR (162 MHz, $CDCl_3$, 298 K) spectrum of complex **(4)** immediately after preparation of the sample (purple), after one day (blue-green), after two days (green) and after one week (red).

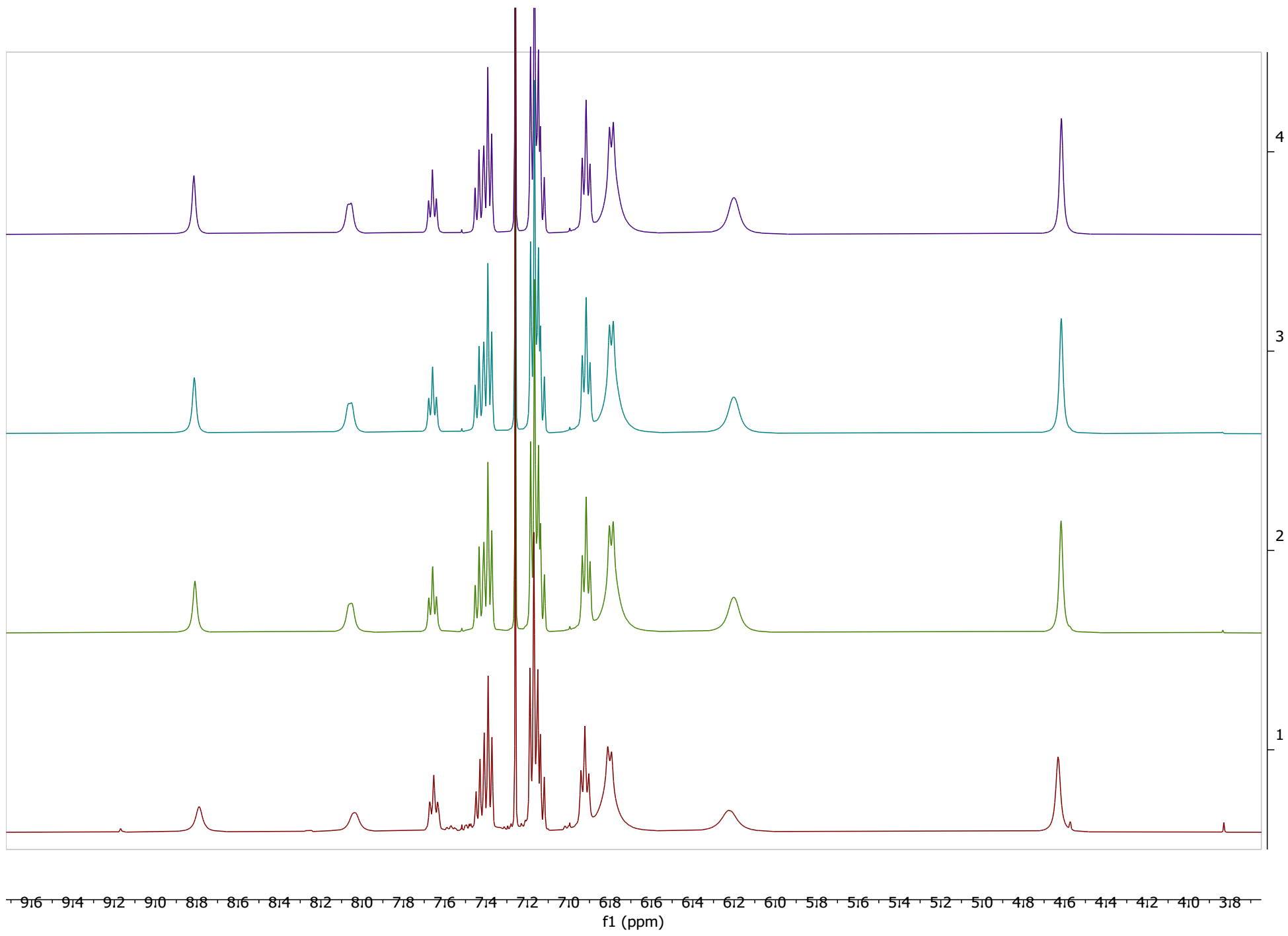

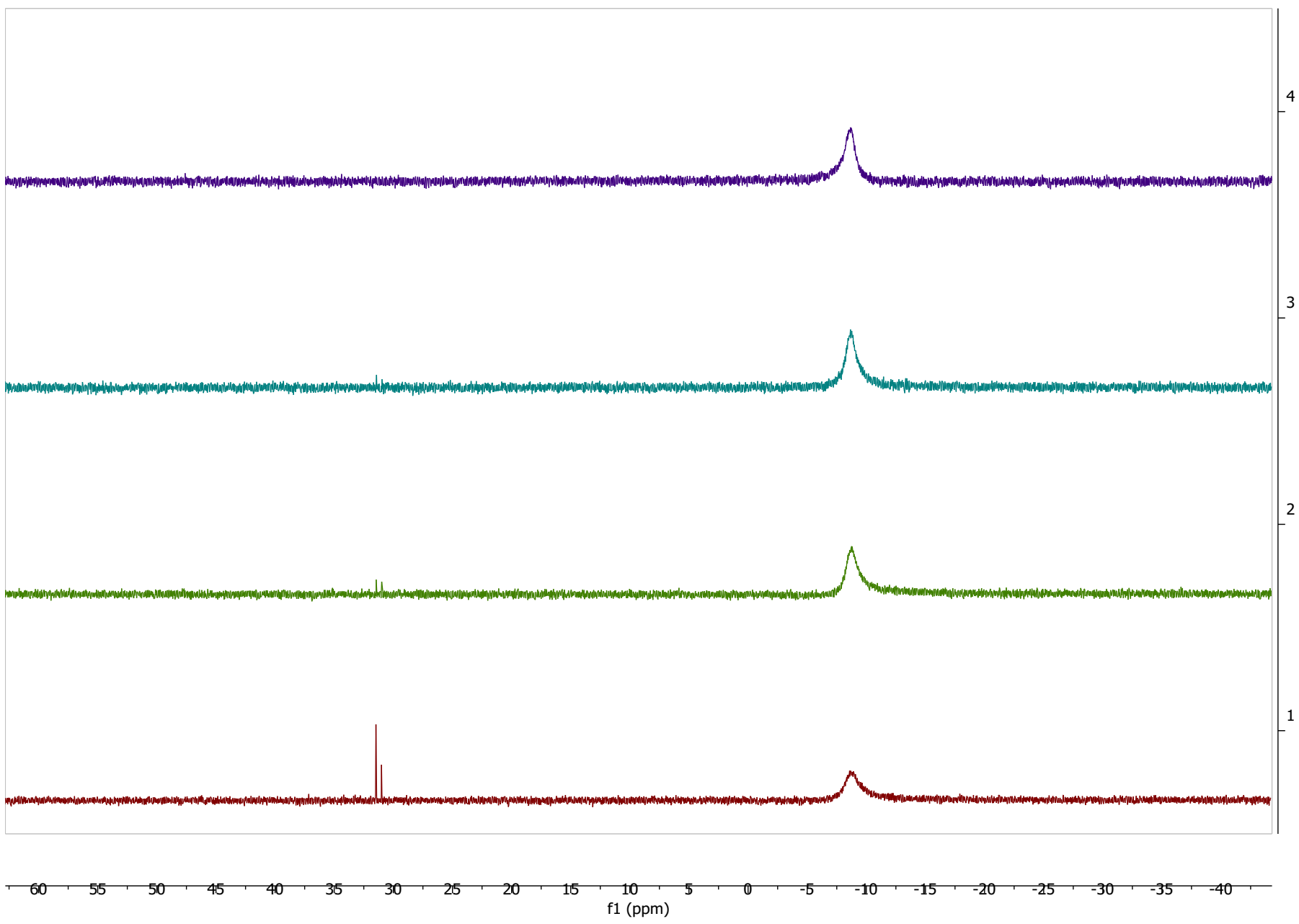

Figure S23: ^{1}H NMR (400 MHz, DMSO-d$_6$, 298 K) spectrum of **(4)**.

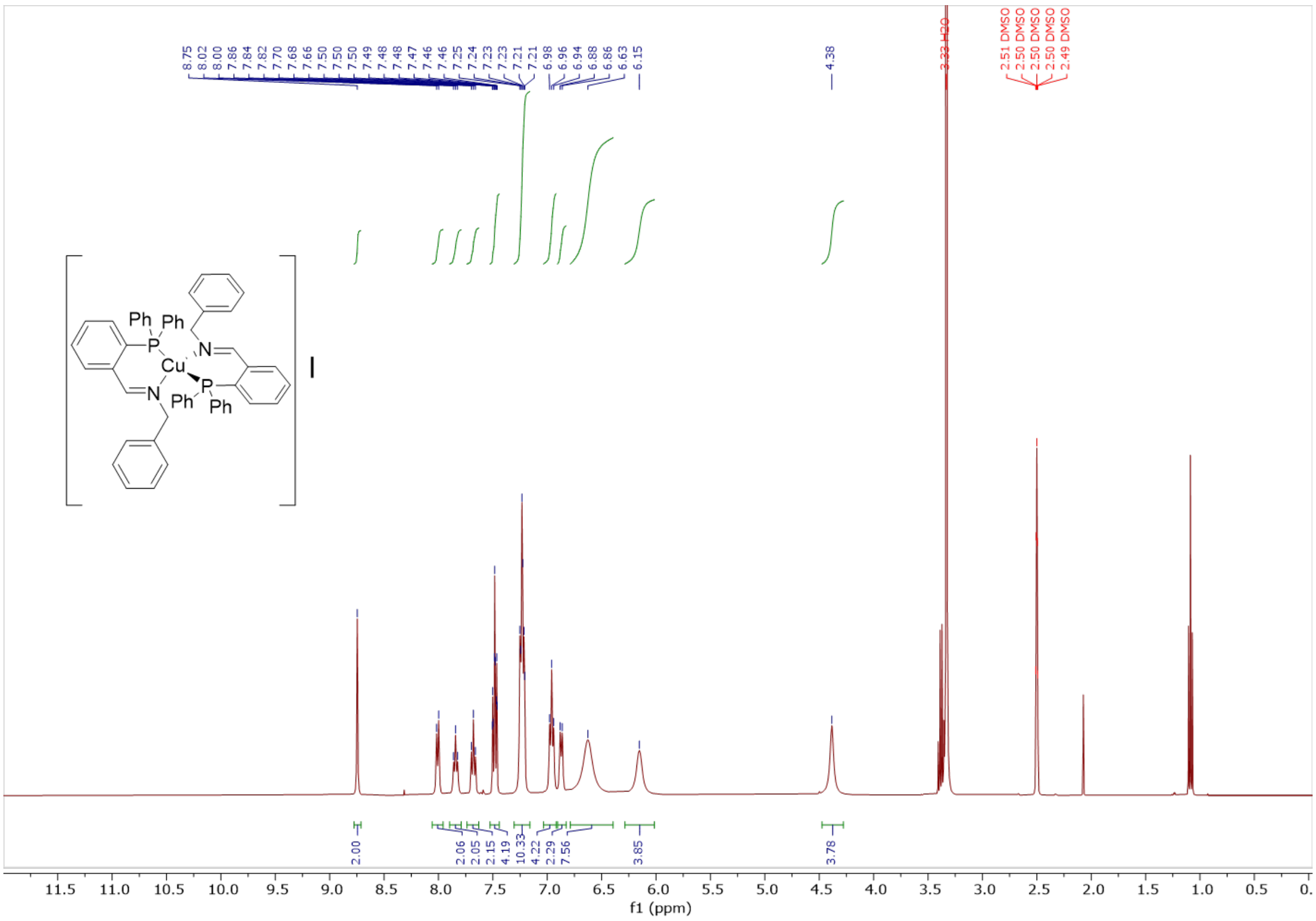

Figure S24: ^{1}H NMR (400 MHz, DMSO-d$_6$, 298 K) spectrum of **(4)** immediately after preparation of the sample (top), after one day (middle) and after two days (bottom).

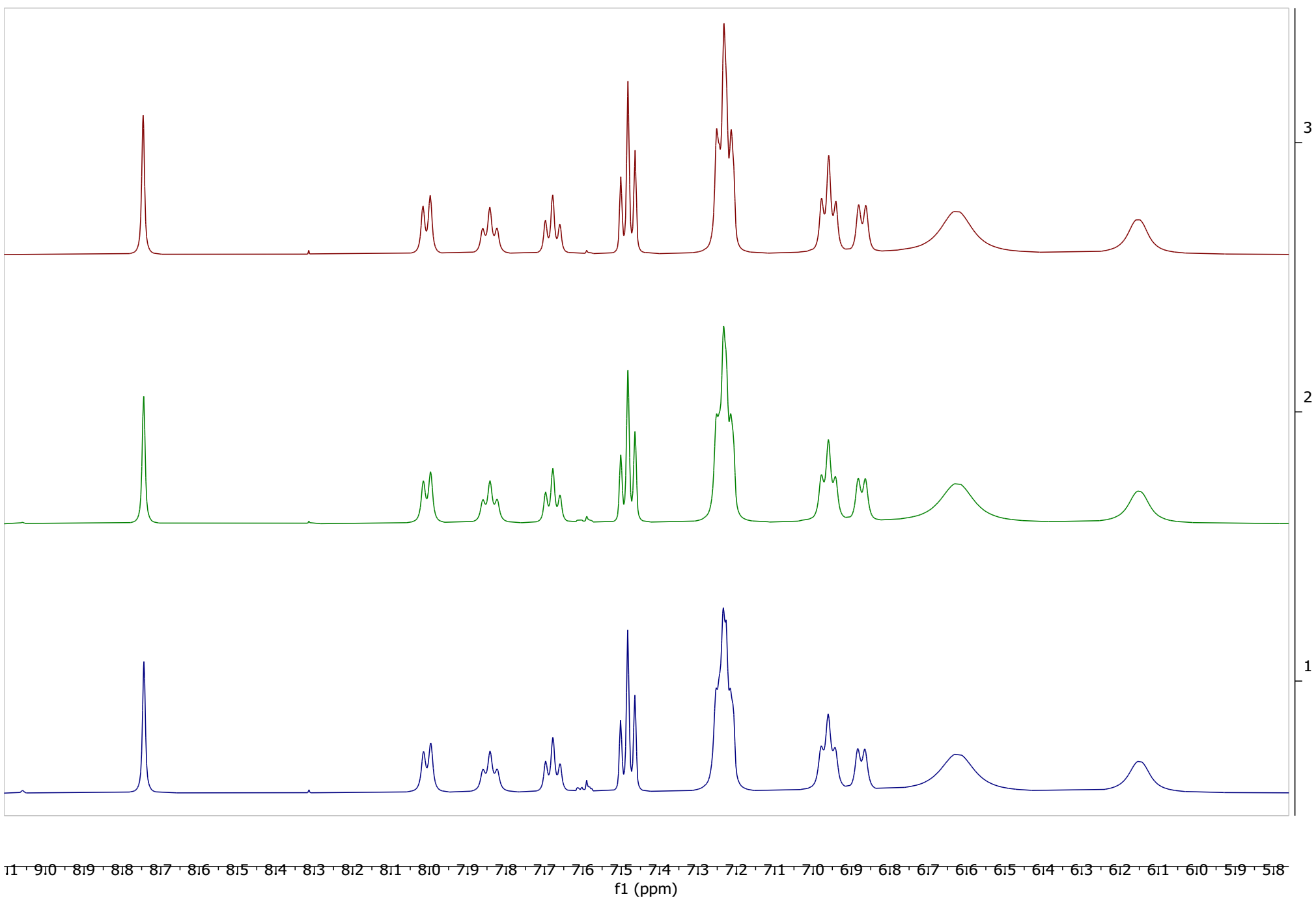

Figure S25: ^{1}H NMR (400 MHz, DMSO-d_6, 298 K) spectrum of **(5)**.

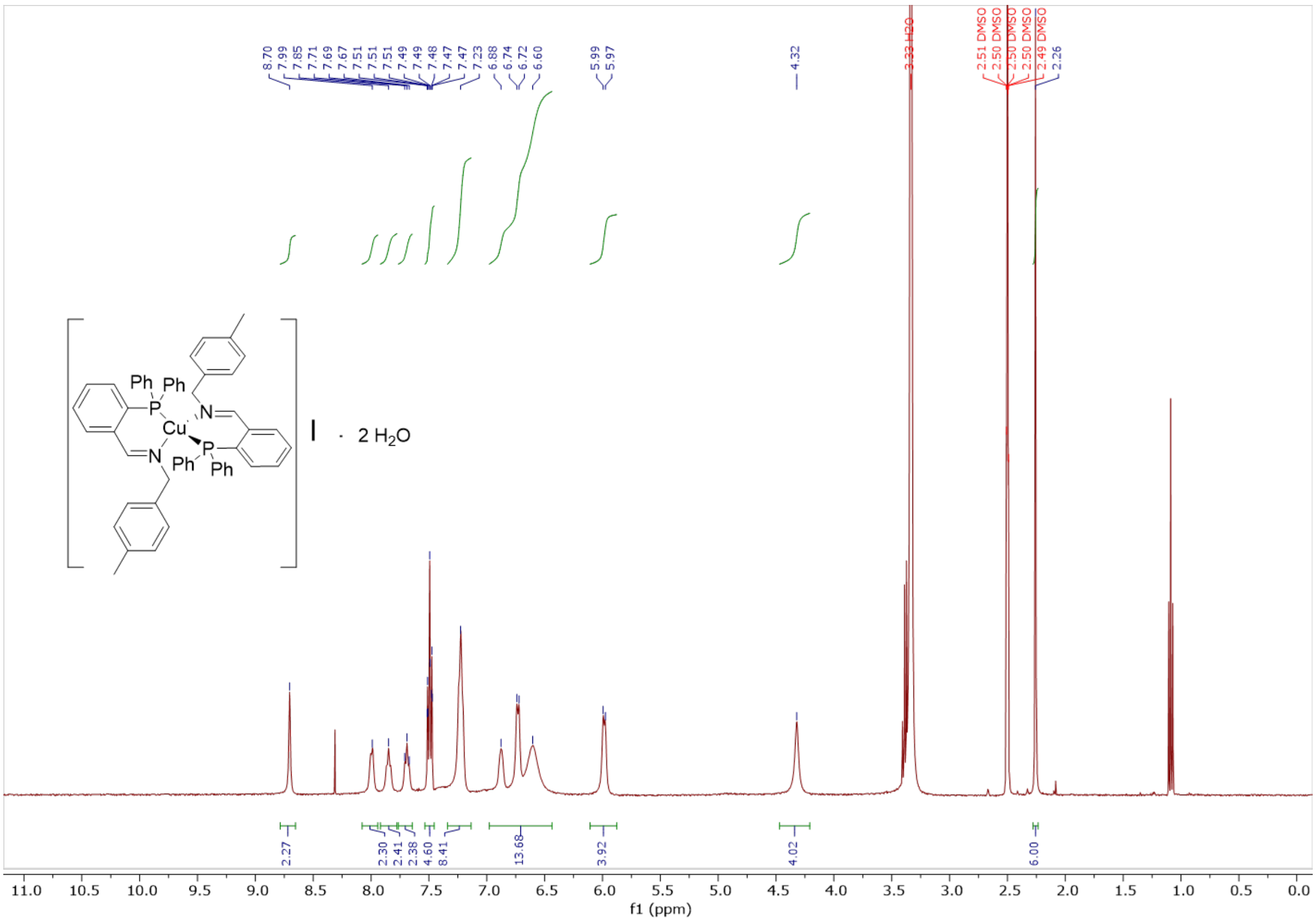

Figure S26: ^{31}P NMR (162 MHz, DMSO-d_6, 298 K) spectrum of **(5)**.

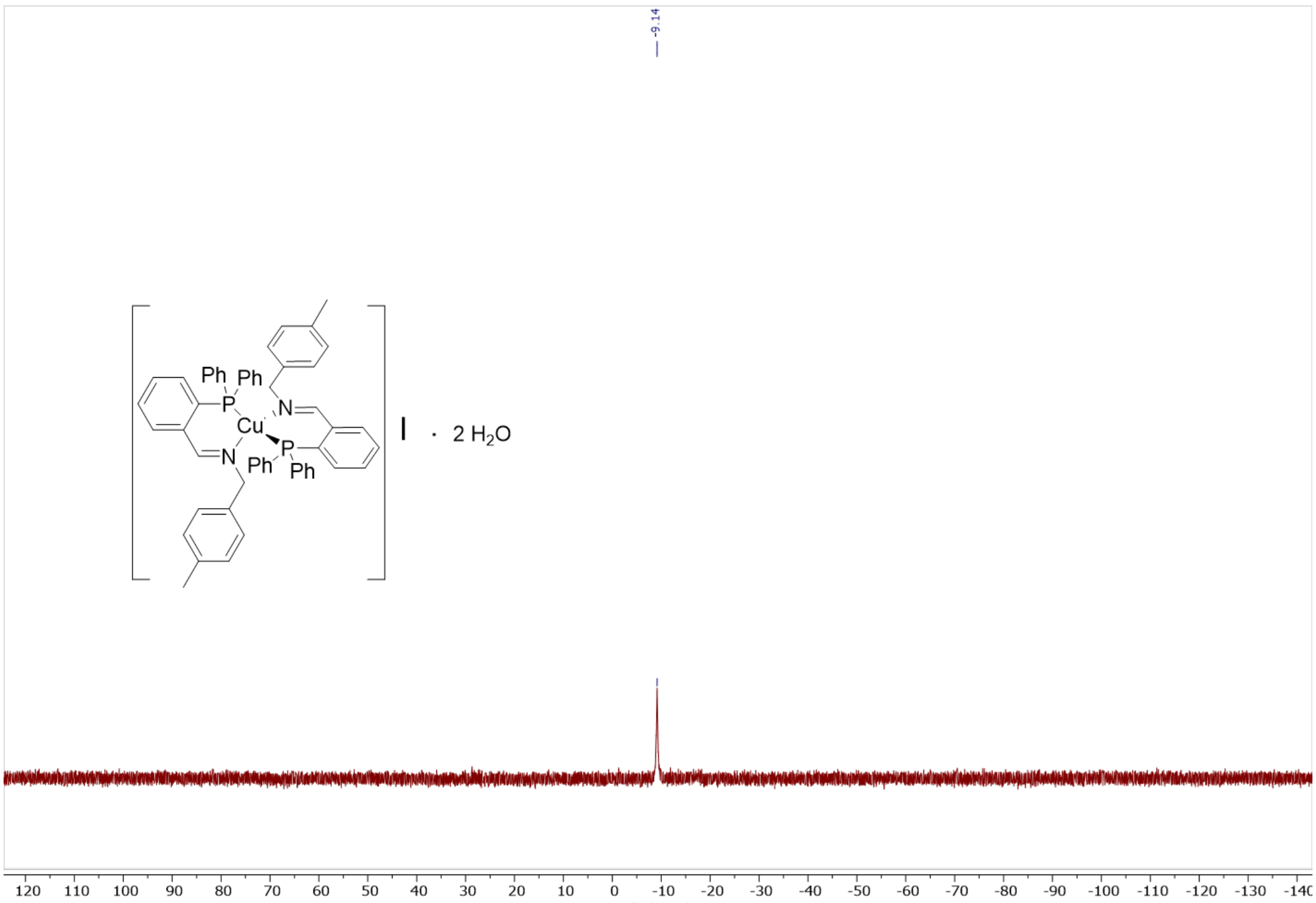

Figure S27: ^{1}H NMR (400 MHz, DMSO-d_6, 298 K) spectrum of **(6)**.

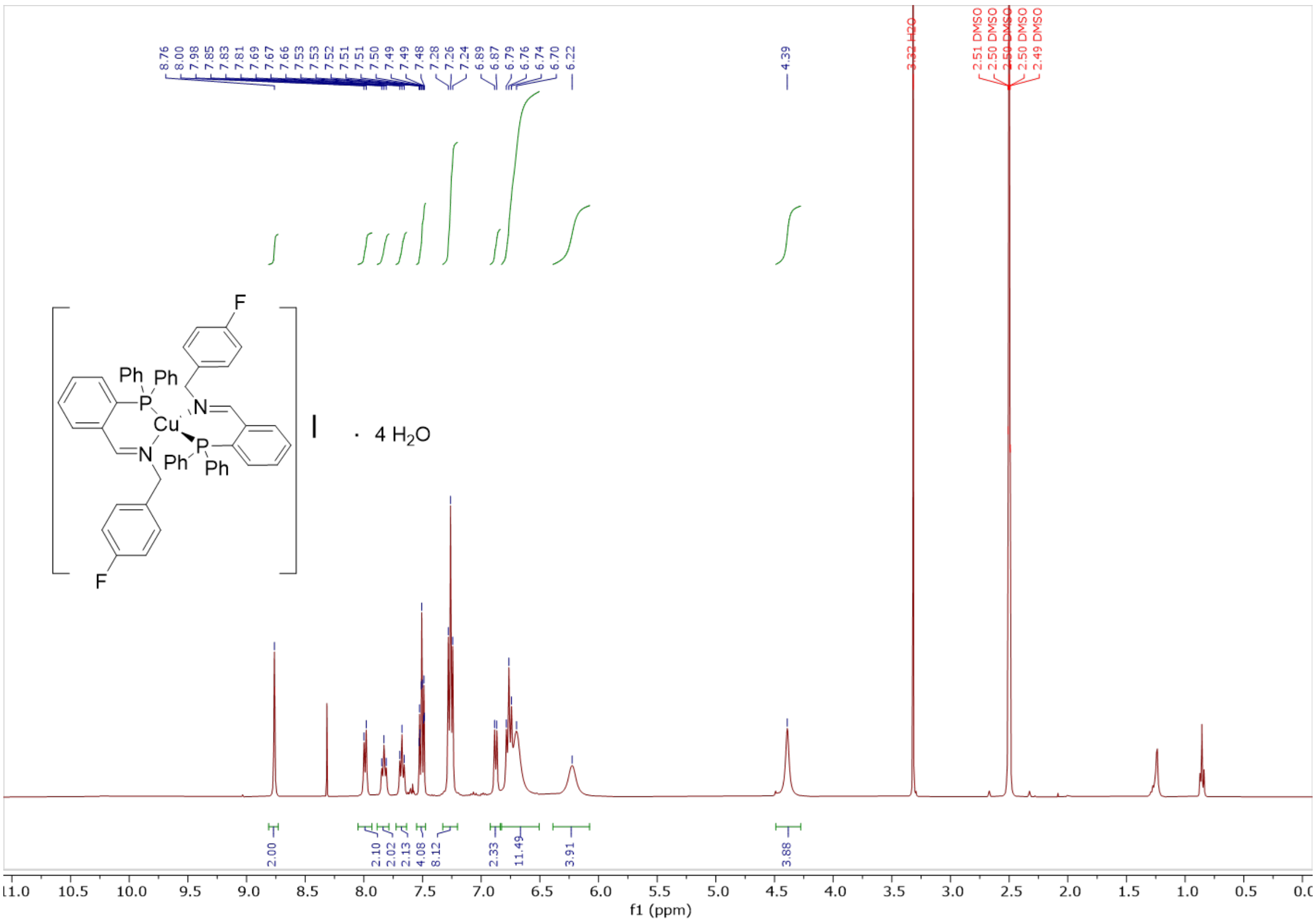

Figure S28: ^{31}P NMR (202 MHz, DMSO-d_6, 298 K) spectrum of **(6)**.

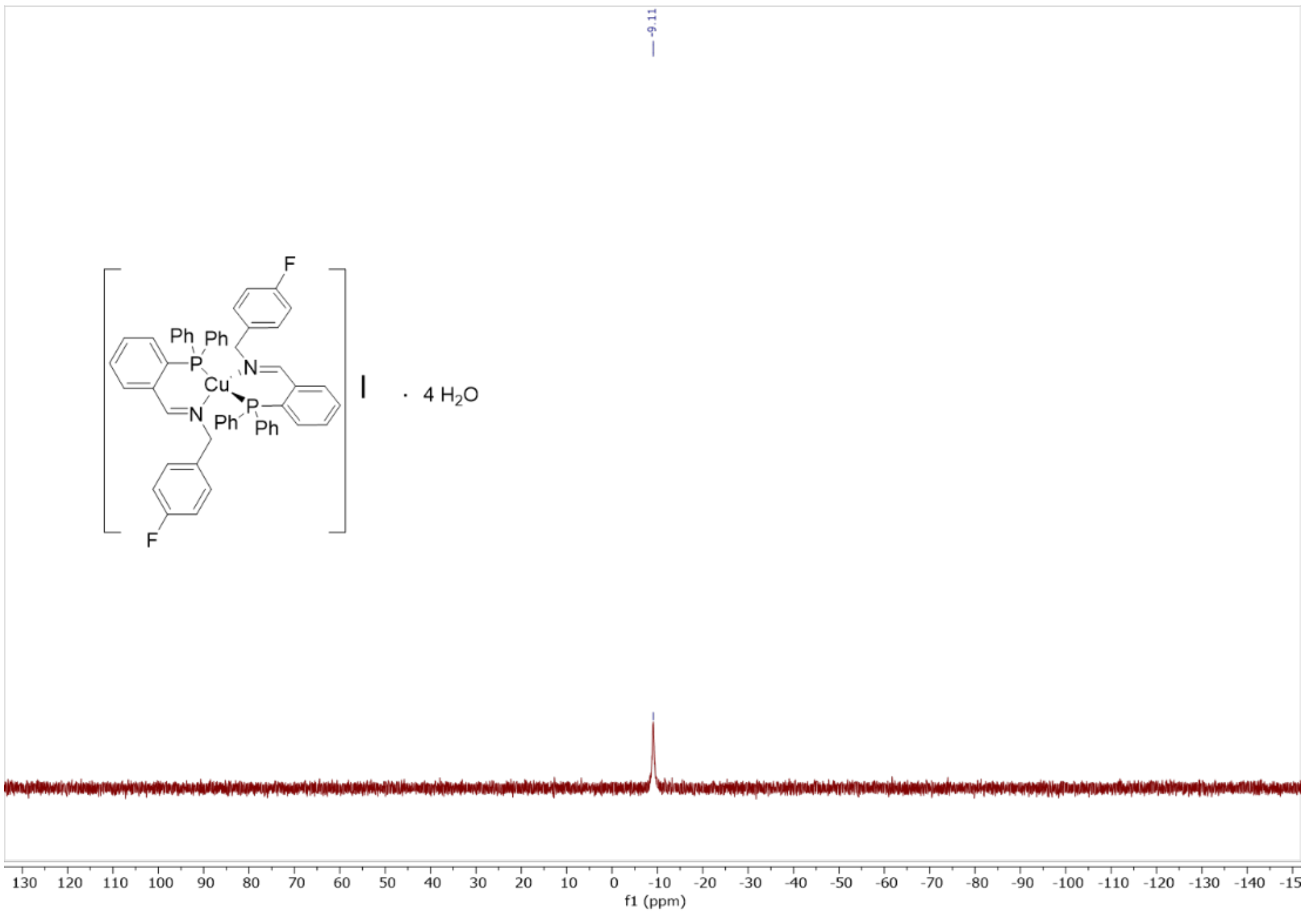

Figure S29: ESI-MS spectrum (positive ions) for **(4)** in acetonitrile.

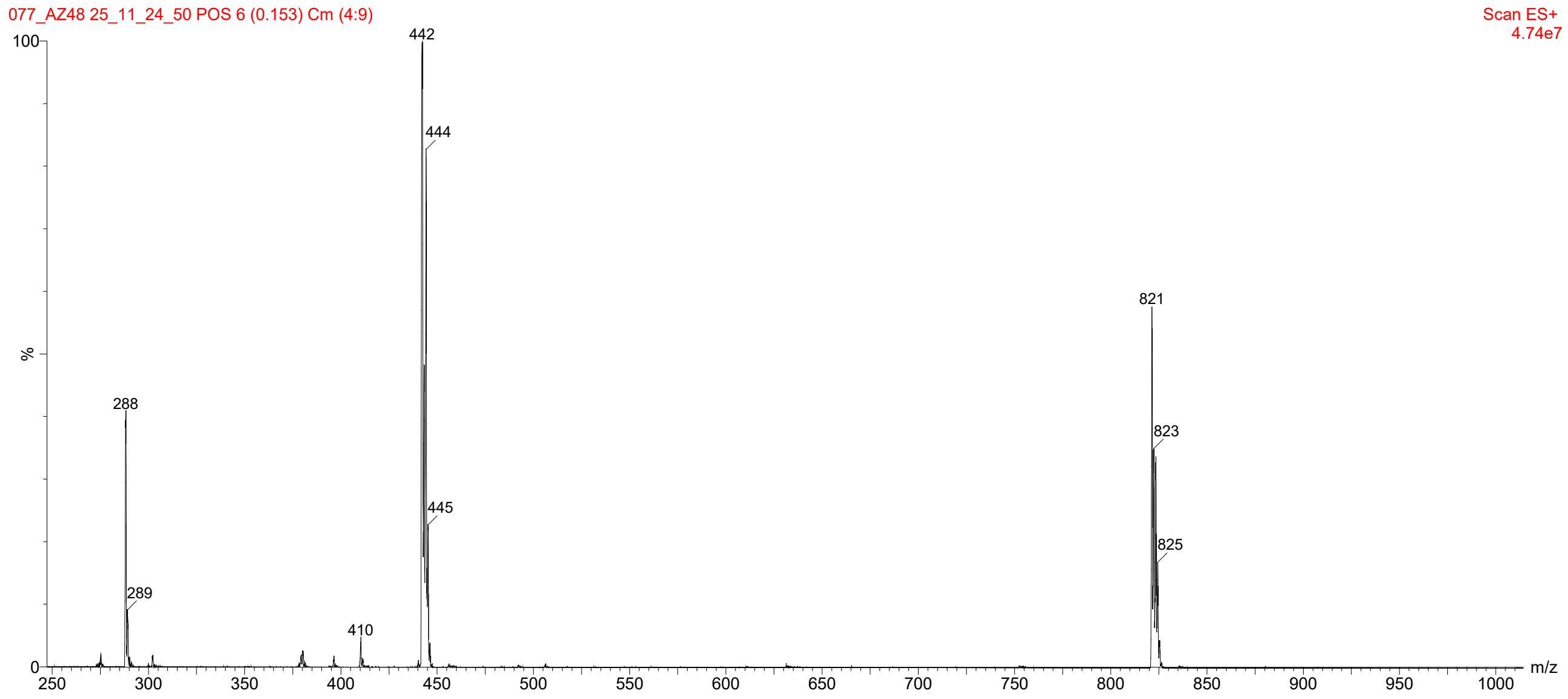

Figure S30: ESI-MS spectrum (positive ions) for **(5)** in acetonitrile.

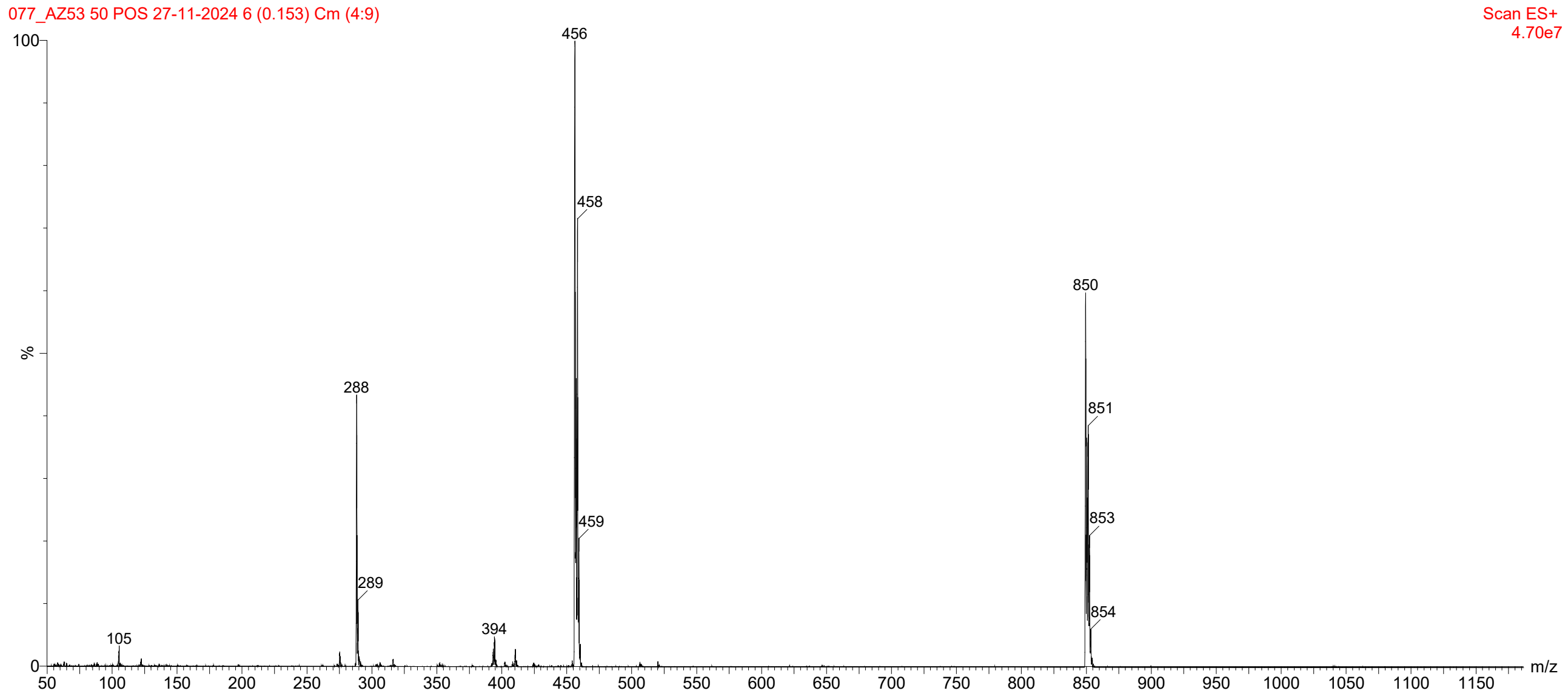

Figure S31: ESI-MS spectrum (positive ions) for **(6)** in acetonitrile.

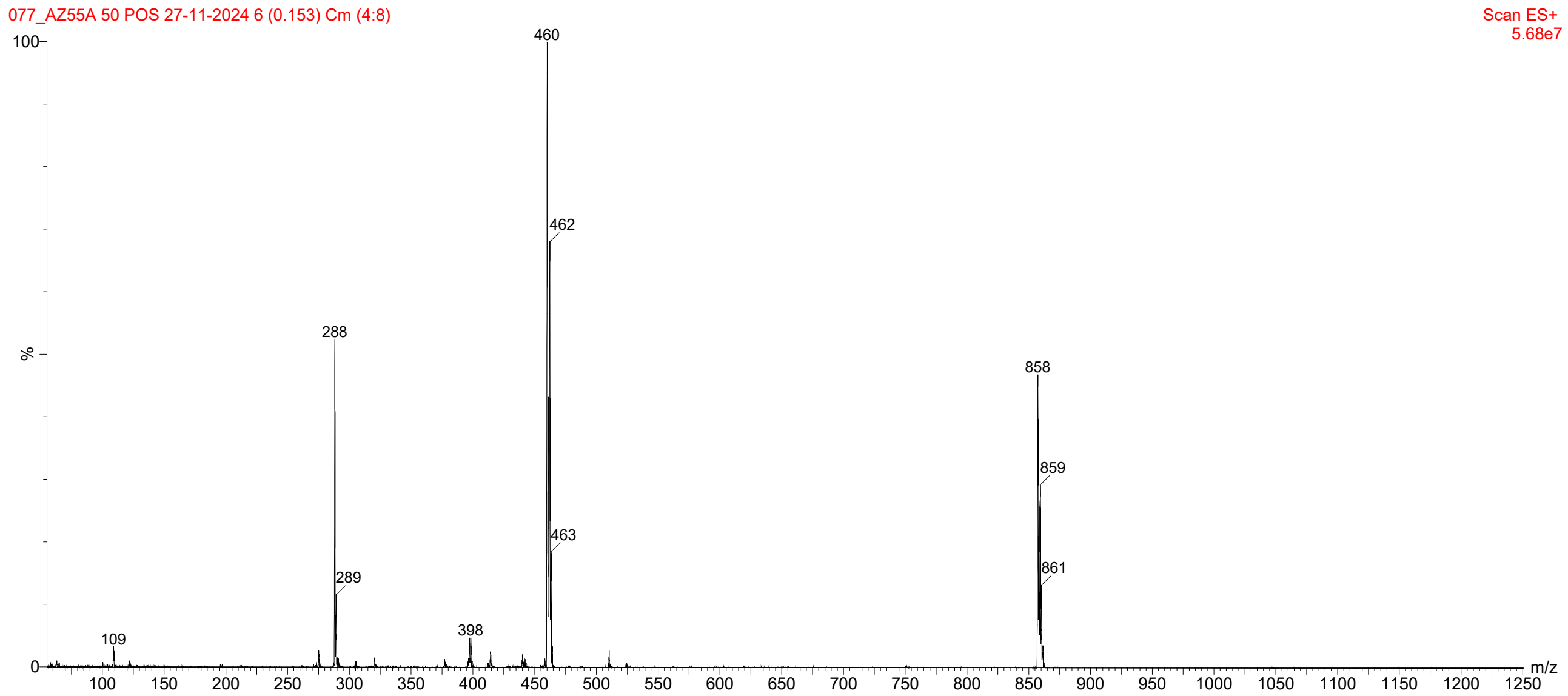

Figure S32: X-ray crystallographic data and ORTEP representation for compound **(5a)**

**Empirical formula**	C54 H48 Cu I N2 P2
**Formula weight**	977.32
**Temperature/K**	200.0
**Diffractometer/detector**	Bruker D8 Venture / PhotonII area detector
**Radiation**	CuKα (λ = 1.54178)
**Crystal system**	triclinic
**Space group**	P-1
**a/Å**	11.595(17)
**b/Å**	13.058(18)
**c/Å**	16.76(3)
**α/°**	92.44(10)
**β/°**	106.06(12)
**γ/°**	105.07(5)
**Volume/Å^3**	2337(7)
**Z**	2
**ρcalc /g·cm^{-3}**	1.389
**μ/mm^{-1}**	6.776
**F(000)**	996
**Θ range for data collection/°**	2.76 - 66.60
	-13<h<13
**Index ranges**	-15<k<15
	-19<l<19
**Reflections collected**	39324
**Unique reflections**	8235
**Parameters**	543
**Goodness-of-fit on F^2**	1.053
**Final R indexes [I>=2σ (I)]**	R=0.037, wR2=0.099
**CCDC Number**	2560045

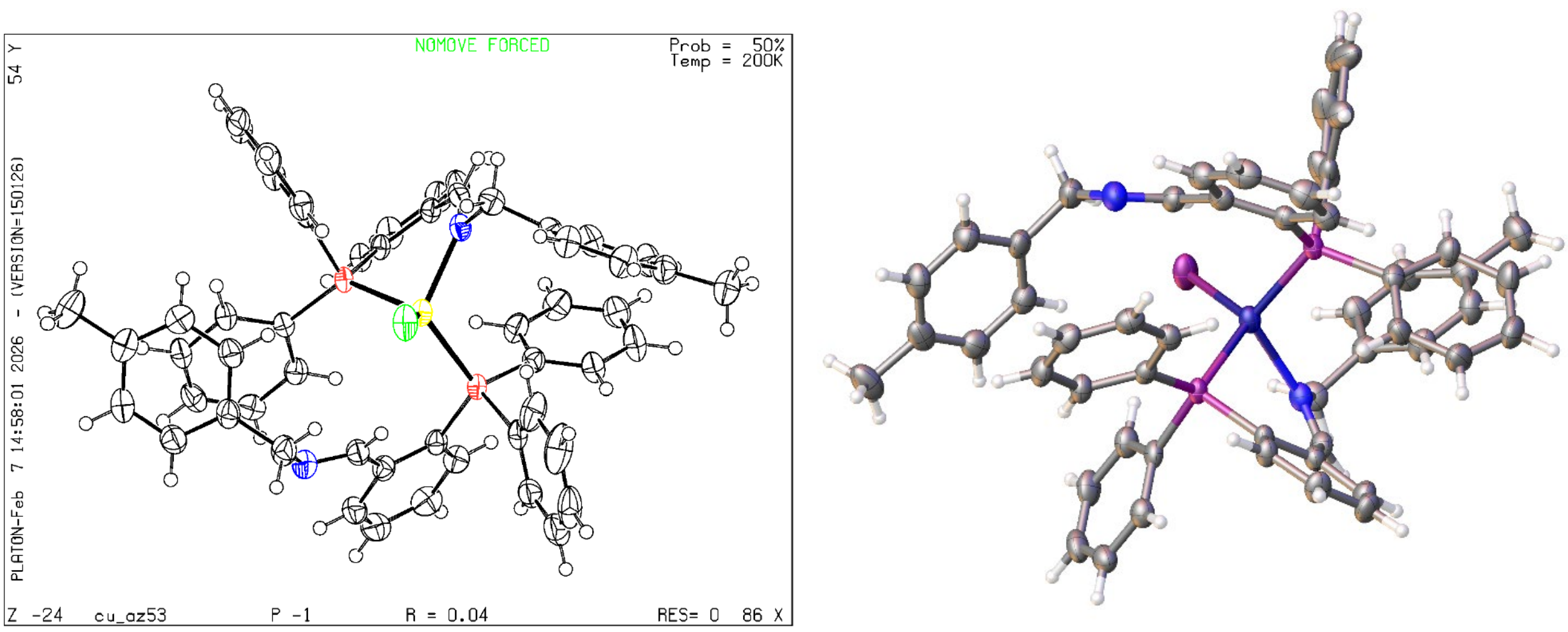

Figure S33: FT-IR spectrum of [**Cu(L^a)_2I**].

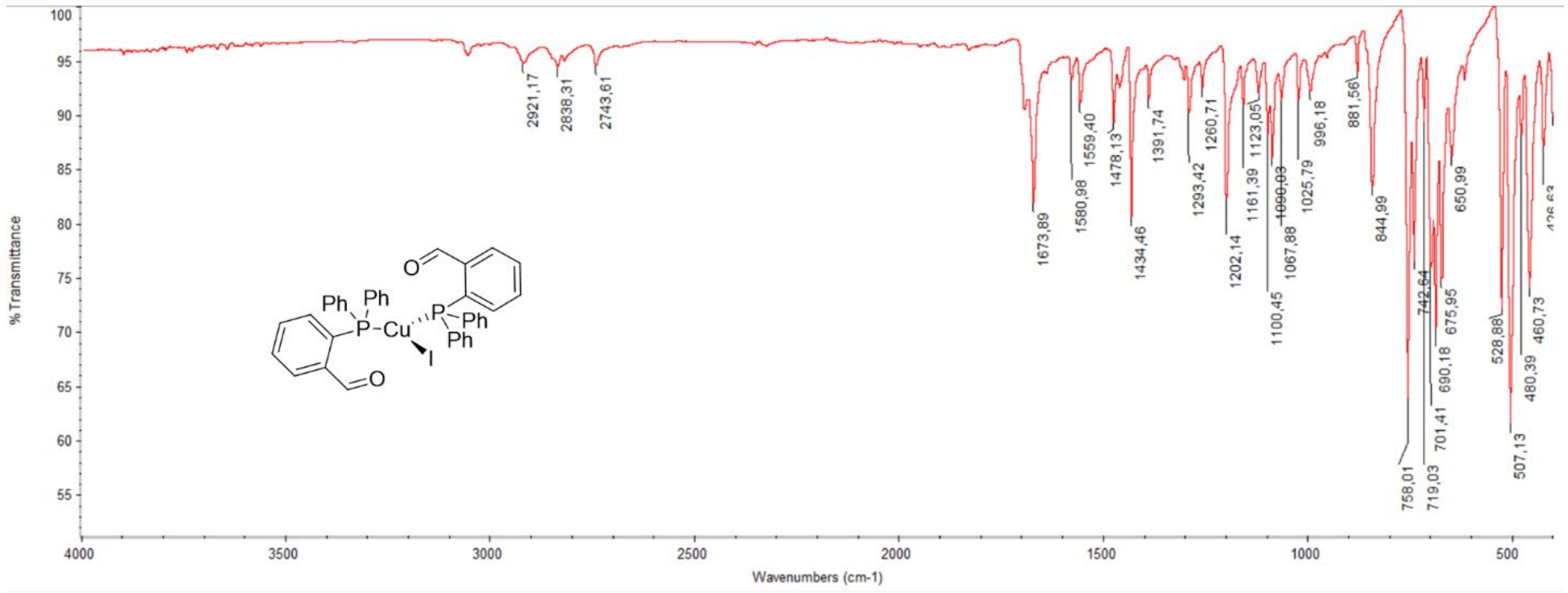

Figure S34: (top) ^{1}H NMR (400 MHz, 298 K) and (bottom) ^{31}P NMR (162 MHz, 298 K) spectra of **[Cu(L^a)_2I]** in $CDCl_3$

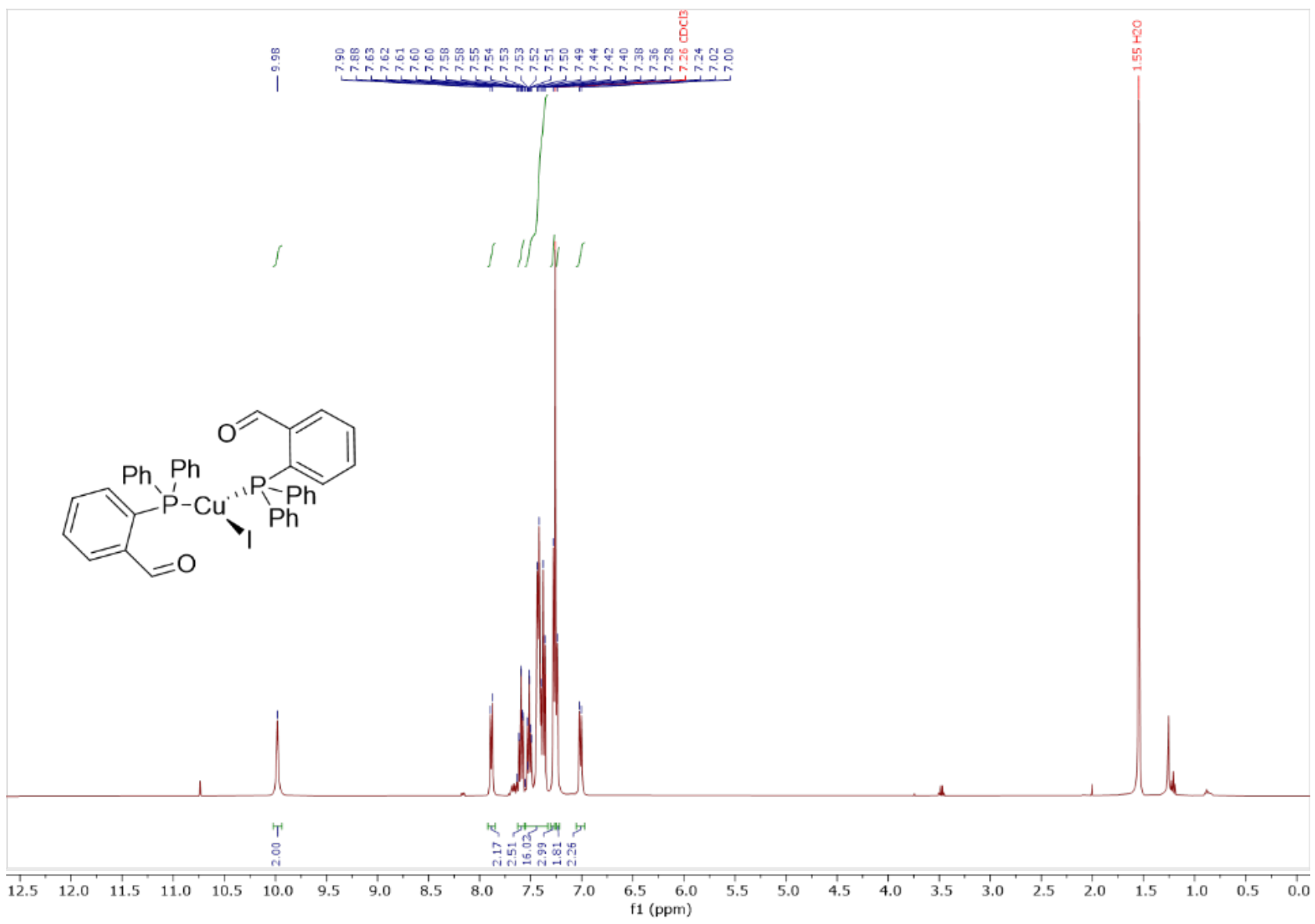

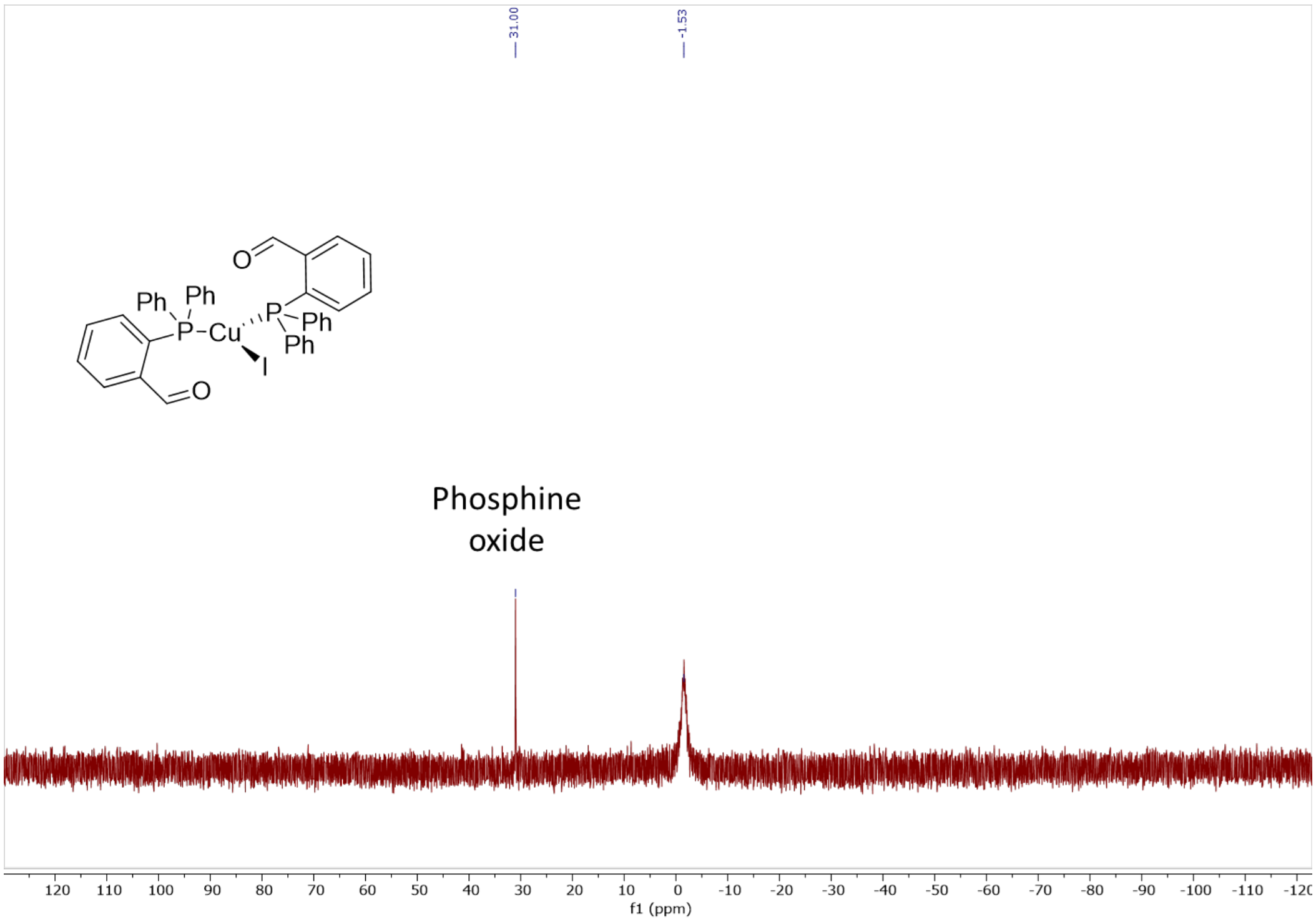

Figure S35: ESI-MS spectrum (positive ions) for **[Cu(L^a)$_2$I]** in acetonitrile.

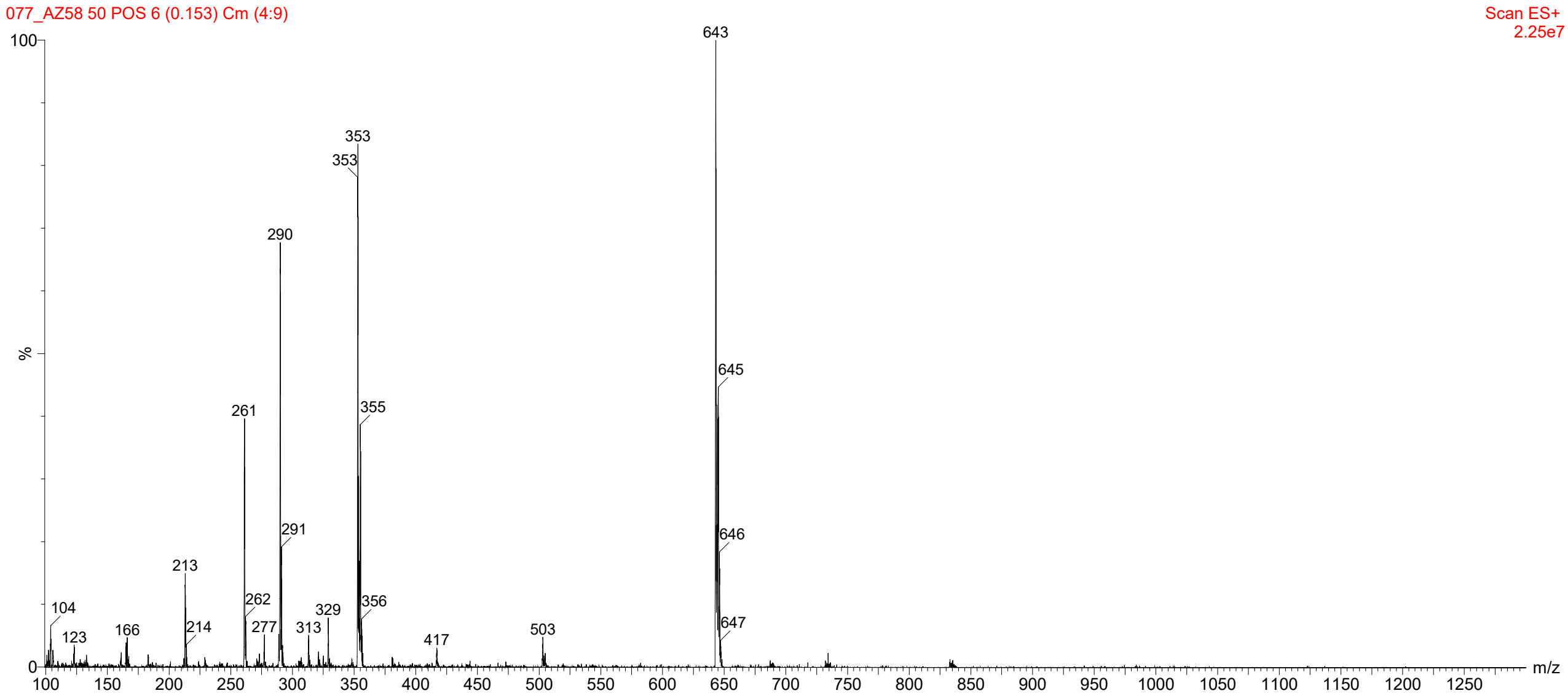

Figure S36: (top) FT-IR spectra of the complex **(7)** (in red) and $\mathbf{L^1}$ (in blue); (bottom) FT-IR spectrum of **(7)**.

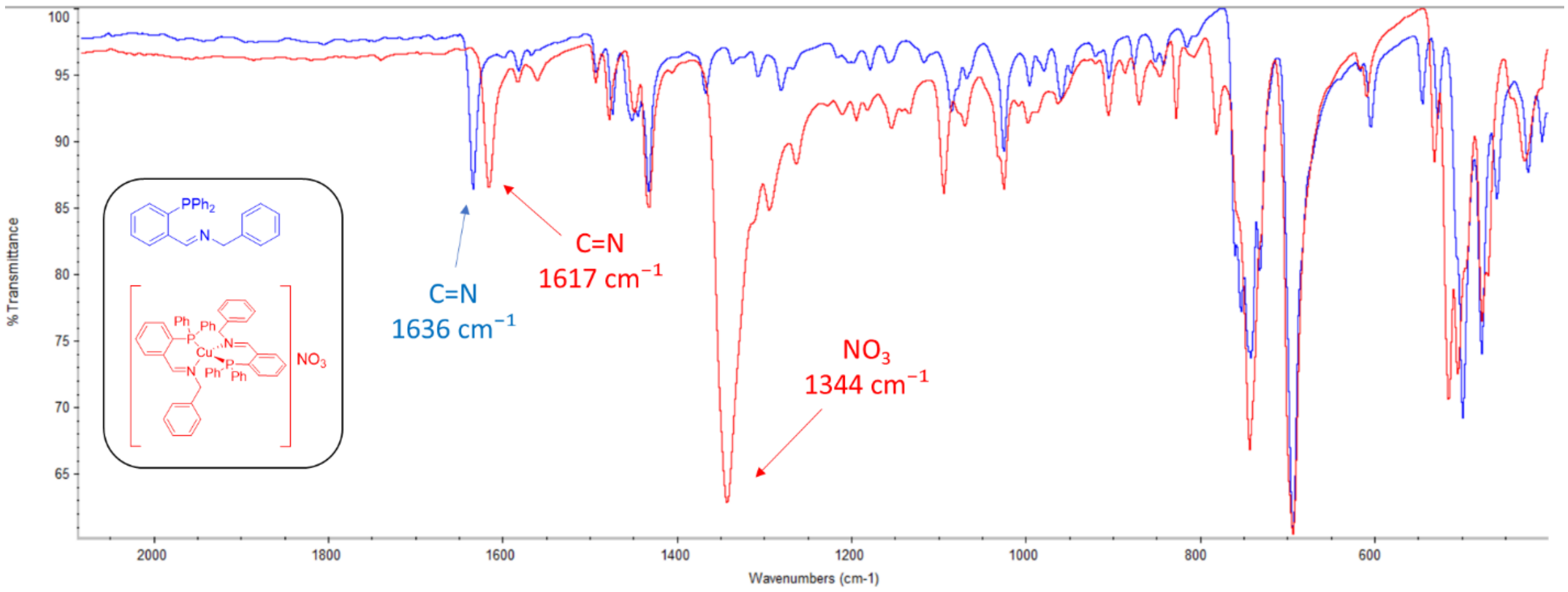

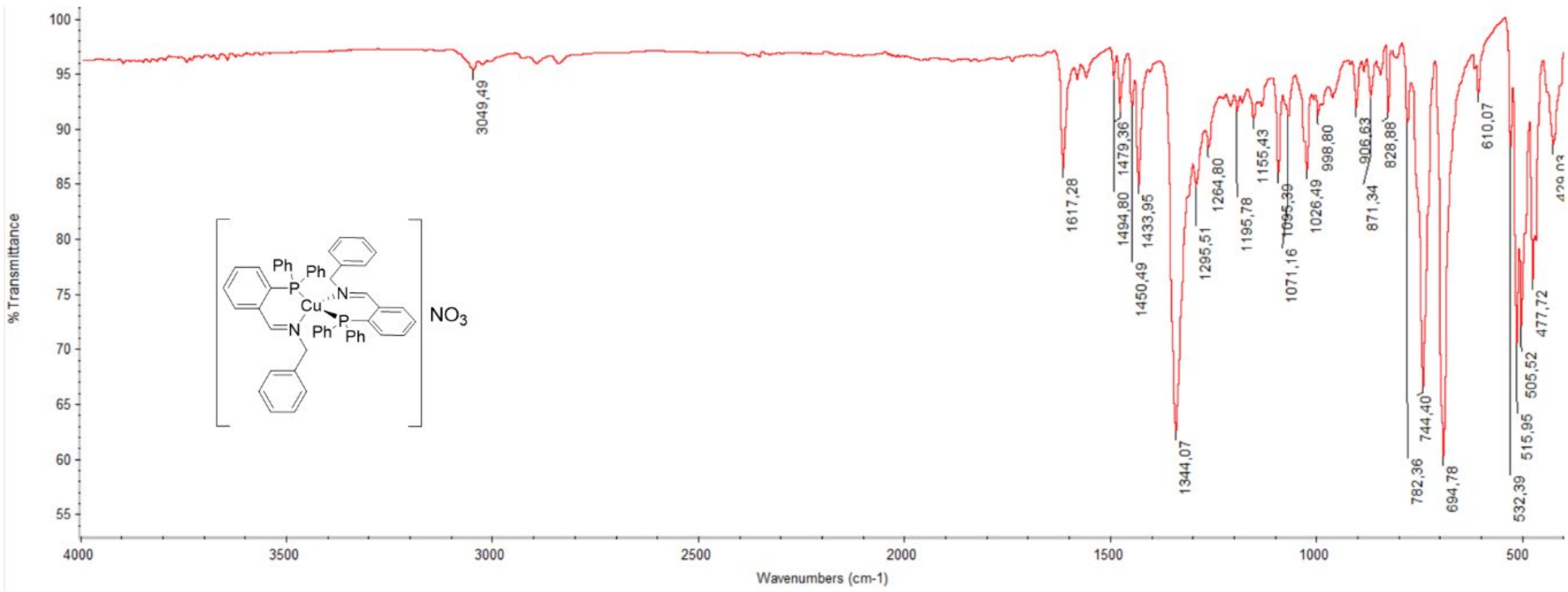

Figure S37: FT-IR spectrum of **(8)**.

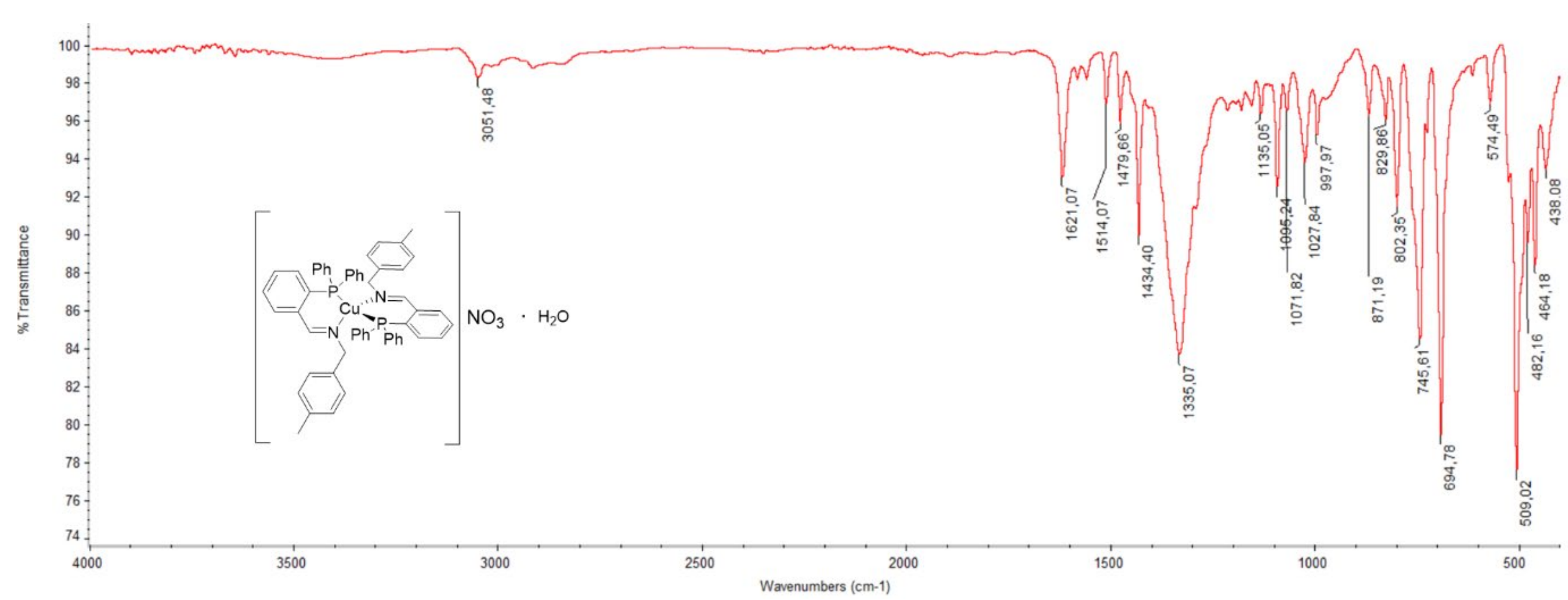

Figure S38: FT-IR spectrum of **(9)**.

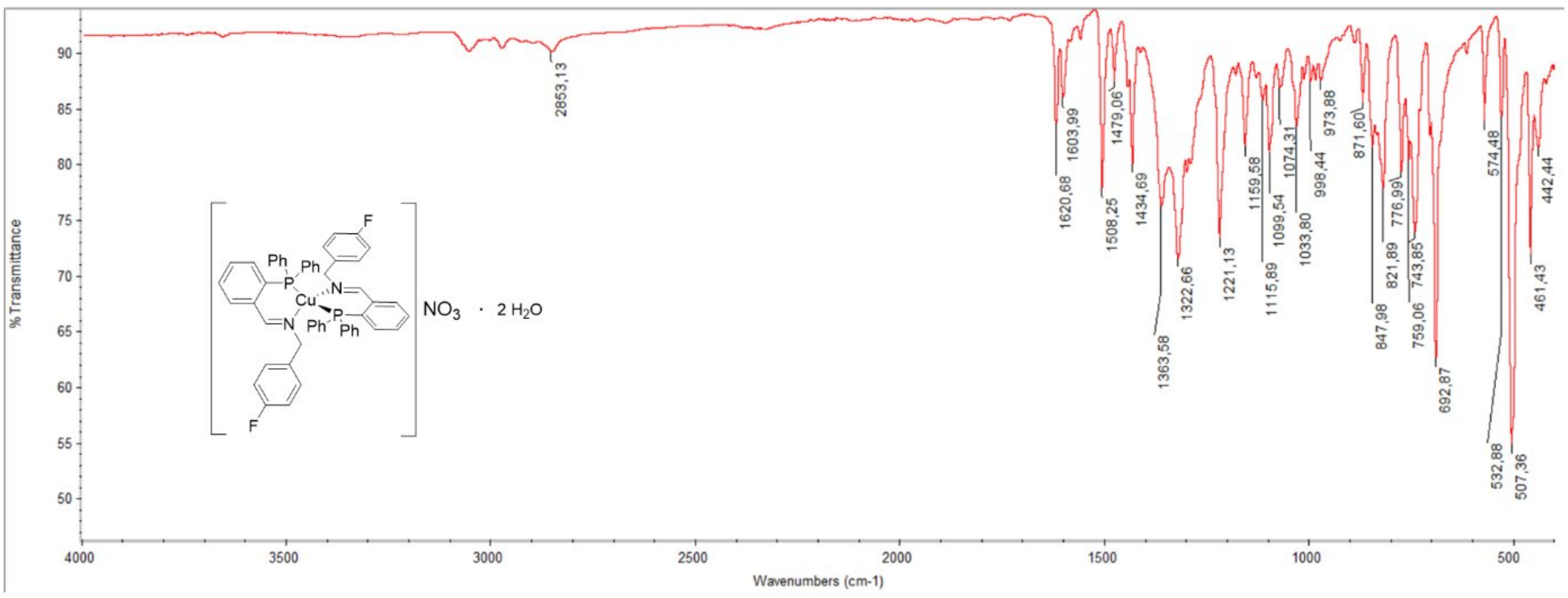

Figure S39: (top) ^{1}H NMR (400 MHz, $CDCl_3$, 298 K) and (bottom) ^{31}P NMR (162 MHz, $CDCl_3$, 298 K) spectra of **(7)**.

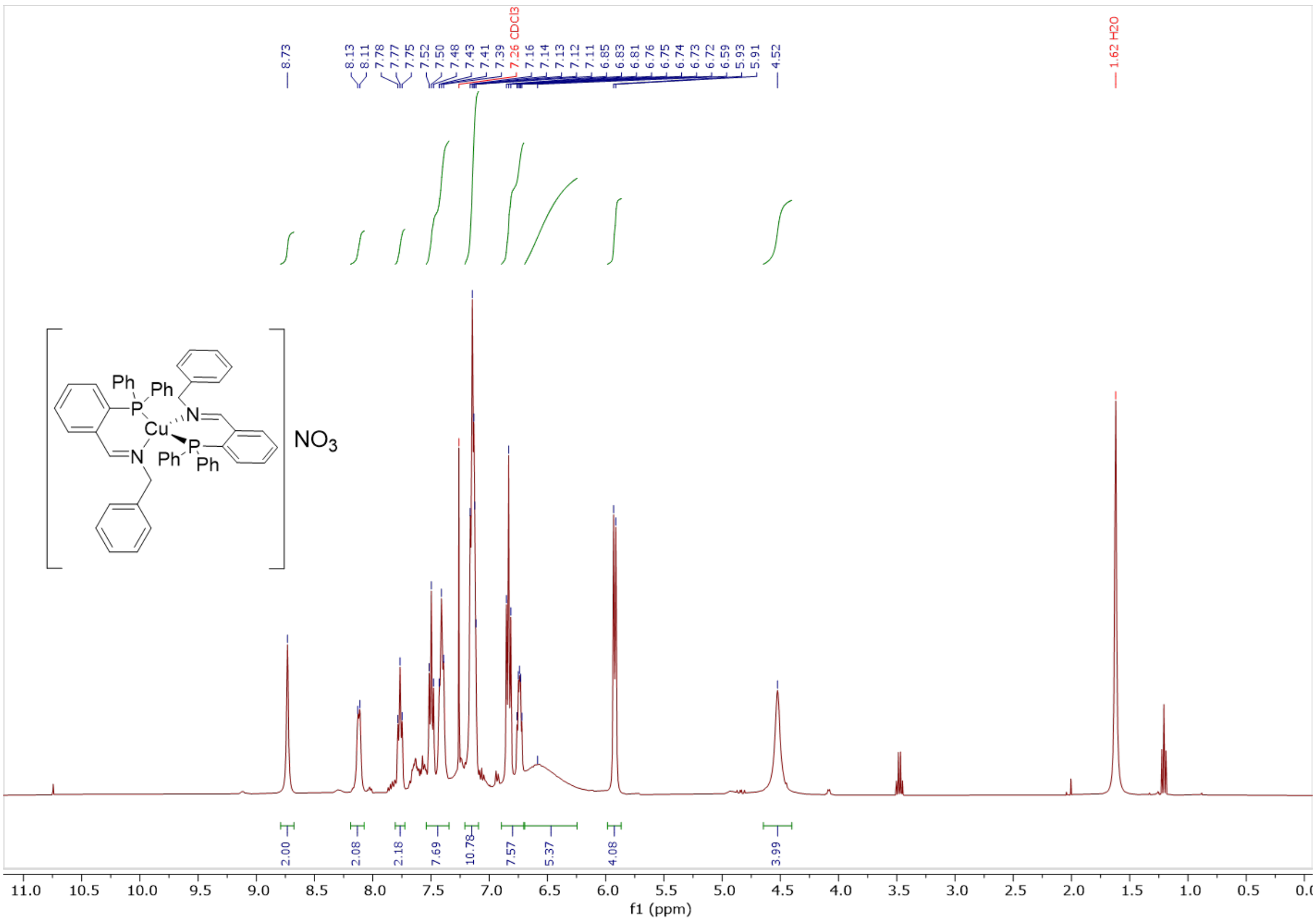

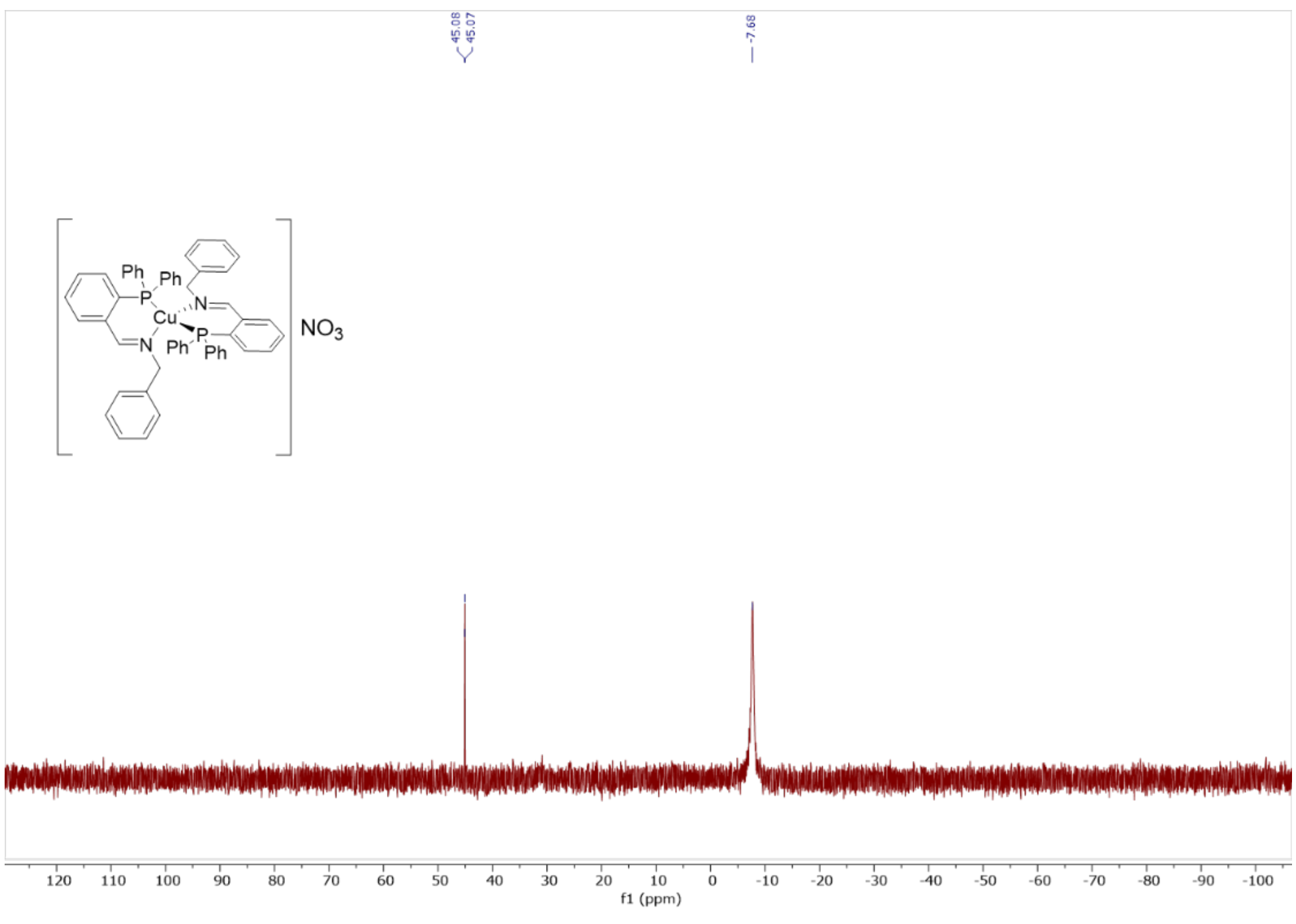

Figure S40: a) ^{1}H NMR (400 MHz, 298 K) spectrum of **(7)** recorded in DMSO-d$_6$; b) ^{31}P NMR (162 MHz, 298 K) spectrum of **(7)** in DMSO-d$_6$ measured immediately after preparation of the sample (purple), after 24 hours (blue-green), 48 hours (green) and one week (red).

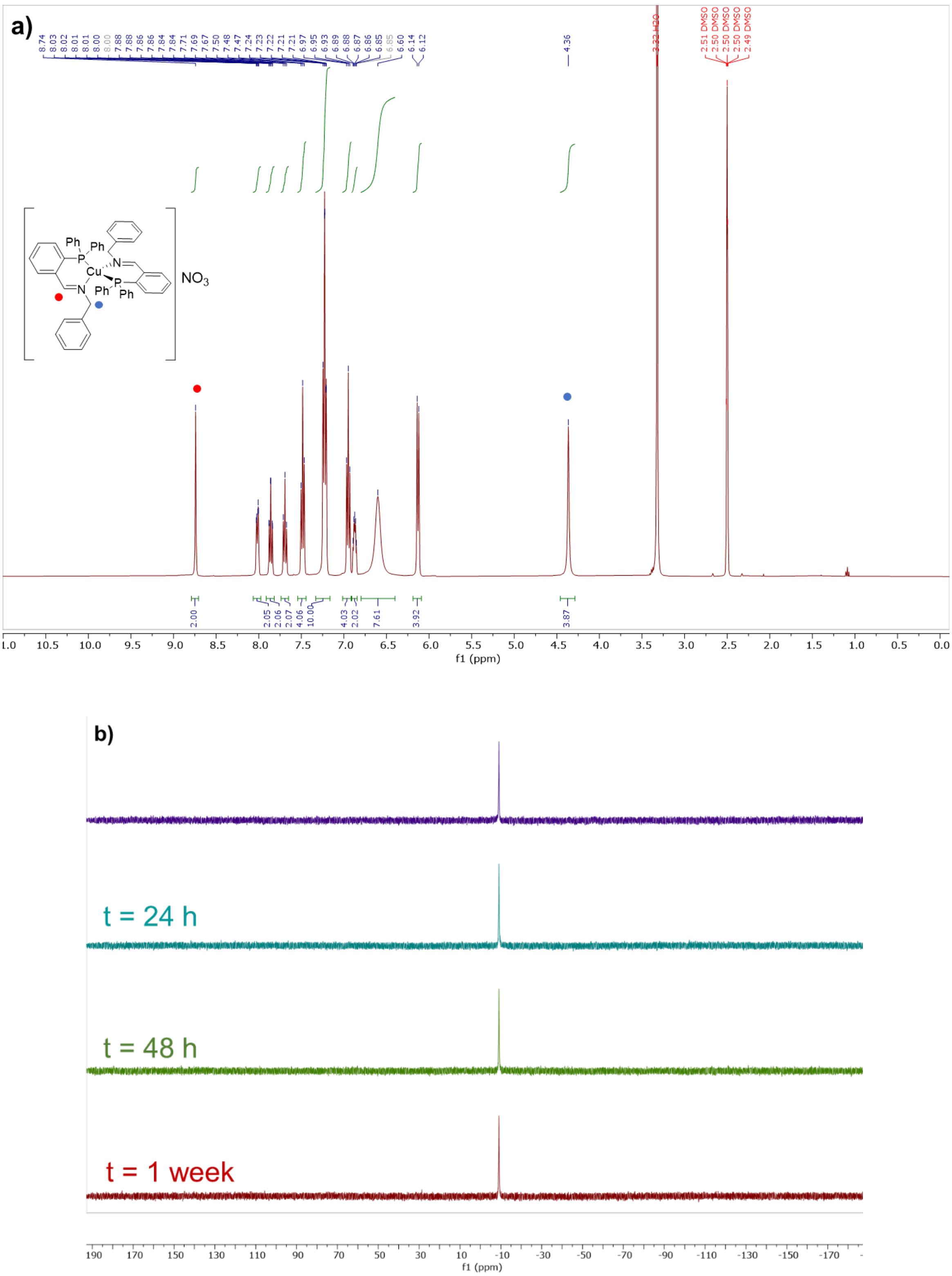

Figure S41: ^{1}H NMR (400 MHz, DMSO-d_6, 298 K) spectrum of **(8)**.

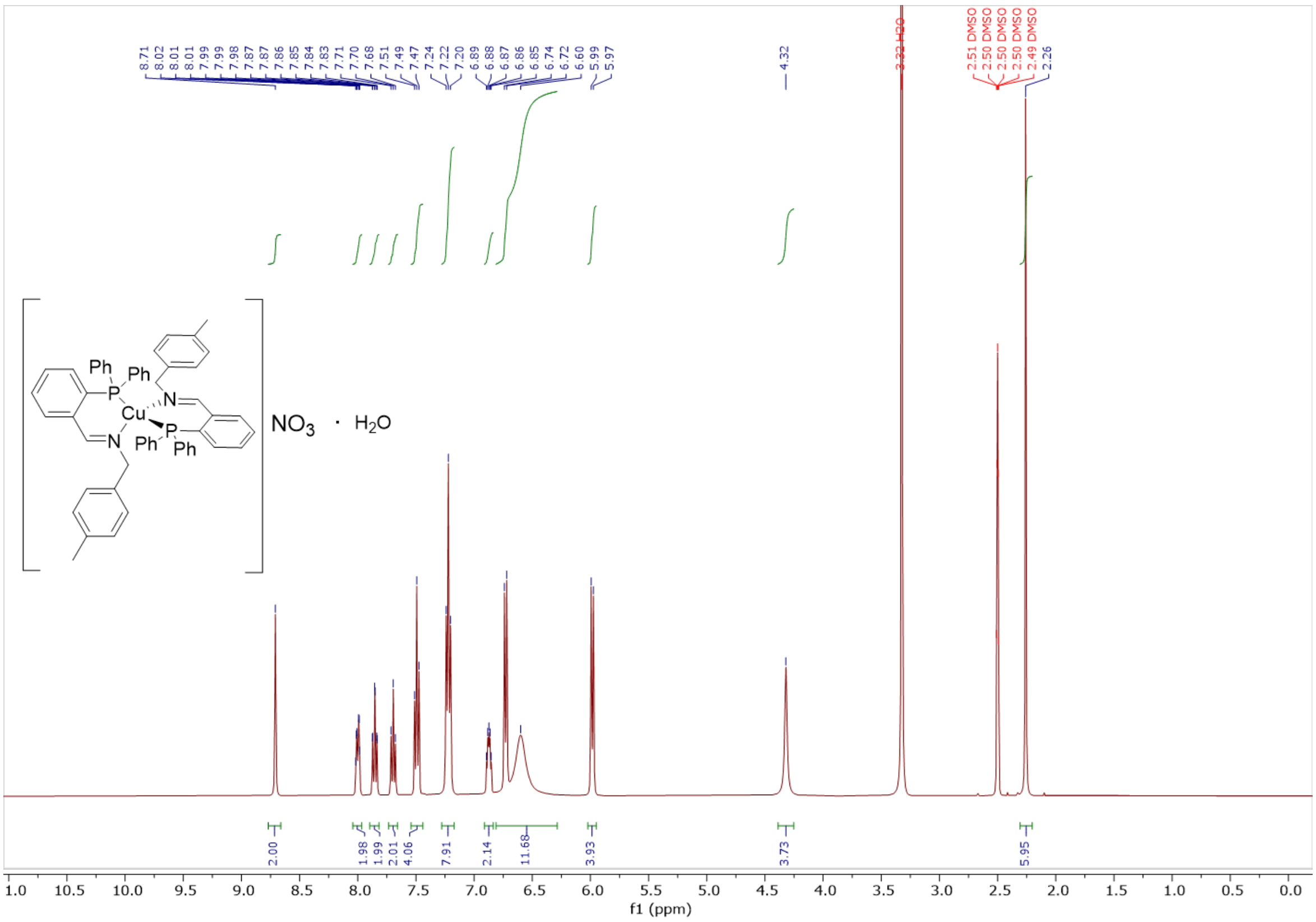

Figure S42: ^{31}P NMR (162 MHz, DMSO-d_6, 298 K) spectrum of **(8)**.

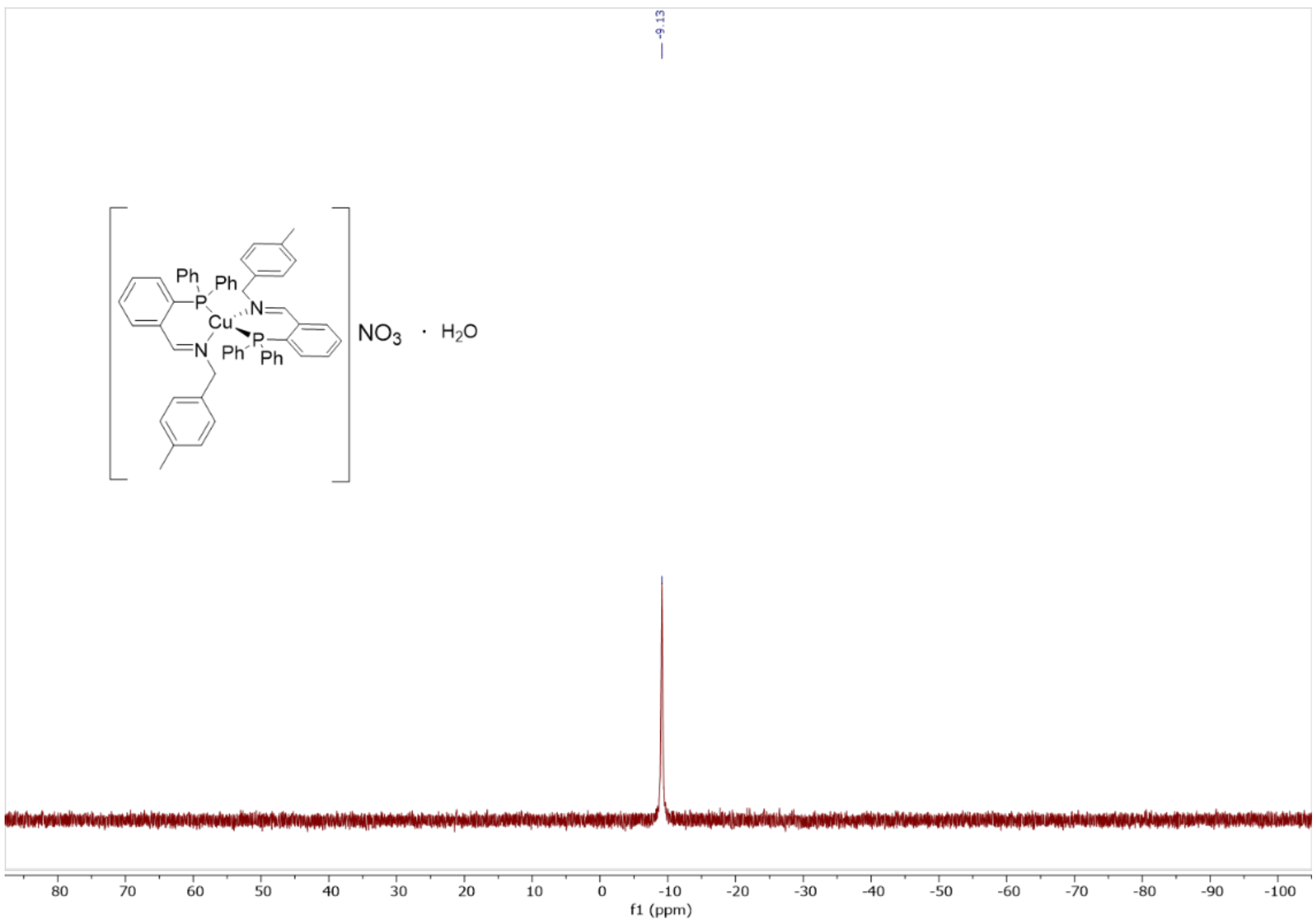

Figure S43: ^{1}H NMR (400 MHz, DMSO-d_6, 298 K) spectrum of **(9)**.

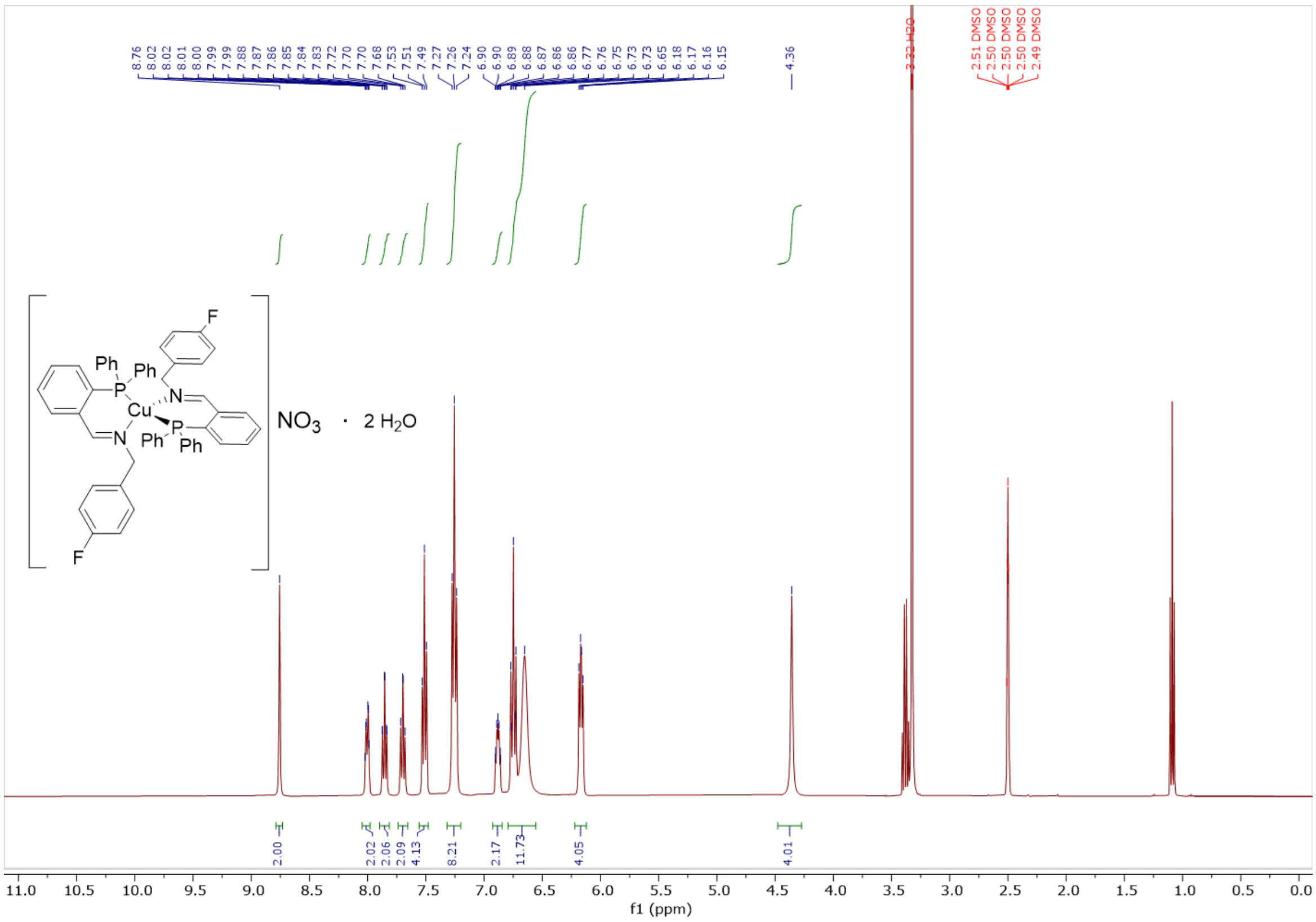

Figure S44: ^{31}P NMR (162 MHz, DMSO-d_6, 298 K) spectrum of **(9)**.

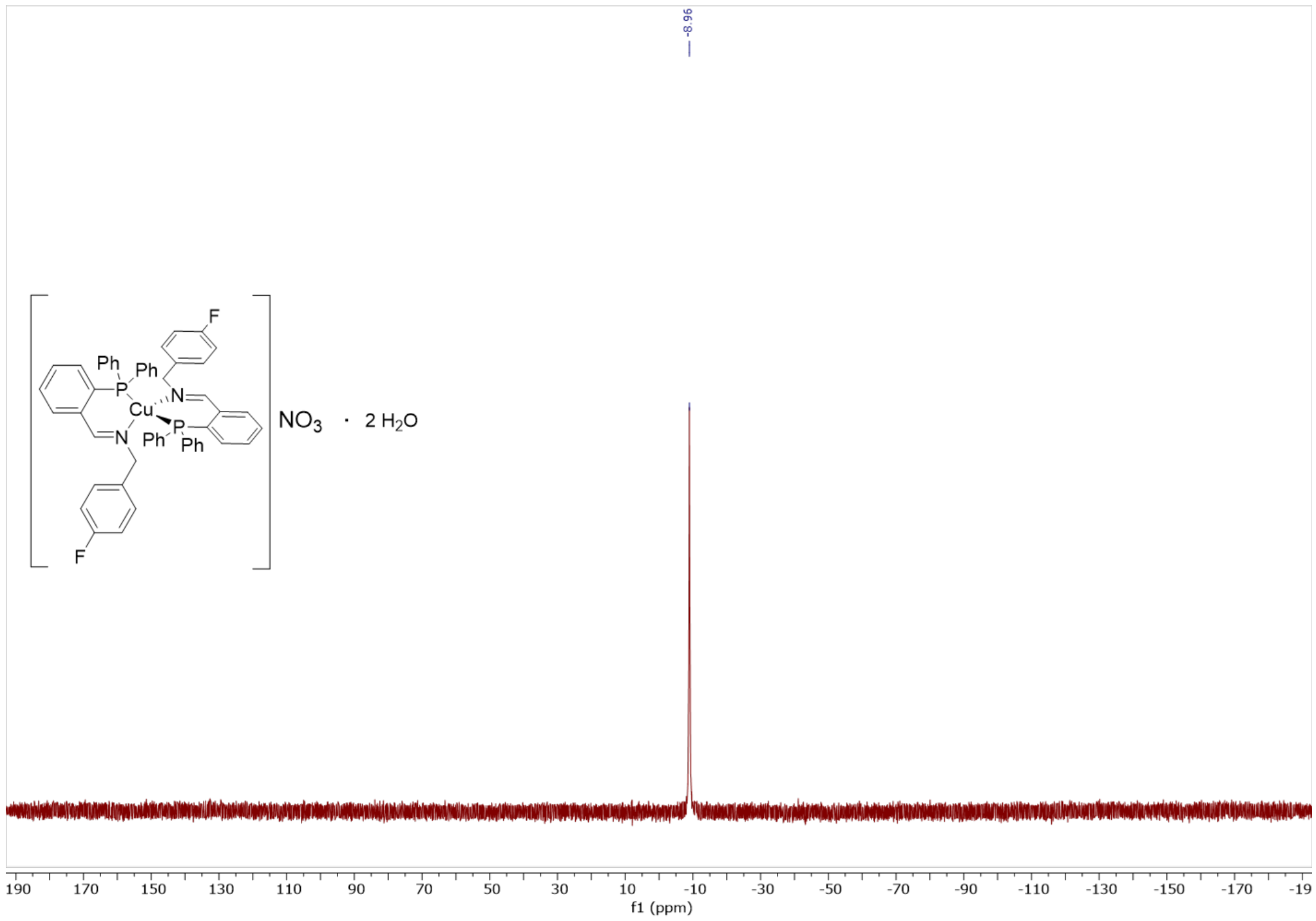

Figure S45: ESI-MS spectra (positive ions) for **(7)** (top), **(8)** and **(9)** (bottom) in acetonitrile.

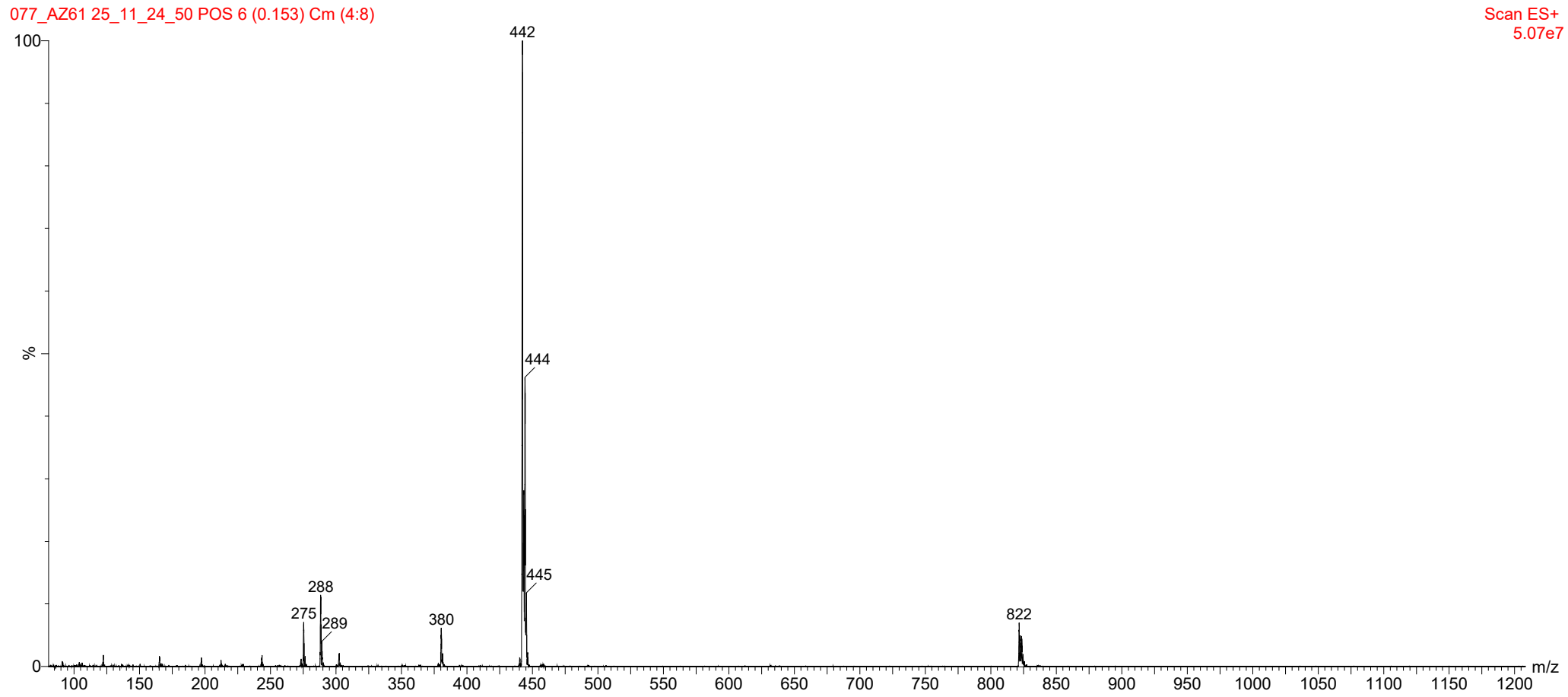

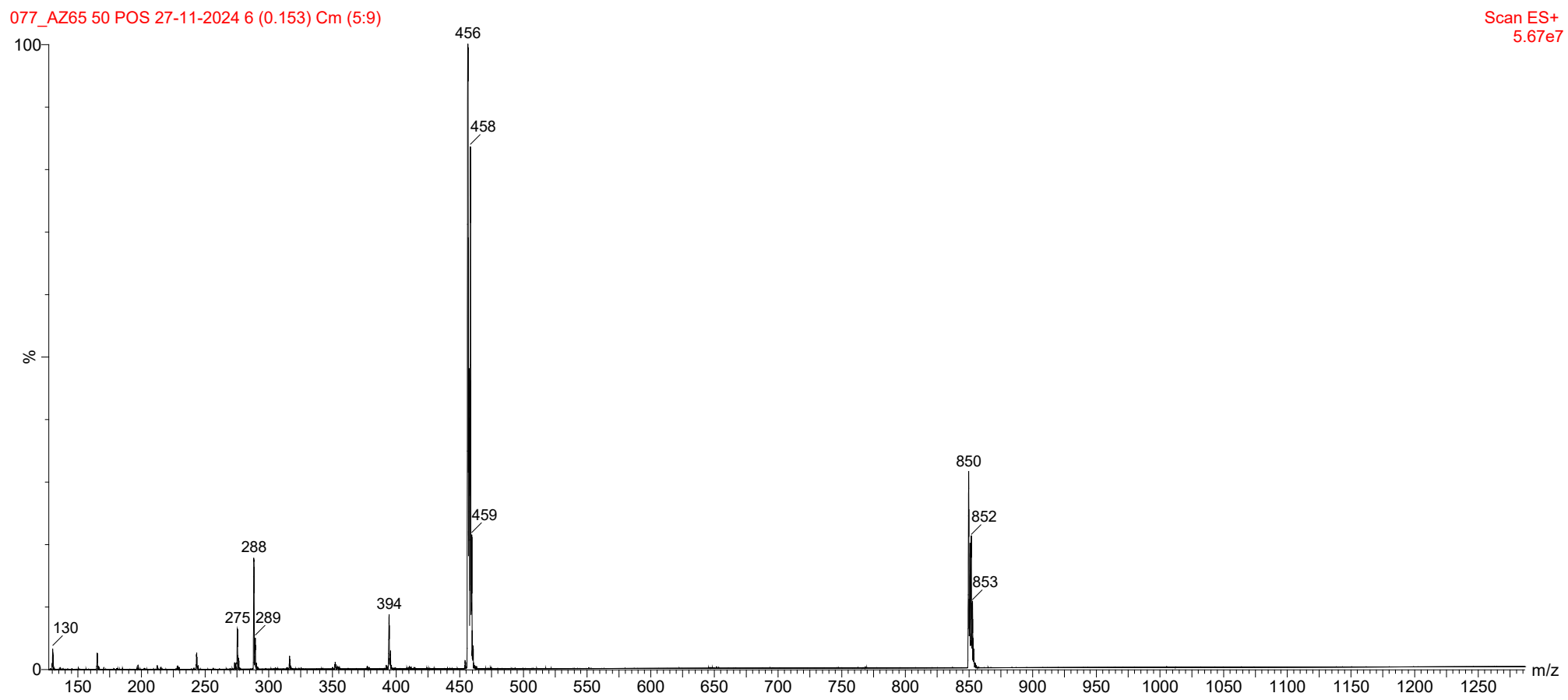

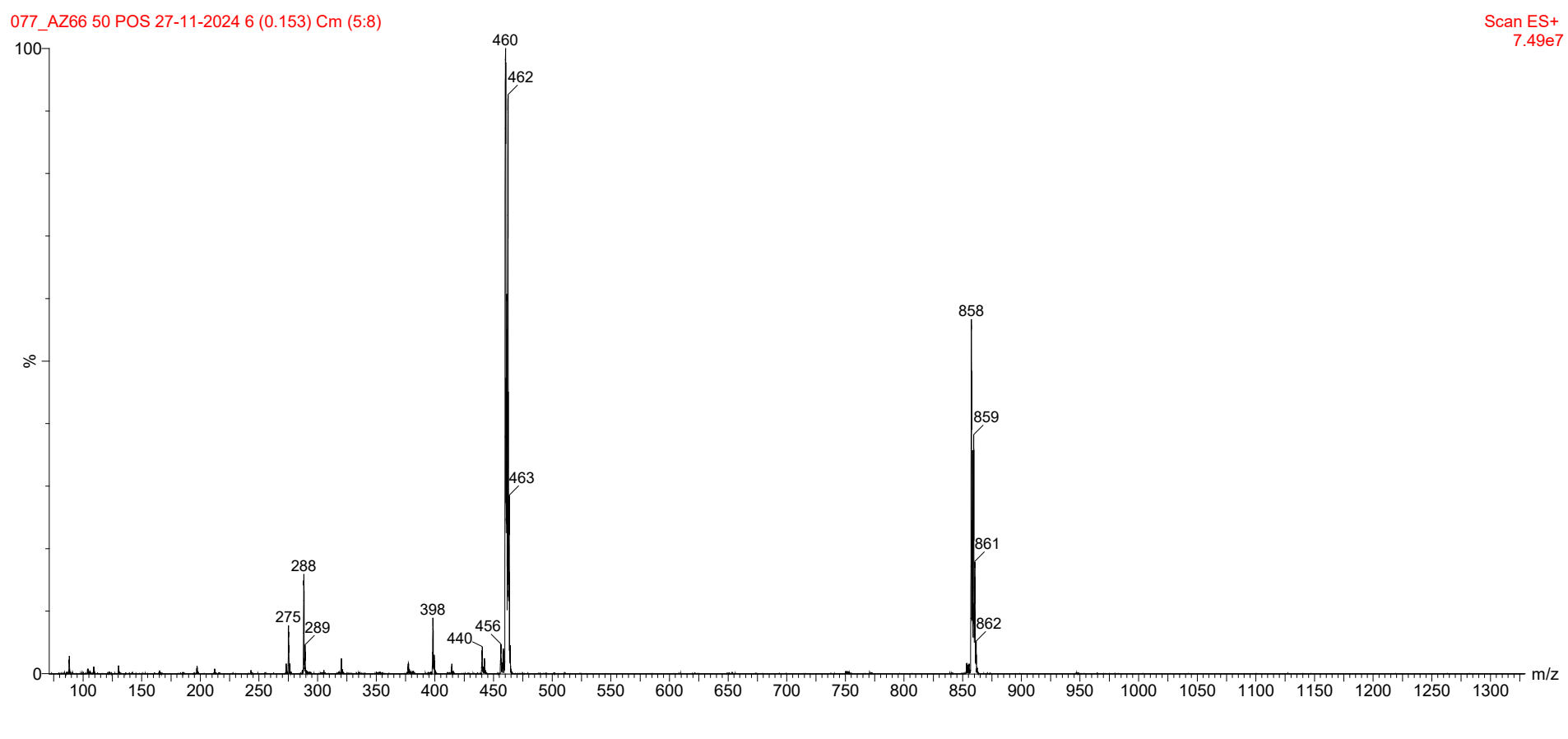

Figure S46: X-ray crystallographic data and ORTEP for compound **(7)**

**Empirical formula**	C52 H45 Cu N2 P2, N O3
**Formula weight**	885.39
**Temperature/K**	200.0
**Diffractometer/detector**	Bruker D8 Venture / PhotonII area detector
**Radiation**	CuKα (λ = 1.54178)
**Crystal system**	monoclinic
**Space group**	P21/n
**a/Å**	12.2621(5)
**b/Å**	18.4764(7)
**c/Å**	19.8598(8)
**α/°**	90
**β/°**	107.538(2)
**γ/°**	90
**Volume/Å^3**	4290.3(3)
**Z**	4
**ρcalc /g·cm^{-3}**	1.371
**μ/mm^{-1}**	1.806
**F(000)**	1844
**Θ range for data collection/°**	3.342 - 70.056
	-14<h<14
**Index ranges**	-20<k<22
	-24<l<23
**Reflections collected**	56004
**Unique reflections**	8133
**Parameters**	661
**Goodness-of-fit on F^2**	1.033
**Final R indexes [I>=2σ (I)]**	R=0.035, wR2=0.086
**CCDC Number**	2560047

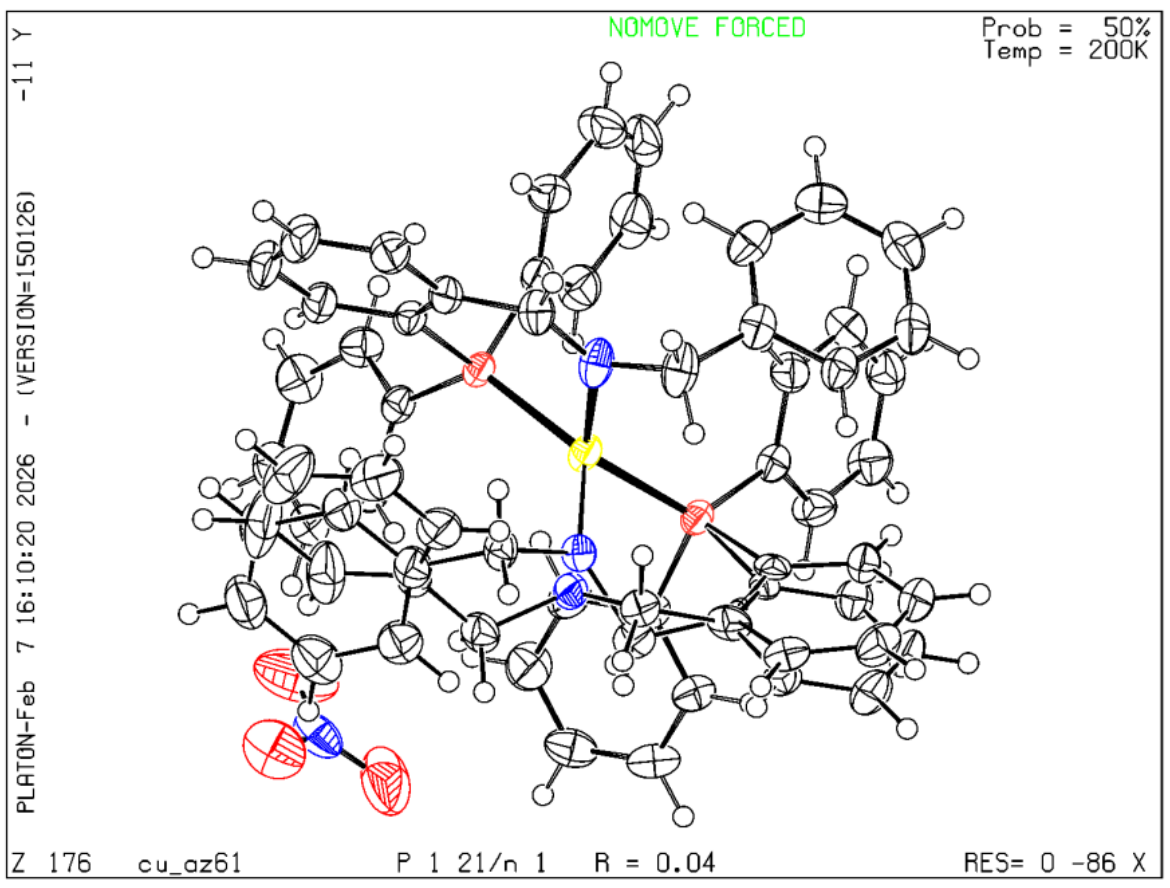

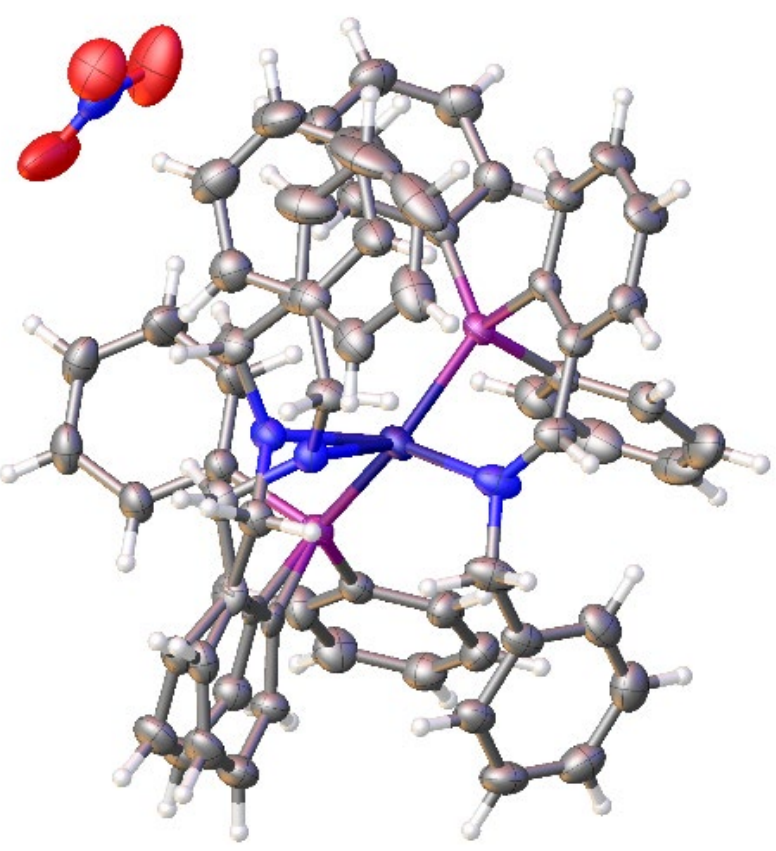

Figure S47: FT-IR spectra of **[Cu(L^{a})$_2$NO$_3$]** (in blue) and PCHO (in red).

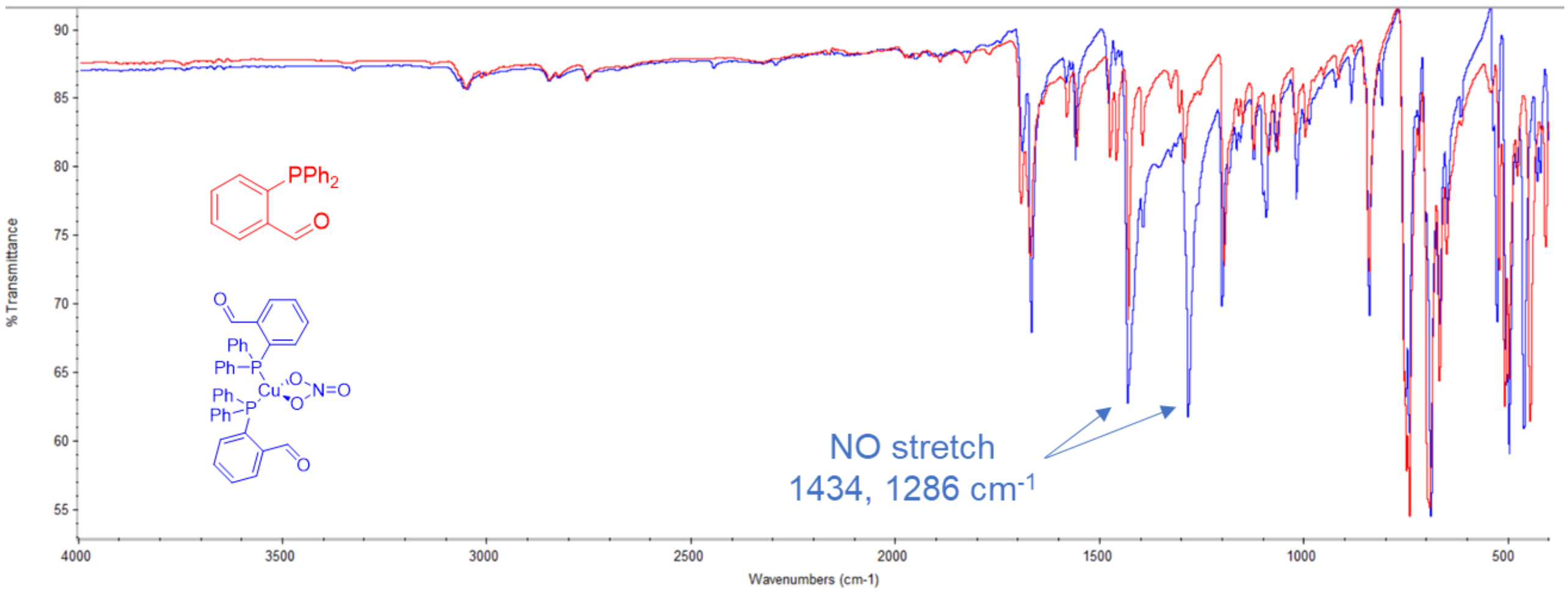

Figure S48: X-ray crystallographic data and ORTEP diagram for compound **[$Cu(L^a)_2NO_3$]**. (a) ORTEP diagram of the crystal structure of complex, drawn with 50% probability ellipsoids; (b) coordination geometry of copper(I) centre with indication of P-Cu-P and O-Cu-O angles (phosphorus atoms in light orange, nitrogen in blue, carbon in grey, hydrogens in white, oxygen in red, copper in dark orange).

**Empirical formula**	C38 H30 Cu N O5 P2
**Formula weight**	706.11
**Temperature/K**	200.0
**Diffractometer/detector**	Bruker D8 Venture / PhotonII area detector
**Radiation**	MoKα (λ = 0.71073)
**Crystal system**	monoclinic
**Space group**	C2/c
**a/Å**	9.6698(10)
**b/Å**	17.6866(17)
**c/Å**	19.3856(16)
**α/°**	90
**β/°**	95.514(4)
**γ/°**	90
**Volume/Å^3**	3300.1(5)
**Z**	4
**ρcalc /g·cm^{-3}**	1.421
**μ/mm^{-1}**	0.805
**F(000)**	1844
**Θ range for data collection/°**	3.342 - 70.056
	-12<h<12
**Index ranges**	0<k<23
	0<l<25
**Reflections collected**	36340
**Unique reflections**	4091
**Parameters**	218
**Goodness-of-fit on F^2**	1.097
**Final R indexes [I>=2σ (I)]**	R=0.070, wR2=0.121
**CCDC Number**	2560043

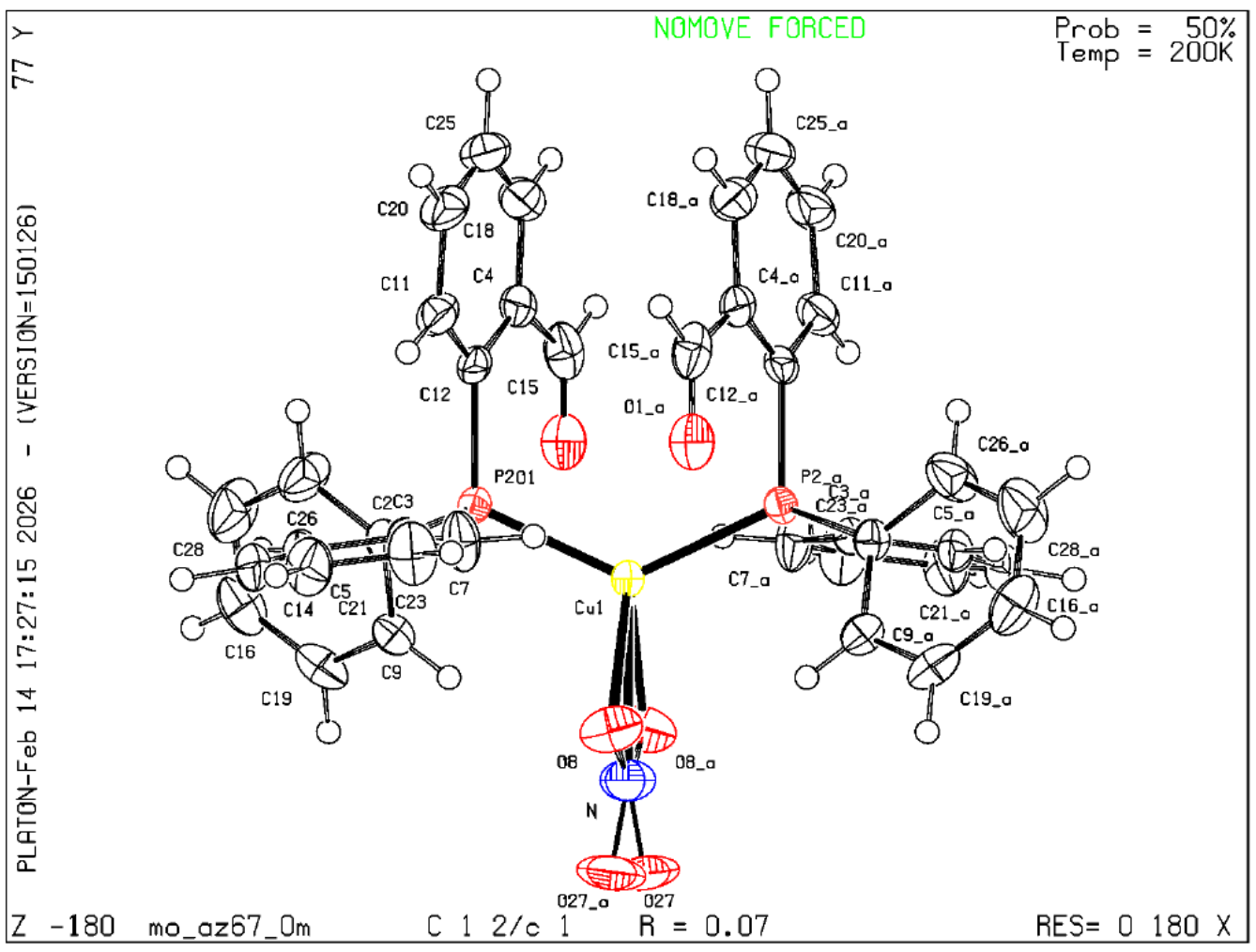

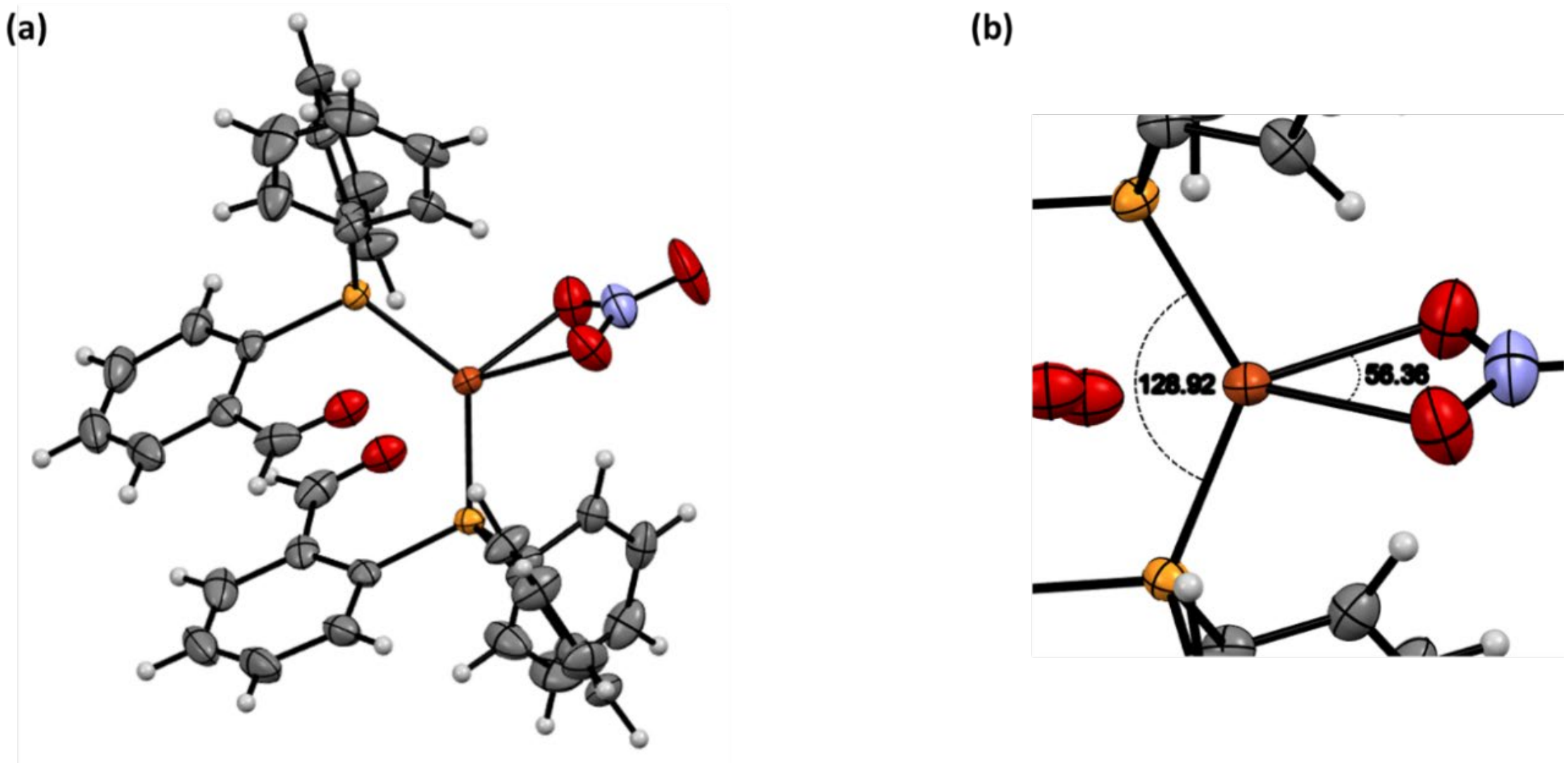

(a)
(b)
128.92
56.36

Figure S49: ^{1}H NMR (400 MHz, DMSO-d_6, 298 K) spectrum of **[Cu(L^a)$_2$NO$_3$]**.

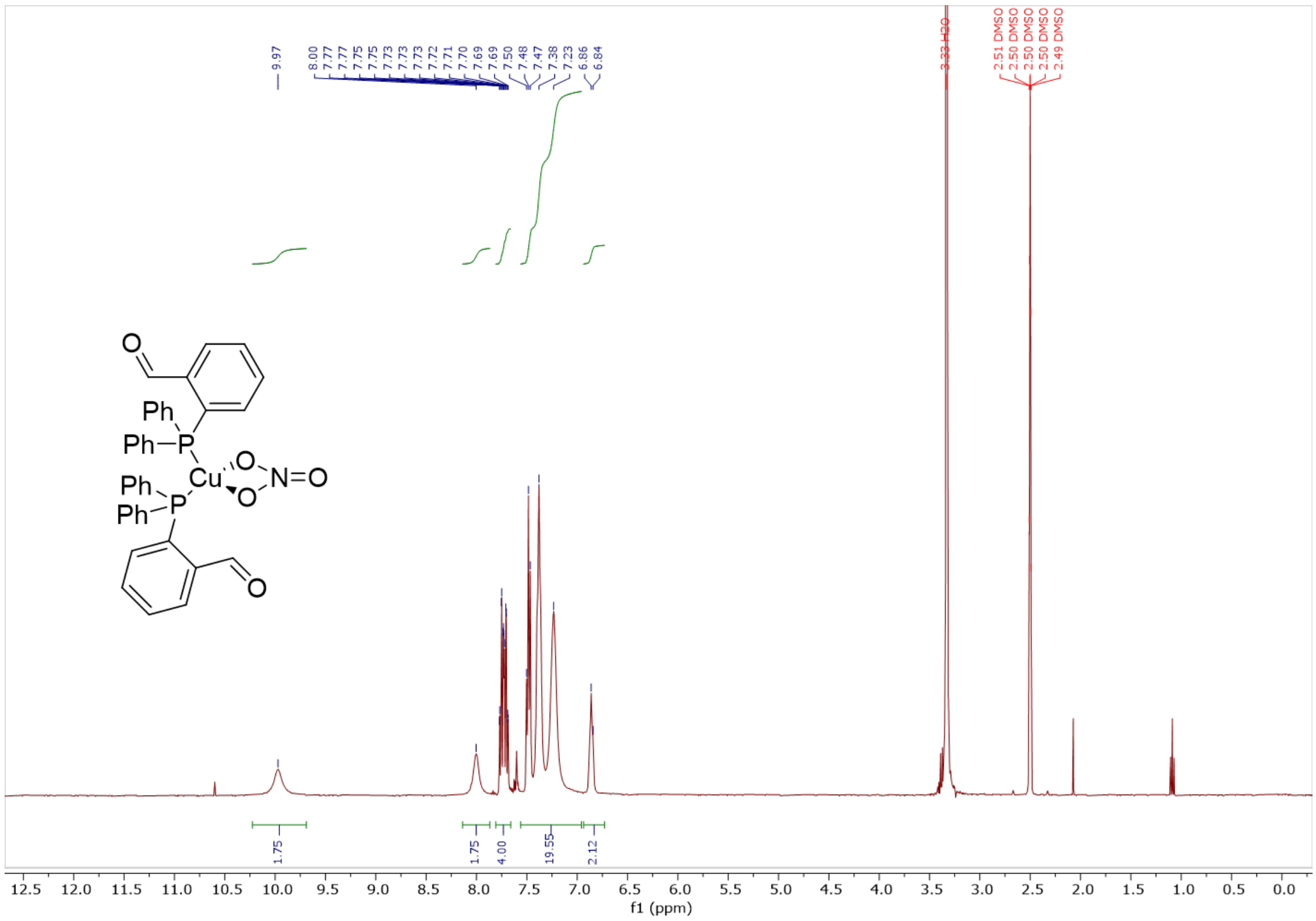

Figure S50: ESI-MS spectrum (positive ions) for **[Cu(L^a)$_2$NO$_3$]** in acetonitrile.

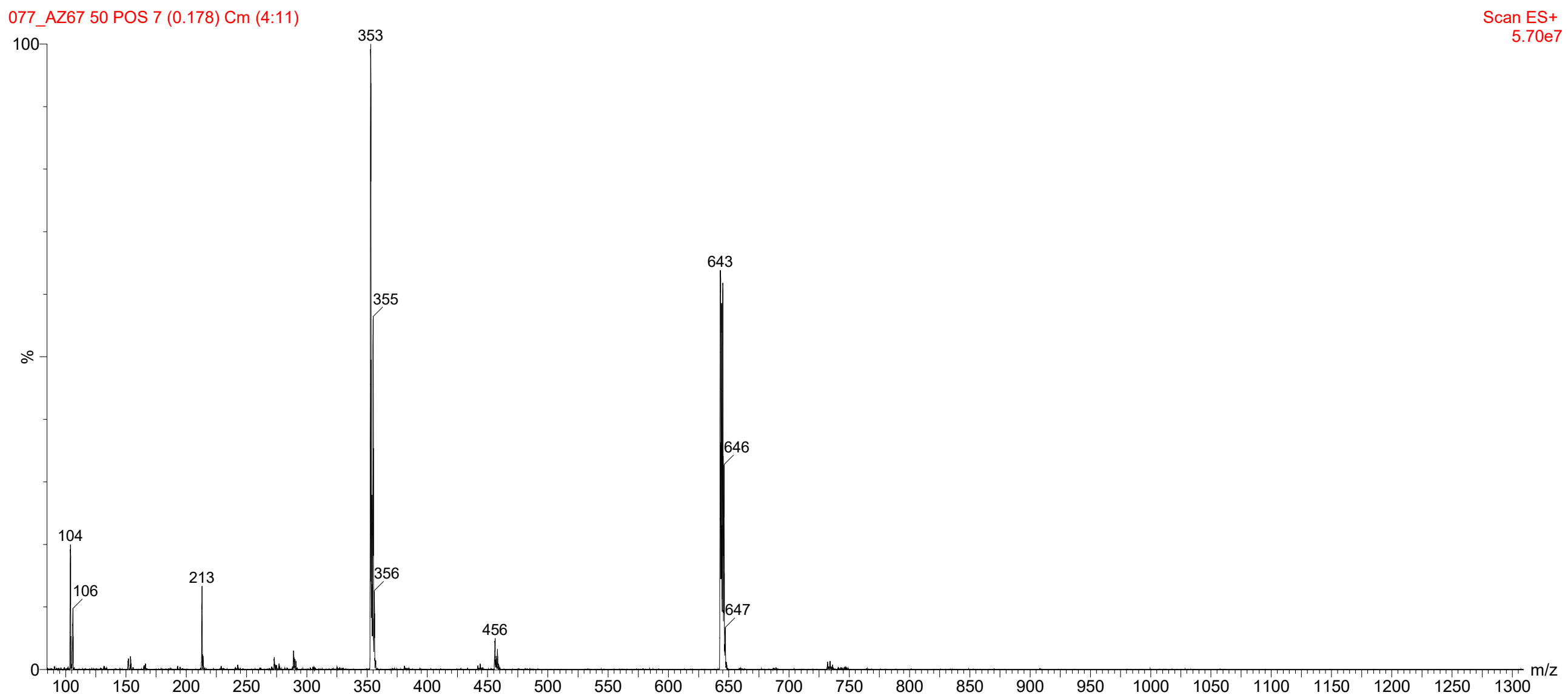

Table S51: IC_{50} and CC_{50} values (mean ± standard deviation) of the reference drug controls

	IC_{50} (µM)			CC_{50} (µM)
Reference drugs	*S. aureus*	*E. coli*	*C. albicans*	Vero cells
Ciprofloxacin	0.39 ± 0.13	1.013 ± 0.001	n.d.*	n.d.
Fluconazole	n.d.*	n.d.	0.19 ± 0.06	n.d.
Cisplatin	n.d.	n.d.	n.d.	0.99 ± 0.74

*n.d. not determined

Table S52: IC_{50} values (in µM) of copper(I) iminophosphine complexes as determined by the nonlinear regression (curve fit) obtained using GraphPad Prism version 10.0.0 for Windows, (GraphPad Software, Boston, Massachusetts USA, www.graphpad.com).

**log(inhibitor) vs. normalized response -- Variable slope**						
Best-fit values	**(4)**	**(5)**	**(6)**	**(7)**	**(8)**	**(9)**
LogIC50	1,281	1,328	1,432	1,387	1,392	1,434
HillSlope	-7,845	-7,499	-7,834	-5,606	-5,129	-7,268
**IC50**	**19,08**	**21,3**	**27,03**	**24,37**	**24,64**	**27,15**
95% CI (profile likelihood)						
LogIC50	1,153 to 1,405	1,178 to 1,489	1,294 to 1,62	1,228 to 1,543	1,241 to 1,556	1,293 to 1,606
IC50	14,21 to 25,40	15,08 to 30,83	19,68 to 42,6	16,90 to 34,88	17,44 to 35,99	19,65 to 40,41
Goodness of Fit						
Degrees of Freedom	62	42	43	46	43	43
R squared	0,9817	0,9496	0,8743	0,8694	0,8759	0,9018
Sum of Squares	2546	4789	11768	12554	11101	8588
Sy.x	6,408	10,68	16,54	16,52	16,07	14,13
Number of points						
# of X values	64	64	64	64	64	64
# Y values analyzed	64	44	45	48	45	45

Table S53: CC_{50} values (in μM) of copper(I) iminophosphine complexes as determined by the nonlinear regression (curve fit) obtained using GraphPad Prism version 10.0.0 for Windows, (GraphPad Software, Boston, Massachusetts USA, www.graphpad.com).

**log(inhibitor) vs. normalized response -- Variable slope**						
Best-fit values	**(4)**	**(5)**	**(6)**	**(7)**	**(8)**	**(9)**
LogCC50	0,6291	0,7536	0,7196	0,5626	0,563	0,7014
HillSlope	-2,754	-3,536	-3,94	-4,915	-8,862	-4,603
**CC50**	**4,257**	**5,67**	**5,243**	**3,652**	**3,656**	**5,028**
95% CI (profile likelihood)						
LogCC50	0,3808 to 0,7004	0,4934 to 0,83	0,4737 to 0,8	0,2839 to 0,65	0,2967 to 0,7(	0,4704 to 0,8462
CC50	2,403 to 5,017	3,114 to 6,788	2,977 to 6,86	1,923 to 4,468	1,980 to 5,05:	2,954 to 7,018
Goodness of Fit						
Degrees of Freedom	22	22	22	22	22	22
R squared	0,9517	0,9694	0,9802	0,9677	0,9791	0,9898
Sum of Squares	1898	1360	966,1	1386	994,6	519,1
Sy.x	9,287	7,863	6,627	7,937	6,724	4,857
Number of points						
# of X values	24	24	24	24	24	24
# Y values analyzed	24	24	24	24	24	24

Figure S54: Dose–response curves of the complexes obtained in Caco-2 cells generated by plotting the percentage of the cell viability relative to untreated controls and as a function of the logarithm of the compound concentration (μM). CC_{50} values are indicated by the intersection with the 50% response level.

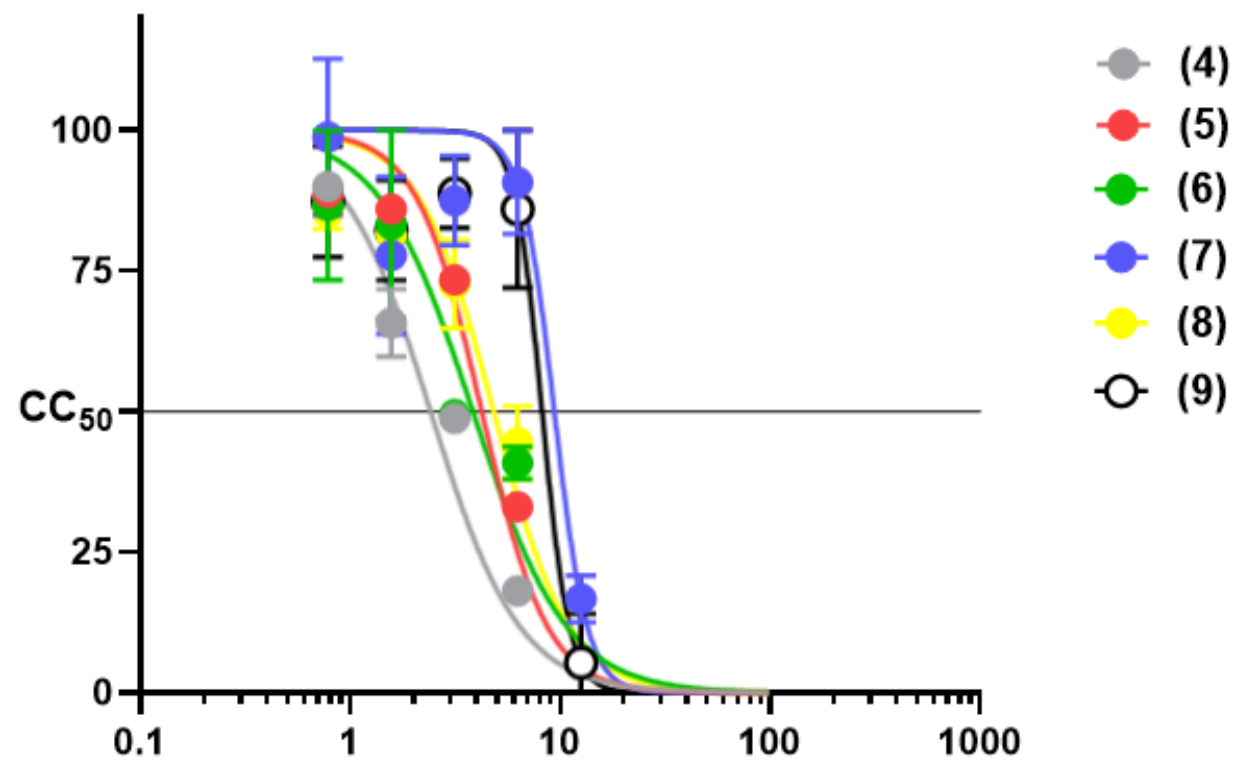

Table S55: CC_{50} values determined in Caco-2 cells and hemolytic activity (%) measured at the corresponding CC_{50} concentrations.

**Complexes**	**CC_{50} (μM)**	**Hemolytic activity (%)**
**(4)**	2.07 (1.79 – 3.25)	< 5
**(5)**	3.68 (3.16 – 5.60)	< 5
**(6)**	3.99 (2.65 – 5.38)	< 5
**(7)**	8.98 (7.89 – 9.05)	< 5
**(8)**	4.49 (3.73 – 6.00)	< 5
**(9)**	9.49 (8.54 – 11.01)	12.01 ± 2.83
